# An Evaluation of Fish Tissue Monitoring Alternatives for Mercury and Selenium: Fish Muscle Biopsy Samples Versus Homogenized Whole Fillets

**DOI:** 10.1007/s00244-021-00872-w

**Published:** 2021-07-30

**Authors:** Leanne L. Stahl, Blaine D. Snyder, Harry B. McCarty, Tara R. Cohen, Kenneth M. Miller, Mark B. Fernandez, John C. Healey

**Affiliations:** 1 OW/Office of Science and Technology, U.S. Environmental Protection Agency, 1200 Pennsylvania Avenue, NW (MC 4305T), Washington, DC 20460 USA; 2 Center for Ecological Sciences, Tetra Tech, Inc., 10711 Red Brook Boulevard, Suite 105, Owings Mills, MD 21117 USA; 3 A General Dynamics Information Technology Company, CSRA, LLC, 6361 Walker Lane, Alexandria, VA 22310 USA

## Abstract

Fish contaminant studies with human health protection objectives typically focus on muscle tissue, recognizing that fillets are the commonly consumed tissue fraction. Muscle biopsy punch sampling for mercury analysis has recently been used as an alternative to harvesting fish for fillets; however, there is limited information comparing fillet plug results to whole fillet results. This study was conducted to address that data gap and to test the applicability of plugs for monitoring associated with United States Environmental Protection Agency’s fish tissue-based mercury and selenium water quality criteria. The mercury phase included 300 fillet homogenates and 300 field-extracted plug samples from 60 fish, and the selenium phase included 120 fillet homogenates and 120 plugs from 30 fish. Both phases showed that there were no statistically significant differences between fillet plug and homogenized fillet results at the community level; however, a selenium plug monitoring alternative must employ a sufficiently sensitive analytical method and consider total solids. Plug and fillet sampling alternatives have inherent advantages and disadvantages. Fillet sampling provides sufficient mass to consider multiple contaminants but requires fish to be harvested. Plug sampling only provides adequate mass for a single analyte but may allow fish survival, although additional research is needed on survival following plug removal.

**Supplementary Information:**

The online version contains supplementary material available at 10.1007/s00244-021-00872-w.

Monitoring chemical contaminant levels in the aquatic environment continues to be important for the characterization of water resource conditions, identification of associated impacts, and protection of human and ecological health. Aquatic organisms may bioaccumulate contaminants to levels that are up to six orders of magnitude higher than concentrations detected in the water column (USEPA 2000). Therefore, aquatic biota, including fish, serve as important indicators for water quality and human health assessments. In the United States (USA), many states, territories, and Native American tribes routinely conduct chemical contaminant analyses of fish tissue as part of their comprehensive water quality monitoring programs and most provide safe eating guidelines and/or issue fish consumption advisories to inform the public about recommended safe levels of consumption for fish caught in local waters (USEPA 2013).

Fish tissue contaminant studies with human health protection objectives typically focus on fish species that are important to commercial, recreational, and subsistence fisheries and on the most commonly eaten tissue fraction (i.e., fillets) of those species (USEPA 2000; Stahl et al. 2009). USEPA’s *Guidance for Assessing Chemical Contaminant Data for Use in Fish Advisories* (USEPA 2000) recommends the use of homogenized fish fillets for both screening-level and intensive fish contaminant monitoring studies because fillets are the tissue fraction typically consumed by humans, and fillet samples provide sufficient tissue mass for the analysis of multiple contaminants. In both of those cases, whole fish must be killed to remove and homogenize the entire fillets.

Researchers have considered other tissue collection methods, often for mercury analysis alone, to find an alternative to killing the entire fish for analysis. Nearly 50 years ago, Uthe (1971) tested the use of a biopsy needle for the extraction of muscle tissue (i.e., 40-mg samples) from live fish for mercury analysis. Crawford et al. (1977) published a technique for taking skeletal muscle samples from live adult Steelhead (*Oncorhynchus mykiss*) (for genetic comparisons) using a dermal biopsy punch to collect a 1-cm^3^ tissue sample and release the fish alive. Rolfhus et al. (2008) and Piraino and Taylor (2013) tested whether fish fins, i.e., those typically clipped to mark released fish during population studies, could be used as a non-lethal approach for monitoring mercury in fish. Heltsley et al. (2005) assessed the use of the adipose fins of certain fish species to evaluate the body burdens of persistent organic compounds. Tissue collection alternatives that avoid a need to harvest fish (i.e., for filleting and homogenization) are advantageous for monitoring contaminant levels in rare, threatened, or endangered species. Waddell and May (1995) used fish muscle biopsy punch (or fillet plug) samples to analyze selenium levels in the endangered Razorback Sucker (*Xyrauchen texanus*), Osmundson et al. (2000) collected fillet plugs from the endangered Colorado Pikeminnow (*Ptychocheilus lucius*) for selenium analysis, and Osmundson and Lusk (2019) used fillet plug samples to assess mercury levels in endangered Colorado Pikeminnows.

The choice of tissue sample type for contaminant analysis ultimately depends on study objectives, target chemicals, and tissue volumes needed for analysis. Advances in analytical techniques over time have resulted in the reduction of tissue volumes required for some contaminants. As a result, muscle biopsy punch sampling has become increasingly popular among researchers and natural resource agencies for assessing contaminants in fish, particularly mercury and selenium (Cizdziel et al. 2002; Baker et al. 2004; Peterson et al. 2005; Bauch 2007; May and Brumbaugh 2007; Schmitt and Brumbaugh 2007; May et al. 2013; Ackerson et al. 2014; Sun et al. 2017; Knight et al. 2019). Despite the level of interest and testing of fillet plug sampling and analysis, Knight et al. (2019) noted that this sampling alternative has not been widely implemented in routine monitoring due, at least in part, to concerns about the variability of contaminant concentrations in fish muscle tissue. Concerns also remain about the latent mortality of fish after fillet plug removal, whether the small sample volumes and the tissue extraction process affect precision, and whether plug results are comparable to whole fillet results.

## Study Objectives and Sampling Design

The U.S. Environmental Protection Agency (USEPA) began collecting fish muscle biopsy plug samples (fillet plugs), nationwide, from whole fish at river and stream sites during the 2013–2014 National Rivers and Streams Assessment (NRSA) as a cost-effective alternative to filleting and homogenizing fish to obtain mercury data. At that time, there was limited information on the degree to which fillet plug analysis could serve as a reliable alternative to the traditional approach of homogenizing and analyzing whole fillet tissue to monitor mercury concentrations in fish. USEPA’s use of fillet plugs for mercury analysis was expanded to include plug sampling in the Great Lakes and the nearshore coasts of the contiguous USA during the 2015 National Coastal Condition Assessment, and again at river and stream sites during the 2018–2019 NRSA. In support of those national and regional probabilistic studies, USEPA designed and conducted the Fish Plug Evaluation Study (FPES) in an effort to increase the knowledge about whether fillet plug mercury results are representative of homogenized whole fillet (hereafter referred to as “homogenized fillet”) results. The FPES also addressed the potential use of fillet plug samples for selenium surveillance monitoring in freshwater in association with the updated national Clean Water Act Section 304(a) aquatic life ambient water quality criterion for selenium (USEPA 2016a), including an evaluation of wet-weight and dry-weight concentrations. The specific objectives of this study were to assess the comparability of mercury and selenium concentrations in fillet plugs versus homogenized fillet samples and to test the applicability of plug sampling and analysis for conducting surveillance monitoring associated with USEPA’s fish tissue-based mercury and selenium water quality criteria (USEPA 2001b, 2016a).

The FPES design included two phases, the mercury phase and the selenium phase, initiated in June 2017 and May 2018, respectively. The study focused on two waterbody types (i.e., the Great Lakes and eastern US rivers) and six target fish species that are commonly caught and consumed by humans. Table 1 presents a summary of the sampling design. Ultimately, the study design objectives were to collect 60 whole fish and 300 field-extracted fillet plug samples for the mercury phase and 30 whole fish and 120 field-extracted fillet plug samples for the selenium phase (plus an additional 120 fillet plug samples for solids determination).

**Table 1:** Fish plug evaluation study design summary

Design element	Mercury phase	Selenium phase	Description
Waterbody types	2	2	Great Lakes and East Coast Rivers
Sampling sites and fish species collected	6	6	Lake Michigan, Lake Trout; Lake Erie, Walleye; Lake Ontario, Chinook Salmon; Anacostia River, Blue Catfish; Potomac River, Largemouth Bass; St. Lawrence River, Smallmouth Bass
Fish collected per site	10	5	Collection of single specimen fish samples per site
Fish tissue sample types	2	2	Fillet plug samples (two plugs per sample) and homogenized fillet tissue samples
Replicates per sample type	5	4	Number applies to each individual fish sample
Total fillet plug samples analyzed	300	120	Sampling sites (6) x fish collected per site x replicates per sample type
Total homogenized fillet tissue samples analyzed	300	120	Sampling sites (6) x fish collected per site x replicates per sample type
Total fillet samples analyzed	600	240^a^	Sampling sites (6) x fish collected per site x sample types (2) x replicates per sample type

^a^An additional 120 single-plug fillet samples were analyzed for percent solids to convert wet-weight concentrations to dry-weight concentrations

## Methods

### Sample Collection and Preparation

**Fig. 1:**
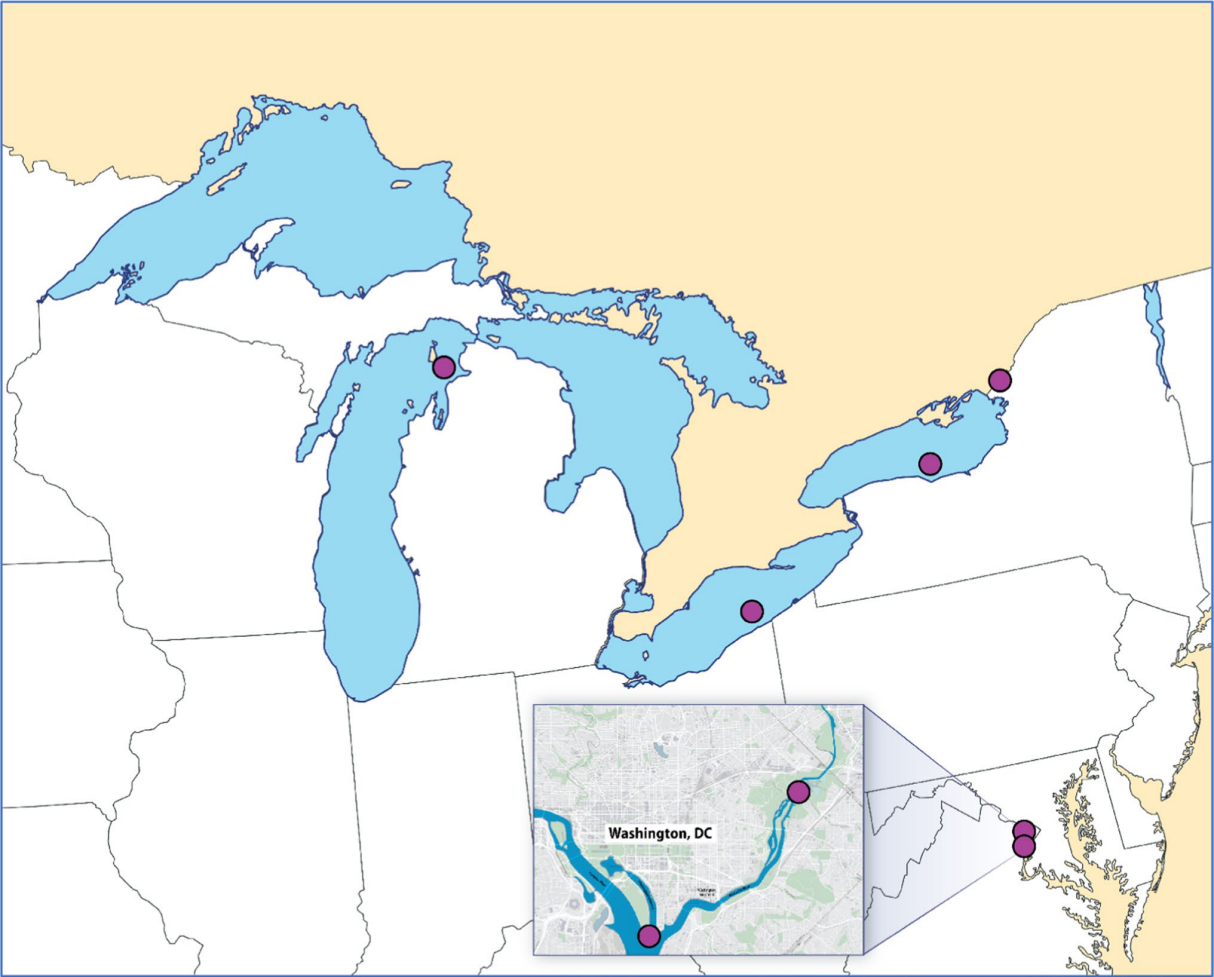
Fish Plug Evaluation Study sampling locations

The field sampling team used boat electrofishing and hook-and-line methods to collect the target species and numbers of whole fish for both the mercury and selenium phases of the study. Fish sampling was conducted in two waterbody types, the Great Lakes (i.e., Lake Michigan, Lake Erie, and Lake Ontario) and eastern US rivers (i.e., Anacostia River, Potomac River, and St. Lawrence River) (Fig. 1), in order to ensure collection of a variety of fish species and to encounter a range of contaminant concentrations. Individual whole fish were collected from each waterbody type to provide fillet plug and homogenized fillet tissue samples for mercury and selenium analyses. Ten adult specimens were collected from each of the six waterbodies for mercury analysis, with one target species per waterbody (Table 1). Target species for the Great Lakes were Lake Trout (*Salvelinus namaycush*), Walleye (*Sander vitreus*), and Chinook Salmon (*Oncorhynchus tshawytscha*). Target species for rivers were Largemouth Bass (*Micropterus salmoides*), Smallmouth Bass (*Micropterus dolomieu*), and Blue Catfish (*Ictalurus furcatus*). The mercury phase sampling effort was completed in September 2017 and yielded 60 individual whole fish samples for analysis. For the selenium phase, five specimens of each of the six target species mentioned above (i.e., one target species collected per sampling location) were collected from the three designated Great Lakes and three rivers in June through August 2018 and yielded 30 individual whole fish samples. All retained specimens were of suitable size to provide adequate biomass for all fillet plug and homogenized fillet samples needed for the FPES. Each fish of the selected target species was measured to the nearest millimeter, total length. Field-extracted fillet plugs were removed from the fish onsite, whole fish were individually wrapped in solvent-rinsed aluminum foil and food grade polyethylene tubing, and the whole fish samples and fillet plugs were transported to the Tetra Tech laboratory in Owings Mills, MD (where fillets were removed and homogenized).

**Fig. 2:**
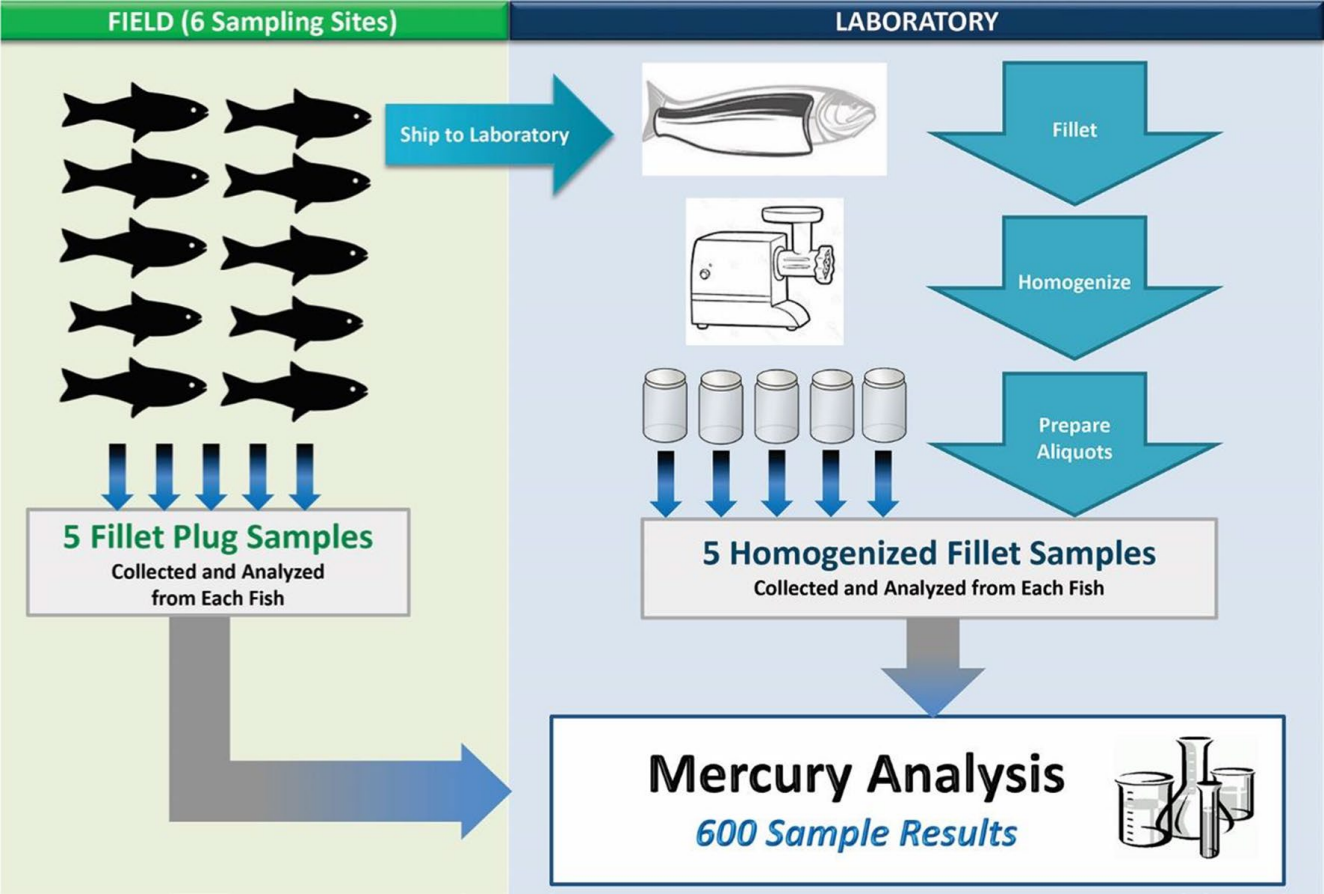
Mercury phase fillet plug sample and homogenized fillet sample collection and preparation summary

**Fig. 3:**
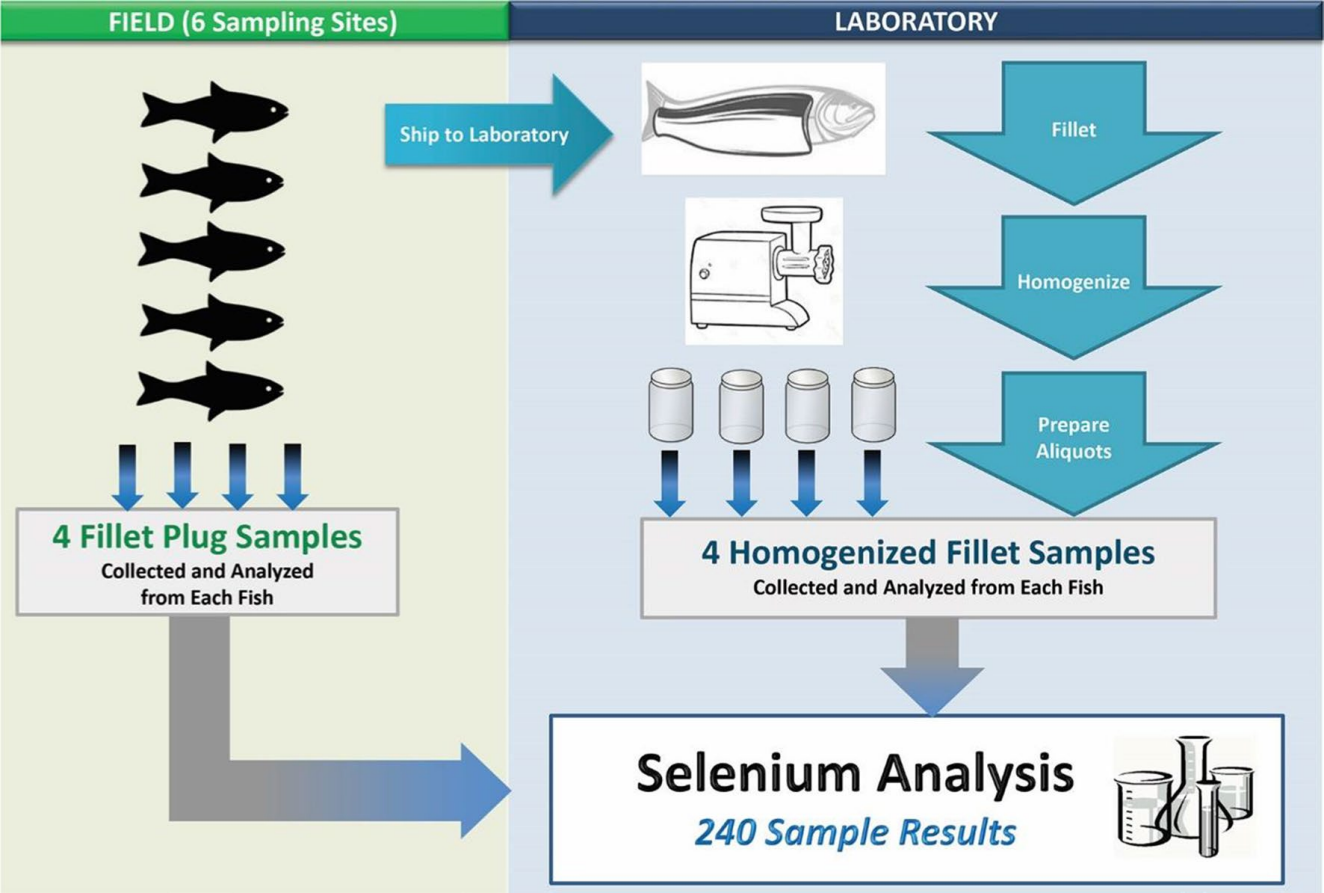
Selenium phase fillet plug sample and homogenized fillet sample collection and preparation summary

The field fillet plug removal process began with the removal of scales (using a sterile scalpel) from a small area on the left side dorsal area of each fish between the dorsal fin and the lateral line. An 8-mm dermal biopsy punch was inserted into that area of the dorsal muscle, cutting the skin and muscle tissue. The full depth of the punch was filled with muscle tissue (which yielded an average of 0.5 g of fillet tissue when filled) to ensure collection of sufficient tissue plug biomass for mercury or selenium analyses. A pipette bulb was placed on the handle of the biopsy punch and squeezed to force the plug into a sterile 20-ml scintillation vial. This process was repeated so that each field-extracted plug sample for both the mercury phase and selenium phase contained two plugs for metal analyses and an additional single plug for percent solids analysis for the selenium phase. Each plug sample was placed immediately on dry ice for transport to the laboratory. The scalpel and biopsy punch were discarded after the collection of each series of plug samples from an individual fish. Each fillet plug sample was weighed to the nearest hundredth of a gram in the laboratory on sterile weight boats, using an Ohaus Explorer Model #E01140 Analytical and Precision Balance. For the mercury phase, plug samples were extracted five times from the same fish (yielding five samples, with two plugs per sample) (Fig. 2). For the selenium phase, plug samples were extracted four times from the same fish (yielding four samples, with two plugs per sample) (Fig. 3). An additional single-plug sample was collected from each fish during the selenium phase for percent solids analysis.

In total, the sampling team collected 60 whole fish and used biopsy punches to extract 300 fillet plug samples in the field for the mercury phase of the study. For the selenium phase, they collected 30 whole fish, 120 field-extracted fillet plug samples for selenium analysis, and 120 single plugs for determination of percent solids (i.e., to provide selenium results on the dry-weight basis specified for the selenium water quality criterion).

To prepare the homogenized fillet samples, laboratory personnel removed the scales and the remaining fillet tissue from both sides of each individual fish (keeping the skin on and the “belly flap” or ventral muscle attached). A stainless-steel electric meat grinder was used to prepare each homogenized fillet sample. All fillet tissue from both sides of each whole fish was homogenized, and the entire homogenized volume was used to prepare the fillet tissue sample. Grinding of each sample was repeated until the homogenized tissue had a finely ground texture with uniform color. Analysis of triplicate lipid aliquots was used as a quality control step to ensure that sample aliquots used for analysis were homogeneous, and equipment rinsate analyses were used to confirm that sample preparation batches were free from extraneous contamination. Homogenized fillet samples were stored in a freezer at ≤ -20 °C and shipped to analytical laboratories on dry ice.

### Tissue Analysis

FPES tissue samples were analyzed for mercury and selenium by commercial environmental laboratories rather than research laboratories, which is consistent with USEPA’s approach to its broader program of human health fish tissue studies (Stahl et al. 2009).

### Mercury

Mercury samples were prepared using Procedure I in Appendix to Method 1631 *Total Mercury in Tissue, Sludge, Sediment, and Soil by Acid Digestion and BrCl Oxidation* (USEPA 2001a), and the instrumental analyses were conducted using Method 1631 Revision E, *Mercury in Water by Oxidation, Purge and Trap, and Cold Vapor Atomic Fluorescence Spectrometry (CVAFS)* (USEPA 2002) on a MERX-T automated mercury analyzer (Brooks Rand Instruments). The sample was digested with a combination of nitric and sulfuric acids.

Each fillet plug sample consisted of two tissue plugs collected from the fish specimen and placed in a single vial. The laboratory weighed the mass of plug tissue in each of the five replicate sample vials and analyzed all of the tissue in each of the vials without subsampling. Each homogenized fillet sample consisted of 5 to 10 grams (g) of tissue. The laboratory removed and analyzed approximately 1 g of tissue for each of the five replicate homogenized fillet samples. Both the fillet plug and homogenized fillet tissue sample results were reported in nanograms per gram (ng/g), based on the wet weight of the tissue sample. The laboratory’s method detection limit (MDL) and minimum level (ML, a quantitation limit) for mercury were 0.09 and 0.3 ng/g, respectively, based on a 1-g sample size.

The required quality control samples associated with each batch of 20 fillet tissue samples analyzed for mercury included three bubbler blanks for the CVAFS instrument, three method blanks, a laboratory control sample, and a reference material sample (see Table 2 for the required frequencies and acceptance limits). For the homogenized fillet samples, where more tissue mass was readily available, a matrix spike sample was also analyzed with each batch (as noted in Table 2, the mercury analysis laboratory ran an additional matrix spike sample with each batch).

**Table 2:** QC samples and acceptance criteria for mercury analysis of fish tissue

QC operation	Frequency	Acceptance limit	Summary of QC findings
Bubbler blank	3 blanks run during calibration and with each batch of up to 20 field samples	50 picograms (pg) of mercury	None of the bubbler blanks were above 50 pg, and none of the averages of the 3 blanks in each of the batches were above 25 pg
Method blank	3 method blanks per batch of up to 20 field samples, with analyses interspersed among the samples in the analysis batch	0.4 nanograms (ng) (400 pg) of mercury, *or* Less than one-tenth the concentration of an associated sample	None of the method blanks were above 400 pg. Overall, 60% of the method blanks were non-detects (below 40 pg), and 40% of the method blanks were detects from 40 to 230 pg. The lowest of 600 field sample results was 23 ng/g, or 100 times the highest blank
Laboratory control sample (LCS)	1 LCS prepared per batch of up to 20 field samples, analyzed *once prior to* the analysis of any field samples, *and again at* the end of each analytical batch, spiked at 4.0 ng	70–130% recovery (5.6–10.4 ng/g)	None of the laboratory control sample results fell outside the acceptance limits. Recoveries ranged from 80 to 119% overall
Reference material sample *(laboratory used TORT-3 from the National Research Council of Canada)*	1 per batch of up to 20 field samples	75–125% recovery	None of the reference sample results fell outside the acceptance limits. Recoveries ranged from 88 to 105% overall
Matrix spike sample	1 per batch of up to 20 homogenized fillet tissue samples (**not** required for plug samples due to limited mass) *(laboratory opted to run two matrix spike samples per batch)*	70–130% recovery	None of the matrix spike sample results associated with the homogenized fillet tissue batches fell outside the acceptance limits. Recoveries ranged from 71 to 119% overall

### Selenium

There are no USEPA methods that directly address the analysis of fish tissue for selenium; therefore, commercial laboratories submitted proposals for analytical approaches. Unlike mercury, there is not an abundance of USEPA data on selenium in fish tissue, so one objective was to employ methods with sufficient sensitivity to avoid reporting results as “below the detection limit.” Selenium analyses can also be impacted by significant analytical interferences in samples containing metals and other metalloids; therefore, for the FPES, USEPA elected to use a modification to EPA Method 200.8, *Determination of Trace Elements in Waters and Wastes by Inductively Coupled Plasma-Mass Spectrometry* (USEPA 1994) that employed an inductively coupled plasma (ICP) instrument with a triple quadrupole mass spectrometer detection system to reduce interferences and improve sensitivity (Agilent Model 8800 ICP-QQQ).

Tissue samples were digested using a procedure based on SW-846 Method 3050B, *Acid Digestion of Sediments, Sludges, and Soils* (USEPA 1996) that employs a combination of nitric acid and hydrogen peroxide. As in the mercury phase of the study, each fillet plug sample consisted of two tissue plugs collected from the fish specimen, and the laboratory weighed the mass of plug tissue provided in the sample vial and analyzed all of the tissue in the vial without subsampling. Each homogenized fillet sample aliquot shipped to the analytical laboratory consisted of 20 to 25 g of tissue. The laboratory removed approximately 5 g of homogenized fillet tissue for analysis of each of the four replicate samples and recorded the wet weight of tissue analyzed for each replicate sample. The MDLs for selenium were 43.2 ng/g for 1-g plug samples and 44.9 ng/g for 5-g homogenized fillet samples, and the corresponding ML for both types of fillet samples was 150 ng/g.

The required quality control samples associated with each batch of 20 tissue samples analyzed for selenium included a method blank, a laboratory control sample, and a reference material sample (see Table 3 for the required frequencies and acceptance limits). For the homogenized fillet samples, where more tissue mass was readily available, a matrix spike sample was also analyzed with each batch (as noted in Table 3, the selenium analysis laboratory ran three additional method blanks with each batch).

**Table 3:** QC samples and acceptance criteria for selenium analysis of fish tissue

QC operation	Frequency	Acceptance Limit	Summary of QC findings
Method blank	1 method blank per batch of up to 20 field samples *(Lab opted to run four method blanks per batch)*	Less than the sample size-specific MDL determined for this study *or* less than one-tenth the concentration of an associated sample	None of the method blanks were above the sample size-specific MDLs (43.2 ng/g for nominal 1-g plug samples or 44.9 ng/g for nominal 5-g homogenized fillet samples)
Laboratory control sample (LCS)	1 LCS per batch of up to 20 field samples	80–120% recovery	None of the laboratory control sample results fell outside the acceptance limits. Recoveries ranged from 92 to 108% overall
Reference material sample *(lab used DORM-4 from the National Research Council of Canada)*	1 per batch of up to 20 field samples	75–125% recovery	None of the reference sample results fell outside the acceptance limits. Recoveries ranged from 90 to 110% overall
Matrix spike sample	1 per every 20 homogenized fillet tissue samples (**not** required for plug samples)	70–130% recovery	None of the matrix spike sample results associated with the homogenized fillet tissue batches fell outside the acceptance limits. Recoveries ranged from 72 to 106% overall

Both fillet plug and homogenized fillet tissue sample results were reported in ng/g, based on the wet weight of the tissue sample. However, because the USEPA selenium fish fillet tissue-based water quality criterion is expressed in terms of the dry-weight selenium concentration, percent solids were also determined for each type of tissue sample using a separate sample aliquot (i.e., a single plug from each fish and a 1-g aliquot of homogenized fish tissue). The solids content was determined by drying each of these aliquots to a constant weight at 103–105 ºC, using Standard Method 2540G (APHA 2005), and the percent solids results were used to calculate the selenium results in a dry-weight format (wet-weight result divided by % solids).

### Data Analysis

The FPES was designed so that the results would be amenable to routine statistical analyses. The study design included:

- fillet plug samples and homogenized fillet samples collected from the same fish specimens,
- replicate measurements from each fish specimen for each sample type (five replicates for mercury analysis and four for selenium analysis), and
- replicate specimens of each fish species (ten replicates for mercury analysis and five for selenium analysis).

Mercury and selenium results were evaluated separately using the same statistical approach. The null hypothesis (H_0_) for the mercury phase is that the two sampling alternatives, i.e., fillet plugs and homogenized fillets (or the “treatments” in statistical parlance), yield equivalent mean concentrations of mercury for any given fish sample. The H_0_ for the selenium phase is that the two sampling alternatives yield equivalent mean wet-weight concentrations and mean dry-weight concentrations of selenium for any given sample. Summary statistics were generated and tabulated for each set of data (i.e., mercury wet-weight concentrations; selenium wet-weight concentrations and dry-weight concentrations). The data summaries were used to identify any need for data transformations (such as the log transformation) and/or identify unexpected patterns in the data. Measures of central tendency including mean and median were calculated, as were measures of variability including standard deviation, variance, and relative standard deviation (RSD), and additional percentiles of the distribution (e.g., minimum and maximum, first and third quartiles). Summary statistics were calculated separately for each tissue collection method (i.e., field plug and homogenized fillet) and for each combination of tissue collection method, sampling location, and species.

Statistical analyses were performed using multi-factor analysis of variance (ANOVA) models for each of the four evaluated measurements (mercury in wet weight, selenium in wet weight, selenium in dry weight, and percent solids), with sample type (i.e., fillet plugs and homogenized fillets) as the primary factor of interest. Waterbody/species was included as a single factor because the design precluded these from being evaluated separately. Additionally, a sample type x waterbody/species interaction term was initially included in each model and removed if not statistically significant. All four outcome measurements were log-transformed prior to inclusion in the model. Additionally, to mitigate concerns regarding the lack of independence among measurements within a specimen, the data were log-transformed and then aggregated to the specimen level prior to fitting the ANOVA models.

Evaluations of statistical significance of sample type effects were performed at the 95% confidence level. Relevant ANOVA assumptions, including normality of model residuals and constant variability with concentration and across sample types and waterbodies, were evaluated to confirm that any conclusions drawn from the model results were statistically valid.

## Analytical and Statistical Results

### Mercury

Mercury concentration data for all samples are provided in Supplementary Information Table SI1. Results and descriptive statistics for the 600 mercury sample results are summarized in Table 4 across all of the sampling locations and by sample type (fillet plug vs. homogenized fillet). Table 5 summarizes the mercury results within each of the six sampling locations by sample type (fillet plug vs. homogenized fillet). Mercury concentrations in the samples varied widely across waterbodies and species, with higher mean concentrations for Lake Erie Walleye and Potomac River Largemouth Bass, and lower mean concentrations for Anacostia River Blue Catfish. The results in this study range from 23 to 649 ng/g, which is well within the ranges of mercury concentrations noted for USEPA’s probabilistic fish tissue contamination studies of the nation’s inland lakes, rivers, and Great Lakes conducted since 2000. For example, mercury concentrations in predator fish species collected for USEPA’s National Lake Fish Tissue Study ranged from 23 ng/g to a maximum of 6,605 ng/g (Stahl et al. 2009), those collected for the 2008–2009 National Rivers and Streams Assessment ranged from 21 to 1,418 ng/g (USEPA 2016b), and those collected from the Great Lakes for the 2010 National Coastal Condition Assessment ranged from 23 to 956 ng/g (USEPA 2015). The data demonstrate that the FPES fish samples represent a range of mercury concentrations that is typical of what has been found in other USEPA fish tissue studies.

**Table 4:** Descriptive statistics for mercury results across all sampling locations by sample type

Sample type	Count	Mercury concentration (ng/g)									RSD (%)
		Min	10th Percentile	25th Percentile	Median	Mean	75th Percentile	90th Percentile	Max	SD	
FP	300	44.2	82.4	101.0	121.0	155.2	171.3	271.0	649.0	101.6	65.5
HF	300	23.0	91.0	105.0	143.0	161.4	185.3	262.0	556.0	86.5	53.6

FP, fillet plug sample; HF, homogenized fillet sample; ng/g, nanograms per gram; SD, standard deviation; RSD, relative standard deviation

**Table 5:** Descriptive statistics for mercury results by sampling location, species, and sample type

Site	Species	Sample type	Count	Mercury concentration (ng/g)							RSD (%)
				Min	25th Percentile	Median	Mean	75th Percentile	Max	SD	
Anacostia River	Blue Catfish	FP	50	44.2	68.5	88.2	107.5	126.0	308.0	61.8	57.5
		HF	50	23.0	69.6	86.5	106.9	132.0	284.0	61.3	57.4
Potomac River	Largemouth Bass	FP	50	74.0	97.4	109.0	177.4	186.0	526.0	139.0	78.4
		HF	50	86.8	102.0	118.0	176.8	213.0	475.0	115.7	65.4
St. Lawrence River	Smallmouth Bass	FP	50	99.6	160.0	185.5	195.6	236.0	322.0	59.8	30.5
		HF	50	105.0	173.0	190.0	199.7	241.0	317.0	54.6	27.3
Lake Michigan	Lake Trout	FP	50	81.0	92.1	131.0	129.8	157.0	211.0	39.2	30.2
		HF	50	95.2	104.0	155.0	148.1	183.0	222.0	42.1	28.4
Lake Erie	Walleye	FP	50	86.7	103.0	124.0	198.2	213.0	649.0	162.9	82.2
		HF	50	87.5	108.0	130.0	189.3	207.0	556.0	133.2	70.3
Lake Ontario	Chinook Salmon	FP	50	106.0	115.0	119.5	122.4	130.0	143.0	10.4	8.5
		HF	50	119.0	139.0	149.0	147.9	155.0	172.0	12.4	8.4

FP, fillet plug sample; HF, homogenized fillet sample; ng/g, nanograms per gram; SD, standard deviation; RSD, relative standard deviation

**Fig. 4:**
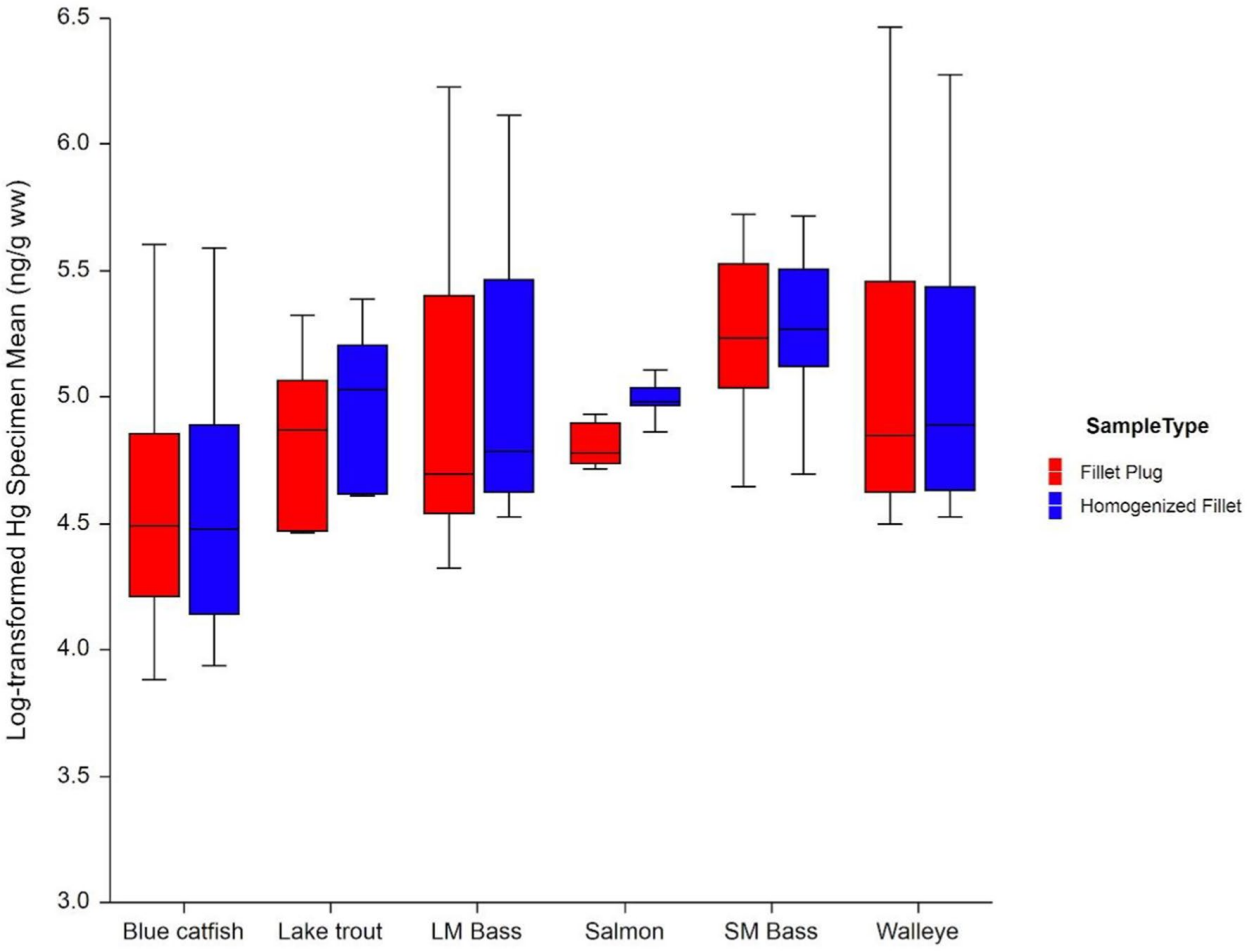
Mean natural log-transformed mercury wet-weight results by species and sample type

Mean mercury concentrations in the fillet plugs (FP) and homogenized fillets (HF) varied by a smaller magnitude within each waterbody and species, with the largest differences observed for Lake Michigan Lake Trout and Lake Ontario Chinook Salmon (with lower mean concentrations for FP samples) (Table 5 and Fig. 4). The variability in concentrations also differed between species. Standard deviations and RSDs were highest for Lake Erie Walleye and Potomac River Largemouth Bass. These sites/species had higher mean mercury concentrations, such that the higher standard deviations could be a function of the expected positively skewed distributions. The higher RSDs and consistent variability measures across the two sample types could also indicate that the variability in concentration across the individual fish specimens may be greater in some species than in others. The smallest variability measures were observed for the Lake Ontario Chinook Salmon samples; for both sample types, the RSD was approximately 8.5%. The concentrations from this site/species were not notably lower than they were for the other sites/species, indicating that the variability in concentration between individual fish was much lower for this site/species.

**Fig. 5:**
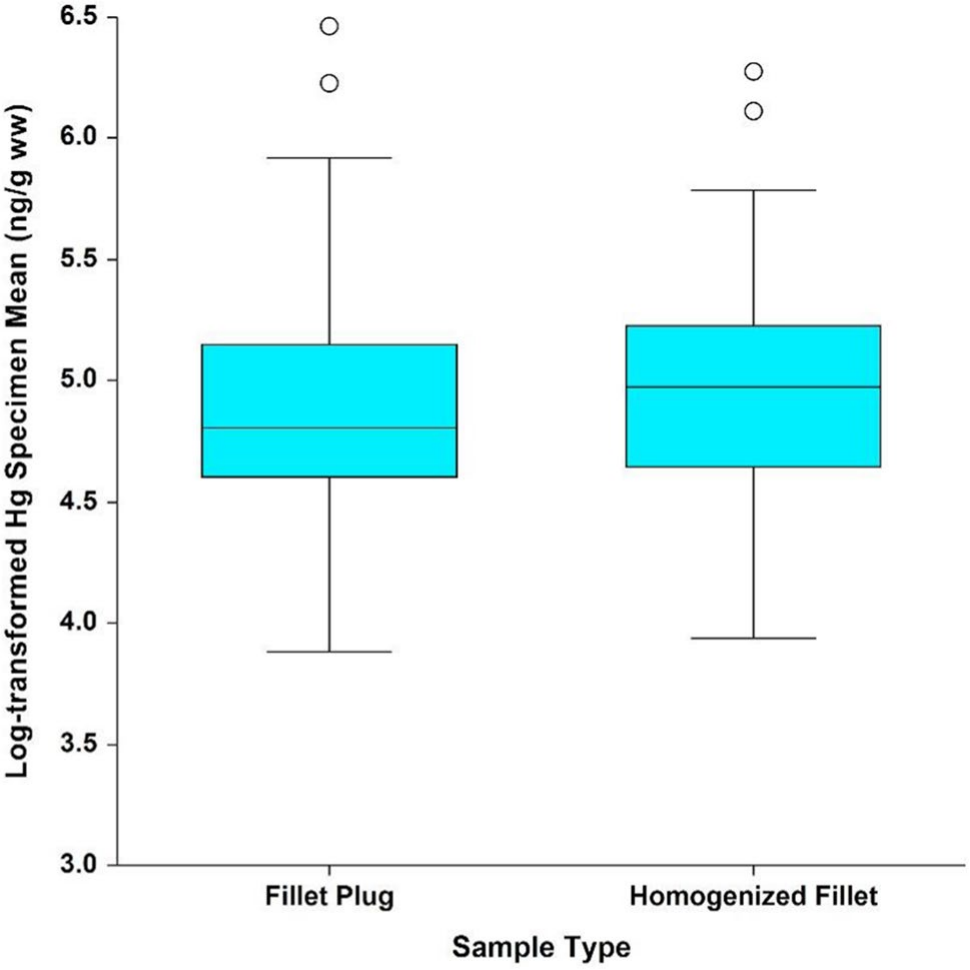
Mean natural log-transformed mercury wet-weight results by sample type

The ANOVA model was initially fit by including a waterbody x sample type interaction term. This term was not statistically significant at an alpha of 0.05 (*p* = 0.973); therefore, conclusions regarding sample type can be made irrespective of waterbody and species, and a single overall comparison can be made between the two sample types. After removing the interaction term, the effect of sample type was also found to not be statistically significant (*p* = 0.405) (Fig. 5).

When evaluating the statistical assumptions regarding the fitted model, the natural log-transformed results deviate from a normal distribution slightly (Shapiro–Wilk W = 0.921, *p* < 0.0001) (Shapiro and Wilk 1965); however, the departure was not to a large enough degree to impact the results. Model variability did not vary with the measured concentrations (i.e., no pattern was observed between the model residuals and measured concentrations), and Levene’s test indicated no significant difference in model variability between sample types (*p* = 0.795) (Levene et al. 1960). As discussed above, variability between measured concentrations was not consistent across waterbodies (Levene’s test *p* < 0.0001 when comparing model variability across waterbodies), due to the smaller variation between Lake Ontario specimens. However, this would have little impact on the conclusions, because any low bias in the calculated model error (i.e., the mean squared error) due to the Lake Ontario data would have made statistical significance of sample type more likely, rather than less likely.

### Selenium Results

**Fig. 6:**
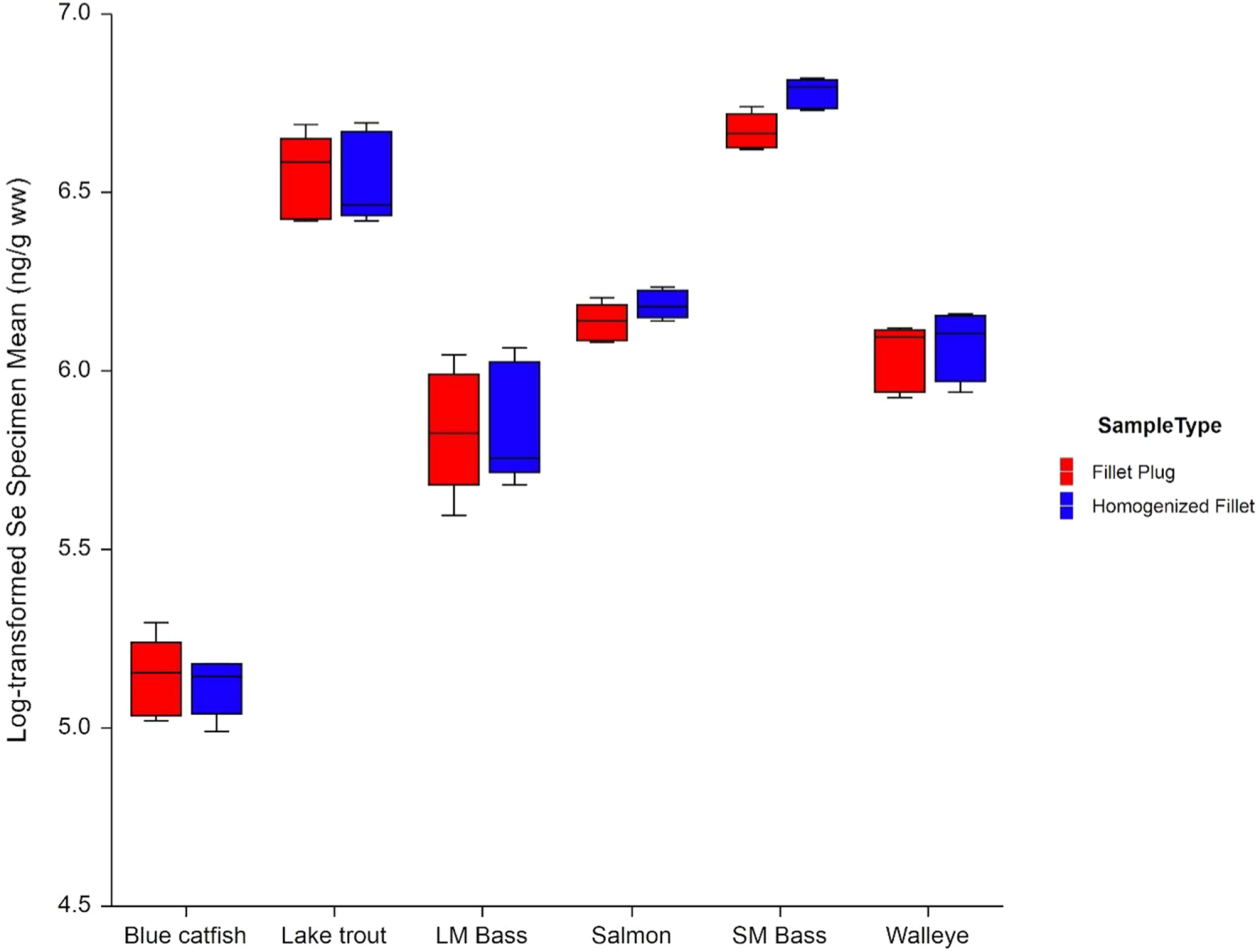
Mean natural log-transformed selenium wet-weight results by species and sample type

Selenium concentration data for all samples are provided in Supplementary Information Table SI2. Descriptive statistics for the 240 selenium sample results are detailed in Table 6 across all of the sampling locations by sample type (field plug vs. homogenized fillet), for both wet-weight and dry-weight selenium results. Results within each of the six sampling locations are summarized in Table 7 by sample type. As with the mercury results, selenium concentrations in the FPES samples varied widely across waterbodies and sites, with higher mean concentrations for Lake Michigan Lake Trout and St. Lawrence River Smallmouth Bass, and lower mean concentrations for Anacostia River Blue Catfish (Table 7) (Fig. 6). Wet-weight selenium results ranged from 139 to 932 ng/g. The variability in the wet-weight selenium results, evidenced by the RSD values in Table 7, was less than the variability in the mercury results. The largest site-specific RSD for wet-weight selenium was about 16% versus over 80% for mercury.

**Table 6:** Descriptive statistics for selenium results across all sampling locations by sample type

Measure	Sample type	Count	Selenium concentration (ng/g)									RSD (%)
			Min	10th Percentile	25th Percentile	Median	Mean	75th Percentile	90th Percentile	Max	SD	
Se (ng/g ww)	FP	120	139.0	174.5	340.0	447.0	482.2	718.5	794.0	848.0	214.4	44.5
	HF	120	145.0	174.5	318.0	466.5	502.0	661.5	877.5	932.0	236.7	47.2
Se (ng/g dw)	FP	120	711.0	859.0	1544.5	1762.0	1922.0	2101.0	3402.0	3814.0	810.1	42.2
	HF	120	750.0	874.5	1542.0	1781.5	1996.0	2090.0	3829.5	4084.0	936.2	46.9
Total Solids (%)	FP	120	17.0	19.9	21.0	22.6	25.0	27.5	34.0	45.8	5.8	23.1
	HF	120	17.8	19.7	20.7	22.8	25.1	27.5	36.2	41.0	6.0	24.0

FP, fillet plug sample; HF, homogenized fillet sample; ng/g, nanograms per gram; ww, wet-weight basis; dw, dry-weight basis; SD, standard deviation; RSD, relative standard deviation

**Table 7:** Descriptive statistics for selenium and solids results by sampling location, species, and sample type

Measure	Site	Species	Sample type	Count	Selenium concentration (ng/g)							RSD (%)
					Min	25th Percentile	Median	Mean	75th Percentile	Max	SD	
Se (ng/g ww)	Anacostia River	Blue Catfish	FP	20	139.0	154.5	172.5	172.0	179.0	234.0	21.6	12.5
			HF	20	145.0	158.5	170.0	167.2	177.5	182.0	12.6	7.5
	Potomac River	Largemouth Bass	FP	20	264.0	314.5	340.0	344.8	377.0	434.0	53.7	15.6
			HF	20	290.0	309.5	318.0	350.3	401.0	437.0	55.6	15.9
	St. Lawrence River	Smallmouth Bass	FP	20	736.0	759.5	783.5	789.9	815.5	848.0	37.1	4.7
			HF	20	829.0	846.5	889.0	879.7	906.0	932.0	33.5	3.8
	Lake Michigan	Lake Trout	FP	20	599.0	621.0	718.5	701.3	755.0	823.0	76.1	10.8
			HF	20	578.0	624.0	661.5	694.8	775.0	867.0	89.1	12.8
	Lake Erie	Walleye	FP	20	360.0	387.0	437.0	422.3	450.5	469.0	36.7	8.7
			HF	20	367.0	395.5	437.5	433.9	465.5	491.0	39.9	9.2
	Lake Ontario	Chinook Salmon	FP	20	428.0	442.5	461.0	463.0	479.5	504.0	23.4	5.0
			HF	20	452.0	466.5	490.0	486.1	502.5	534.0	22.4	4.6
Se (ng/g dw)	Anacostia River	Blue Catfish	FP	20	711.0	765.5	834.0	848.0	899.0	1158.0	106.9	12.6
			HF	20	750.0	841.5	868.0	854.0	884.0	925.0	48.4	5.7
	Potomac River	Largemouth Bass	FP	20	1389.0	1565.0	1658.5	1678.0	1811.0	1978.0	190.2	11.3
			HF	20	1452.0	1512.5	1573.5	1670.0	1858.5	1993.0	183.1	11.0
	St. Lawrence River	Smallmouth Bass	FP	20	3211.0	3355.0	3441.0	3458.0	3539.0	3814.0	147.9	4.3
			HF	20	3647.0	3798.0	3842.5	3869.0	3956.0	4084.0	112.0	2.9
	Lake Michigan	Lake Trout	FP	20	1619.0	1724.0	1873.5	2014.0	2293.0	2669.0	359.9	17.9
			HF	20	1495.0	1631.5	1749.0	1917.0	2310.0	2611.0	364.2	19.0
	Lake Erie	Walleye	FP	20	1549.0	1718.0	1896.5	1888.0	2040.5	2176.0	193.6	10.3
			HF	20	1643.0	1792.0	1912.0	1934.0	2062.5	2299.0	178.8	9.2
	Lake Ontario	Chinook Salmon	FP	20	1427.0	1544.5	1630.5	1645.0	1761.0	1925.0	146.8	8.9
			HF	20	1432.0	1698.0	1765.5	1734.0	1818.0	1922.0	131.9	7.6
Total Solids (%)	Anacostia River	Blue Catfish	FP	20	17.0	19.8	20.5	20.3	21.4	23.9	1.7	8.3
			HF	20	17.8	18.8	19.7	19.6	20.5	20.9	1.0	5.1
	Potomac River	Largemouth Bass	FP	20	18.7	19.8	20.5	20.4	20.9	22.0	0.9	4.5
			HF	20	19.4	20.0	20.4	20.9	21.4	23.6	1.3	6.2
	St. Lawrence River	Smallmouth Bass	FP	20	21.2	22.5	22.8	22.8	23.3	24.5	0.8	3.3
			HF	20	21.6	22.3	22.9	22.7	23.2	24.4	0.7	3.1
	Lake Michigan	Lake Trout	FP	20	29.8	32.6	35.4	35.3	37.3	45.8	3.9	10.9
			HF	20	32.9	34.7	37.3	36.6	38.1	41.0	2.5	6.7
	Lake Erie	Walleye	FP	20	21.1	21.8	22.1	22.4	22.7	24.6	0.9	3.8
			HF	20	21.4	22.1	22.5	22.4	22.9	23.4	0.6	2.5
	Lake Ontario	Chinook Salmon	FP	20	24.8	26.9	27.5	28.3	29.7	33.3	2.7	9.4
			HF	20	26.1	26.9	27.5	28.1	28.8	32.3	1.8	6.5

FP, fillet plug sample; HF, homogenized fillet sample; ng/g, nanograms per gram; ww, wet-weight basis; dw, dry-weight basis; SD, standard deviation; RSD, relative standard deviation

### Selenium Concentration (Wet-Weight Basis)

**Fig. 7:**
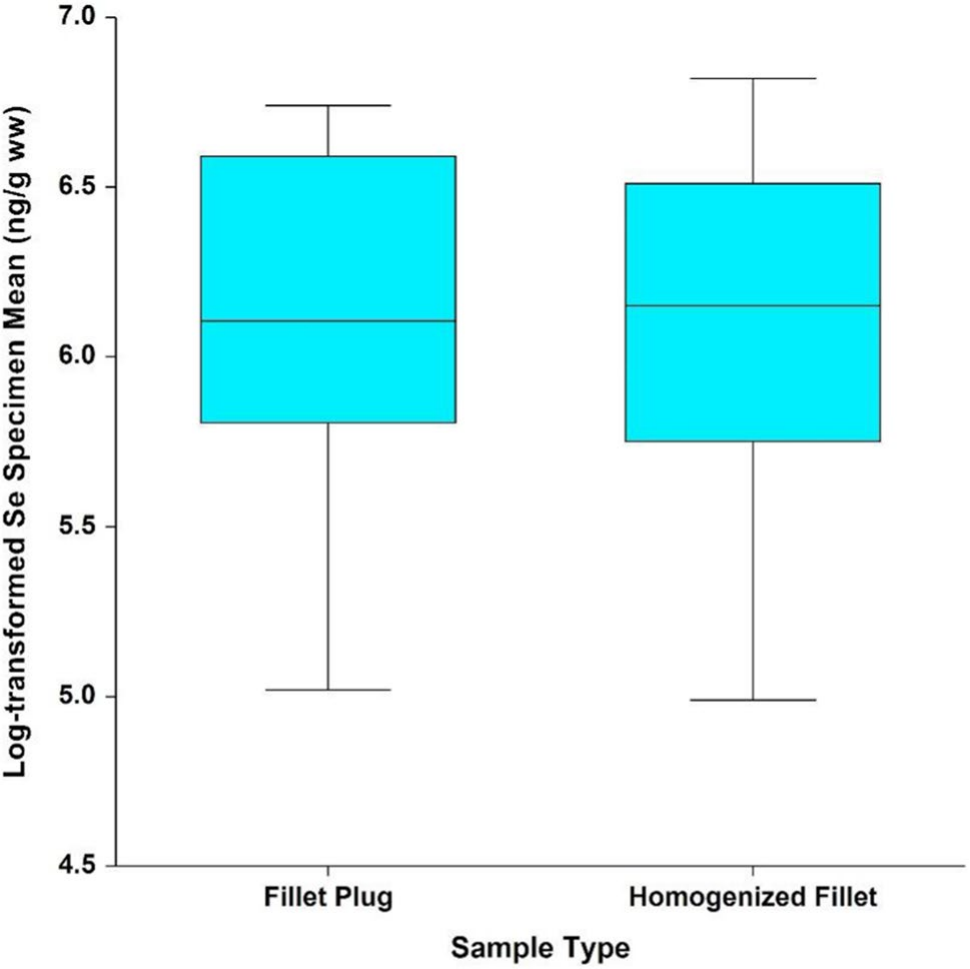
Mean natural log-transformed selenium wet-weight results by sample type

Similar to the mercury data, no statistically significant interaction was observed between waterbody and sample type for selenium wet-weight results (*p* = 0.767), indicating that conclusions regarding sample type can be made irrespective of waterbody and species, and a single overall comparison can be made between the two sample types. After removing the interaction term, the effect of sample type was not statistically significant (*p* = 0.304), as shown in Fig. 7. When evaluating the statistical assumptions regarding the fitted model, the log-transformed results do not deviate from a normal distribution, based on the Shapiro–Wilk test (*p* = 0.645). Model variability did not vary with the measured concentrations (i.e., no pattern was observed between the model residuals and measured concentrations), and Levene’s test indicated no significant difference in model variability between sample types (*p* = 0.819). The model variability was not consistent across waterbodies, due to the smaller variation between Lake Ontario and St. Lawrence River specimens, compared to those collected from the other sites (Levene’s test *p* < 0.0001 when comparing model variability across waterbodies). This was consistent with what was observed for mercury.

### Selenium Concentration (Dry-Weight Basis)

**Fig. 8:**
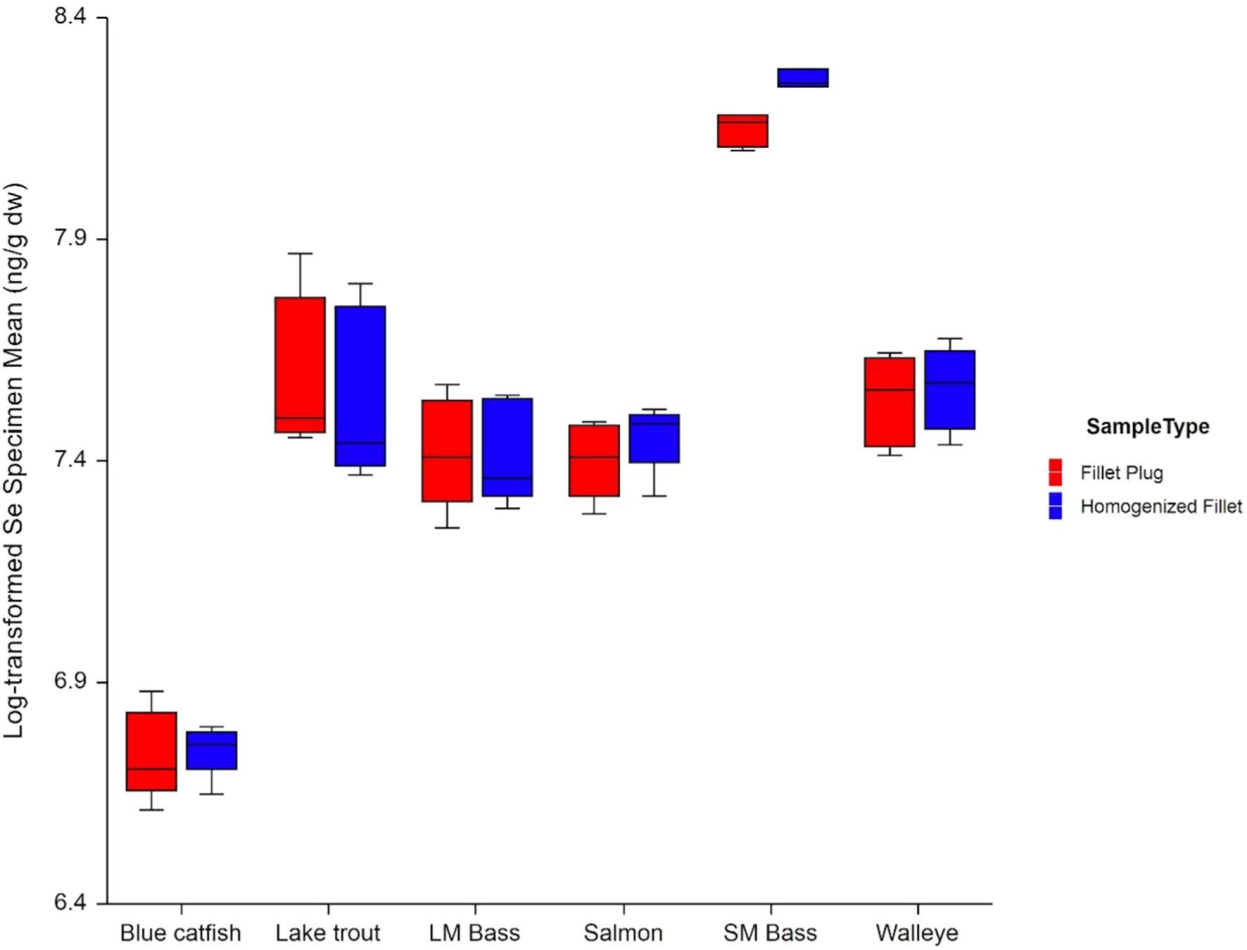
Mean natural log-transformed selenium dry-weight results by species and sample type

**Fig. 9:**
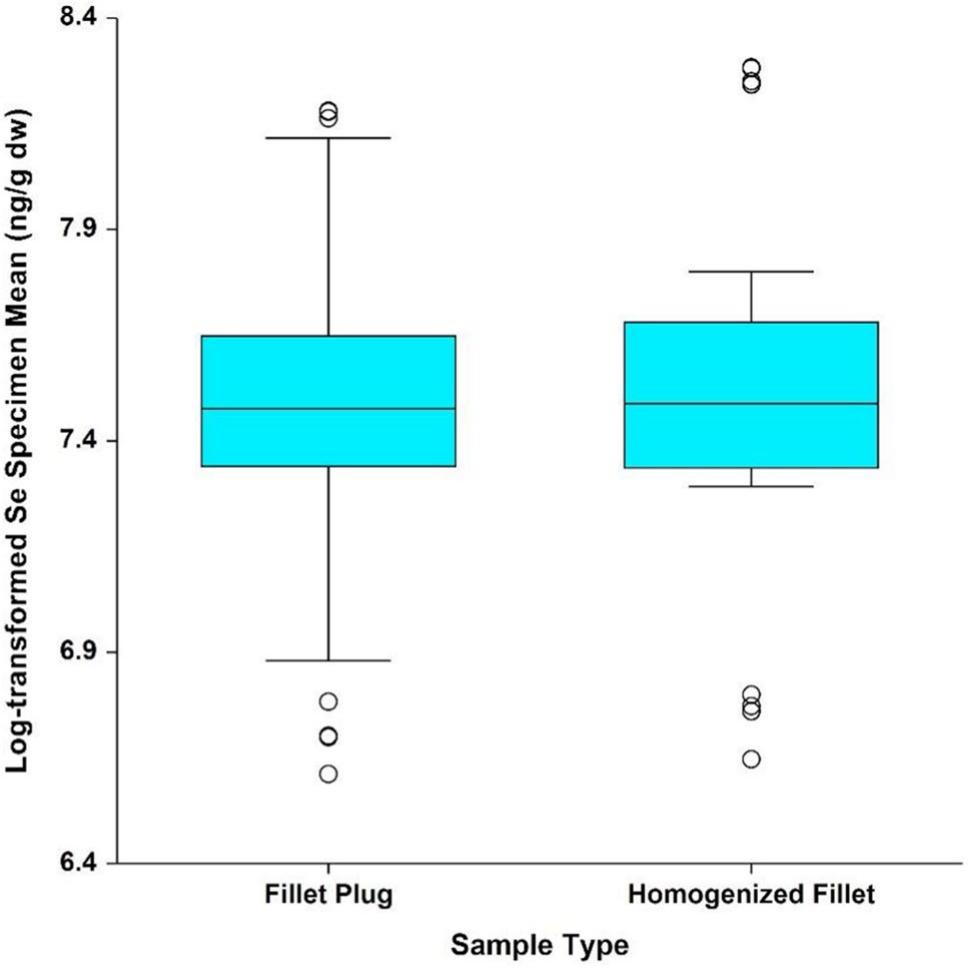
Mean natural log-transformed selenium dry-weight results by sample type

Analysis results for dry-weight selenium concentrations were consistent with those for mercury and wet-weight selenium concentrations (Table 7 and Fig. 8). There was no statistically significant waterbody x sample type interaction (*p* = 0.674), and after removal of the interaction term from the model, the effect of sample type was not statistically significant (*p* = 0.379). Based on this evaluation, the mean fillet plug dry-weight selenium concentration did not differ from the mean homogenized fillet dry-weight selenium concentration (Fig. 9). The evaluation of statistical assumptions was consistent with that of the wet-weight concentrations. The log-transformed results did not deviate from a normal distribution, based on the Shapiro–Wilk test (*p* = 0.551). The model variability did not differ significantly between sample types (Levene’s test *p* = 0.959), but was not consistent across waterbodies (Levene’s test *p* = 0.0002), due to the smaller variation between Lake Ontario and St. Lawrence River specimens; however, as with the wet-weight selenium results, this should not impact the conclusions.

### Total Solids

**Fig. 10:**
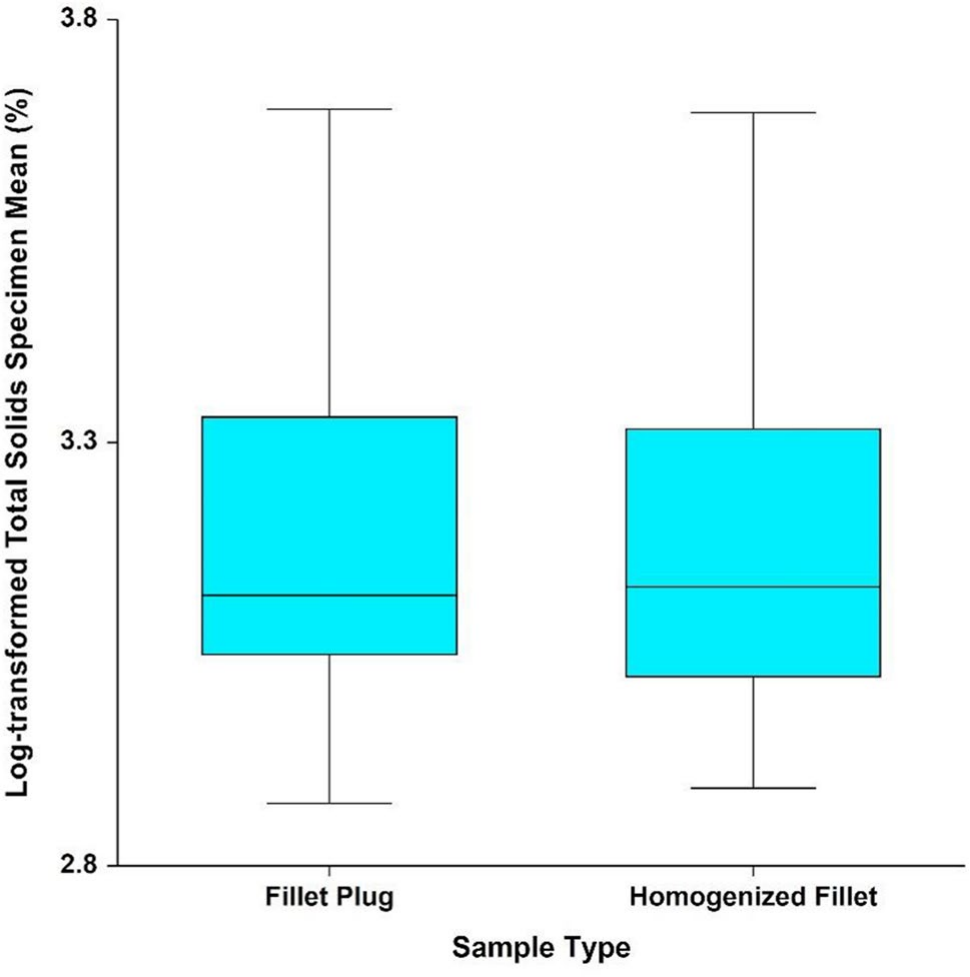
Mean natural log-transformed total solids results (%)

Analytical results for total solids were consistent with those from all other measurements. The waterbody x sample type interaction was not statistically significant (*p* = 0.820). Therefore, conclusions regarding sample type can be made irrespective of waterbody and species and a single overall comparison can be made between the two sample types. After removing the interaction term, the effect of sample type was not statistically significant (*p* = 0.863), as shown in Fig. 10. As with the selenium results, the log-transformed results do not deviate from a normal distribution (*p* = 0.129), model variability does not vary between sample types (Levene’s test *p* = 0.677), and differences in model variability across sites (Levene’s test *p* = 0.022) would not impact study conclusions.

### Analytical Quality Control (QC) Results

Analytical results for each sample in each sample batch were reviewed against the QC requirements for the analytical methods and the study (Tables 2 and 3). The QC operations included method blanks, a laboratory control sample, and a reference material sample. For the homogenized fillet samples, a matrix spike sample was also analyzed with each batch of tissue samples. In addition to reviewing all of the QC results, the final results for at least two samples in every batch were traced from the raw instrument data to the reported result, confirming each of the laboratory’s calculations. As summarized in Tables 2 and 3, no QC problems occurred during either the mercury or selenium analyses. All method blanks were free of contaminants at the detection limits, and no errors or analytical inconsistencies were identified. Tables 2 and 3 also summarize the observed results for each QC operation.

## Discussion

### Mercury

Based on the statistical analyses of 600 results for mercury, there was no statistically significant difference between the fillet plug samples and the homogenized fillet samples. This indicates that mercury concentrations in fish can be monitored in fish tissue by collecting and analyzing plug samples without biasing the results in comparison with analyzing homogenized fillet samples. The lack of a statistically significant difference between the two tissue sample collection techniques holds true across the six fish species studied and across the range of concentrations (23 to 649 ng/g wet weight) found in the rivers and lakes that were sampled.

One important use of fish tissue monitoring results is to identify areas where mercury concentrations exceed USEPA’s fish tissue-based water quality criterion for methyl mercury of 300 ng/g (USEPA 2001b). Therefore, although the analyses of the entire set of 600 results indicated no statistically significant differences, all 300 pairs of observations of fillet plug results and homogenized fillet results were also evaluated relative to the 300 ng/g benchmark value. In total, 553 of the 600 mercury results were less than or equal to 300 ng/g, and 47 results were greater than 300 ng/g.

Closer examination of the data set identified five instances where the use of plug sampling could have resulted in a different decision (i.e., than fillet sampling) regarding safe consumption of the fish. One set of paired results was for the sixth Blue Catfish specimen from the Anacostia River. The fillet plug result in the fourth replicate plug from that one fish specimen was 308 ng/g, and the homogenized fillet result was 284 ng/g from that same fish. Using the plug sample result would lead to the conclusion that the sample exceeded the human health benchmark value, whereas the homogenized fillet sample result would lead to the opposite conclusion. All of the other plug sample and homogenized fillet results for that specimen were below 300 ng/g, indicating that the individual fish did not actually exceed the benchmark. The mean mercury concentration in all five plug samples from that Blue Catfish specimen was 272.2 ng/g and the mean of the homogenized fillet samples was 267.4 ng/g, so it can be concluded that mercury concentrations did not exceed the benchmark value based on results from either sample type.

The other four sets of paired results that spanned the 300 ng/g mercury benchmark were all from the fourth Smallmouth Bass specimen from the St. Lawrence River. (The fifth pair of results from that specimen both were slightly over 300 ng/g.) The mean mercury concentration in those four plug samples from that Smallmouth Bass specimen was 304.25 ng/g, and the mean of the four homogenized fillet samples was 304.0 ng/g. Adding in the results from the fifth pair of results makes the mean plug sample concentration 305.4 ng/g and the mean homogenized fillet sample concentration 303.8 ng/g. Therefore, when viewed in aggregate, the mercury concentrations do exceed the mercury benchmark value in this specimen.

Of those four pairs of results, the fillet plug results in two pairs were below the 300 ng/g benchmark and the homogenized fillet results were above it, while in the other two pairs, the opposite situation occurred. Thus, it appears that the observed differences could be a random effect of the arbitrary numbering system of the plug locations on a specimen.

The absolute values of the differences between all four of the pairs of samples that split the benchmark value range from 24 to 35 ng/g, which are roughly 10% of the reported concentrations. Such differences are generally within the expected precision of the analytical method for mercury, further suggesting that the differences between the paired results may simply be random variations.

In an example scenario where only one fish is collected from a site for study and only a plug sample is taken for mercury analysis, the FPES data suggest that the risk of making the “wrong” decision relative to the 300 ng/g benchmark value would be 5 in 300, or 1.67%. While that risk is small, it could be reduced by collecting at least one more specimen at the site and averaging the plug sample results. Alternatively, even collecting a second plug sample from the same fish specimen and averaging the results may reduce the risk (although it could impact fish health or survival). For example, in the single pair of conflicting results in the Blue Catfish specimen, even averaging the second highest plug result of 276 ng/g with the highest result of 308 ng/g would yield 292 ng/g, which is below the benchmark value.

Considering all five fillet plug results from that one Smallmouth Bass specimen, there are ten possible combinations of any two plug samples. If one were to average the results within each of those 10 random pairs, seven average mercury concentrations would be above 300 ng/g and three averages would be below 300 ng/g. That suggests that the 1.67% decision error risk when using one plug sample cited above might be reduced to 0.5% (e.g., 1.67% × 0.30 = 0.501%) if two plug samples per specimen are used.

### Selenium

Statistical analyses of 240 results for selenium, in both wet-weight and dry-weight concentrations, also showed that there were no statistically significant differences between the fillet plug samples and the homogenized fillet samples using either reporting convention. As with the mercury results, the lack of a statistically significant difference between the two sample collection techniques holds true across the six fish species studied, and across the range of concentrations (139 to 932 ng/g wet weight, and 711 to 4,084 ng/g dry weight) found in the rivers and lakes that were sampled.

One study objective was to test the applicability of fish fillet plug sampling and analysis for conducting surveillance monitoring associated with USEPA’s recommended freshwater selenium ambient chronic water quality criterion for fish fillet tissue of 11,300 ng/g (dry weight) (USEPA 2016a). None of the FPES samples contained selenium at or above that criterion, or even near that benchmark. Therefore, it was not possible to examine any paired selenium results near the selenium fish fillet tissue-based water quality criterion. However, these data indicate that either of the two sample collection techniques can be used to monitor selenium concentrations in fish fillet tissue relative to the water quality criterion, with some caveats for plug sampling. Collecting fillet plug samples in the field can accomplish the goal if the analytical laboratory implements a sufficiently sensitive analytical procedure. The laboratory should employ sample preparation and analysis techniques that can measure selenium using samples weighing no more than 1 g (wet weight). The sensitivity of the process (expressed as the quantitation limit) should be well below the selenium water quality criterion (for the fillet tissue fraction) and should include consideration of the range of total solids contents of the fish under study, which may range from 17 to 40% solids based on this study. Whole fish sampling for homogenized fillet sampling and analysis can accomplish the goal as well, but the ability to analyze a sample aliquot larger than 1 to 2 g means that the final determinative method need not be as sensitive as the method required for plug sample analysis.

Because reporting dry-weight results for selenium (e.g., for monitoring associated with the USEPA selenium water quality criterion) involves separate measurements of selenium and solids, the end result of the analyses includes variability introduced during both analyses. While analytical methods for selenium include a range of QC operations and acceptance criteria, procedures for determining the solids content of tissue samples generally include fewer QC operations. For example, there are no standards or reference materials available for solids analyses of tissue samples. As noted earlier, an additional sample plug is needed to conduct solids analysis, whereas the size of homogenized fillet samples can be increased in the containers to provide sufficient mass for both selenium analysis and solids analysis.

One analytical alternative could be to lyophilize (freeze dry) each sample before digesting it for the selenium analysis. The FPES did not consider using that approach for several reasons, including the fact that there are no readily available reference methods for freeze-drying tissues. If freeze-drying were to be used, it would be critical to record the initial wet weight of the sample aliquot and the final weight after drying and before calculating the solids content of the sample so that the results were available for comparison to other data sets for the same species in the literature.

## Conclusions

Both the mercury and selenium phases of this study showed that there were no statistically significant differences between fillet plug and homogenized fillet results; however, for selenium, the plug sampling alternative must employ a sufficiently sensitive analytical method and should consider total solids. Baker et al. (2004) noted that the reliability of results from small fish tissue samples collected with biopsy tools depends on the analytical methodology and the tissue sample weight, and cautioned that loss of moisture in small-volume samples in the field or during freezer storage is an important potential source of variation. Those potential sources of variation, i.e., field handling and prolonged freezer storage of fillet plug samples, warrant further investigation. The FPES conclusions apply to mercury and selenium only and cannot be extrapolated to other chemical contaminants. The purpose of this study was to advance our understanding of mercury and selenium fish tissue sampling alternatives for monitoring at the national or regional scales and therefore focuses on the community level. Local researchers may be interested in comparisons of fillet plugs (FP) versus homogenized fillets (HF) at the specimen level for studies on a smaller scale. Scatterplot comparisons of the FPES FP versus HF concentration data are provided in Supplementary Information Figure SI1 for mercury and Figure SI2 for selenium. The mercury and selenium plots show some variability; however, there is consistent agreement across species along the 1:1 line, the distance of the specimen mean comparison points from the 1:1 line is generally small, and that variability does not result in statistically significant differences at the community level. These study findings are consistent with those from the site-specific study of Baker et al. (2004) and Canadian research by Knight et al. (2019). Baker et al. (2004) found some statistical differences in mercury concentrations between biopsy samples and fillets, but concluded that the magnitude of the differences was small and within the range expected in multiple-sample and multiple-laboratory comparisons. Knight et al. (2019) concluded that mercury concentrations in skinless biopsy plug samples were statistically equivalent to those in homogenized skinless fillets and postulated that inclusion of skin should not have any major impacts on their study findings. Other researchers reached similar conclusions to the FPES in site-specific or laboratory studies of mercury (Ackerson et al. 2014; Peterson et al. 2005) and selenium (Waddell and May 1995) in freshwater fish plugs versus fillets.

The target species in the FPES are representative of freshwater sportfish species commonly caught and consumed in the USA; therefore, these findings can be extrapolated to similar freshwater species, but they may not apply to all fish species (e.g., estuarine or marine species were not tested). Also, studies designed to analyze contaminants in whole fish (rather than fillet tissue) would not be expected to yield similar plug sample comparison conclusions to those described here. In this study, plug samples were collected from a specifically delineated area on each fish, i.e., in the dorsal muscle between the lateral line and the dorsal fin. Collecting plug samples from other areas of the fish musculature may not yield results similar to the FPES and therefore may not represent a suitable alternative for fillet analysis.

Both fish plug and homogenized fillet sampling alternatives have inherent advantages and disadvantages for monitoring applications (Table 8). A choice between sampling alternatives ultimately depends on study objectives, target chemicals, and tissue volumes needed for analysis. Homogenized fillet sampling provides sufficient mass for the analysis of multiple contaminants but requires fish to be killed for analysis, whereas plug sampling may allow fish survival following collection but only provides adequate tissue mass for a single analyte (e.g., mercury or selenium). Morizot et al. (1990) listed six points to consider when assessing the applicability of a minimally invasive procedure (like fillet plug sampling) for fish field monitoring, stating that it should have minimal impact on fish survival, have minimal impact on fish health and fitness, provide sufficient tissue mass, require minimal post-sampling treatment of fish and easy storage under field/laboratory conditions, be applicable for most fish taxa, and require minimal training of field personnel. After testing fish field plug methods for monitoring mercury in 31 Smallmouth Bass, Ackerson et al. (2014) concluded that plug methods satisfy all of the Morizot et al. (1990) applicability points; however, other authors note that sublethal effects (e.g., infection) and fish mortality associated with widespread monitoring using plug methods under ambient conditions are largely unknown (Waddell and May 1995; Baker et al. 2004; Schmidt et al. 2016). Fillet plug muscle wound healing in Rainbow Trout (*Oncorhynchus mykiss*) was documented by Schmidt et al. (2016) to progress slowly in the laboratory, and progression was partially ascribed to water temperature (the inflammation phase lasted for 14 days or more, the dermis was still thickened and disorganized around the wound after 100 days, but by 100 days the epidermis had regenerated). Other fish fillet plug researchers have characterized it as a non-lethal sampling technique, with or without supporting survival data (Ackerson et al. 2014; Knight et al. 2019). While the FPES demonstrated that there are no statistically significant differences in the mercury or selenium results from fillet plug samples compared to homogenized fillets, this study does not address fish survival or provide any confirmation that plug sampling is a non-lethal technique. The advantages of the plug sampling alternative are reduced if fish survival is significantly compromised. Additional research on fish survival after plug removal is warranted, and in particular, more information is needed on survival differences between species and life stages under various ambient conditions upon release.

**Table 8:** Summary of advantages and disadvantages associated with fillet versus fish plug sampling

Advantages	Disadvantages
*Fillets*	
Tissue quantities are sufficient for analysis of multiple contaminants, along with the corresponding QC samples in each analytical method, and for archive There is no field preparation of tissue samples Tissue is prepared in the laboratory under controlled conditions (limiting contamination potential) Whole fish sampling for fillet analysis provides consistency with historical studies in tissue fraction and preparation Whole fish sampling for fillet analysis provides continuity with previous national surveys for trends analysis and comparison of contaminant concentrations	Fish are killed for analysis Whole fish can be heavy and more expensive to ship Laboratory preparation (i.e., filleting and homogenization) is needed, and project-specific training as well as hands-on practice is required for laboratory staff Ample laboratory freezer space is needed for temporary storage of whole fish samples and long-term storage of archived tissue samples
*Plugs*	
Fish may be released alive (although survival rates are generally unknown) Samples are lighter and less expensive to ship No laboratory preparation of the fillet tissue is required before sample digestion and analysis Less laboratory freezer space is required for temporary sample storage	Sample volumes are insufficient for analysis of multiple chemicals and QC samples, or for repeating analyses if problems develop Sampling may require larger fish if survival of released fish is a goal Project-specific training as well as hands-on practice is required for field staff in an effort to minimize fish mortality Samples may be vulnerable to contamination during field collection and preparation An additional plug is required (for solids) if dry-weight reporting is intended Long-term frozen storage may result in moisture loss that will increase the contaminant concentration by reducing the wet weight of the sample Tissue fraction and preparation methods are not consistent with national program needs for comparison and trends analysis

## Supplementary Information

Below is the link to the electronic supplementary material.

Supplementary file1 (PDF 712 KB)

## References

[CR1] Ackerson JR, McKee MJ, Schmitt CJ, Brumbaugh WG (2014). Implementation of a non-lethal biopsy punch monitoring program for mercury in Smallmouth bass, *Micropterus dolomieu* Lacepède, from the Eleven Point River, Missouri. Environ Contam Toxicol.

[CR2] APHA (2005). Standard method 2450G. Total, fixed, and volatile solids in solids and semisolid samples. Standard methods for the examination of water and wastewater.

[CR3] Baker RF, Blanchfield PJ, Paterson MJ, Flett RJ, Wesson L (2004). Evaluation of nonlethal methods for the analysis of mercury in fish tissue. Trans Am Fish Soc.

[CR4] Bauch NJ (2007) Selenium and mercury concentrations in fish, Wolford mountain reservoir, Colorado, 2005. USGS Scientific Investigations Report 2007–5019

[CR5] Cizdziel JV, Hinniers TA, Pollard JE, Heithmar EM, Cross CL (2002). Mercury concentrations in fish from Lake Mead, USA, related to fish size, condition, tropic level, location, and consumption risk. Arch Environ Contam Toxicol.

[CR6] Crawford BA, Leider SA, Tipping JM (1977). Technique for rapidly taking samples of skeletal muscle from live adult steelhead trout. Prog Fish Culturist.

[CR7] Heltsley RM, Cope WG, Shea D, Bringolf RB, Kwak TJ, Malindzak EG (2005). Assessing organic contaminants in fish: comparison of a nonlethal tissue sampling technique to mobile and stationary passive sampling devices. Environ Sci Technol.

[CR8] Knight A, Bhavsar SP, Branfireun BA, Drouin P, Prashad R, Petro S, Oke M (2019). A comparison of fish tissue mercury concentrations from homogenized fillet and nonlethal biopsy plugs. J Environ Sci.

[CR9] Levene H, Olkin I (1960). Robust tests for equality of variances. Contributions to probability and statistics: essays in Honor of Harold hotelling.

[CR10] May TW, Brumbaugh WG (2007) Determination of total mercury in whole-body fish and fish muscle plugs collected from the South Fork of the Humboldt River, Nevada, September 2005. U.S. Geological Survey Open-File Report 2007–1059

[CR11] May TW, Walther MJ, Brumbaugh WG, McKee MJ (2013) Concentrations of elements in fish fillets, fish muscle plugs, and crayfish from the 2011 Missouri Department of Conservation general contaminant monitoring program. U.S. Geological Survey Open-File Report 2012–1268

[CR12] Morizot DC, Schmidt ME, Carmichael GJ, Stock DW, Williamson JH, Whitmore DH (1990). Minimally invasive tissue sampling. Electrophoretic and isoelectric focusing techniques in fisheries management.

[CR13] Osmundson BC, Lusk JD (2019). Field assessment of Colorado pikeminnow exposure to mercury within its designated critical habitat in Colorado, Utah, and New Mexico. Arch Environ Contam Toxicol.

[CR14] Osmundson BC, May TW, Osmundson DB (2000). Selenium concentrations in the Colorado Pikeminnow (*Ptychocheilus lucius*): relationship with flows in the Upper Colorado River. Arch Environ Contam Toxicol.

[CR15] Peterson SA, Sickle JV, Hughes RM, Schacher JA, Echols SF (2005). A biopsy procedure for determining filet and predicting whole-fish mercury concentration. Arch Environ Contam Toxicol.

[CR16] Piraino MN, Taylor DL (2013). Assessment of nonlethal methods for predicting muscle tissue mercury concentrations in coastal marine fishes. Arch Environ Contam Toxicol.

[CR17] Rolfhus KR, Sandheinrich MB, Wiener JG, Bailey SW, Thoreson KA, Hammerschmidt CR (2008). Analysis of fin clips as a nonlethal method for monitoring mercury in fish. Environ Sci Technol.

[CR18] Schmidt JG, Andersen EW, Ersboll BK, Nielsen ME (2016). Muscle wound healing in rainbow trout (*Oncorhynchus mykiss*). Fish Shellfish Immunol.

[CR19] Schmitt CJ, Brumbaugh WG (2007). Evaluation of potentially nonlethal sampling methods for monitoring mercury concentrations in Smallmouth Bass (*Micropterus dolomieu*). Arch Environ Contam Toxicol.

[CR20] Shapiro SS, Wilk MB (1965). An analysis of variance test for normality (complete samples). Biometrika.

[CR21] Stahl LL, Snyder BD, Olsen AR, Pitt JL (2009). Contaminants in fish tissue from US lakes and reservoirs: a national probabilistic study. Environ Monit Assess.

[CR22] Sun J, Robinson A, Davis JA, Trowbridge P, Stewart AR, Palace VP, Jackson ZJ (2017). Selenium in White Sturgeon Tissues: 2015 Sturgeon Derby. Regional Monitoring Program for Water Quality in San Francisco Bay.

[CR23] USEPA (1994). Method 200.8. Determination of trace elements in waters and wastes by inductively coupled plasma-mass spectrometry, revision 5.4.

[CR24] USEPA (1996). Method 3050B, acid digestion of sediments, sludges, and soils. Test methods for evaluating solid waste, physical/chemical methods.

[CR25] USEPA (2000) Guidance for assessing chemical contaminant data for use in fish advisories, Volume 1: Fish sampling and analysis, 3rd edn. EPA-823-B-00-007. U.S. Environmental Protection Agency, Washington

[CR26] USEPA (2001a) Appendix to Method 1631, Total Mercury in Tissue, Sludge, Sediment, and Soil by Acid Digestion and BrCl Oxidation. EPA-821-R-01-013. U.S. Environmental Protection Agency, Washington

[CR27] USEPA (2001b) Water quality criterion for the protection of human health: methylmercury. EPA-823-R-01-001. U.S. Environmental Protection Agency, Washington

[CR28] USEPA (2002) Method 1631, Revision E: Mercury in Water by Oxidation, Purge and Trap, and Cold Vapor Atomic Fluorescence Spectrometry. EPA-821-R-02-019. U.S. Environmental Protection Agency, Washington

[CR29] USEPA (2013) National Listing of Fish Advisories. EPA-820-F-13-058. U.S. Environmental Protection Agency, Washington

[CR30] USEPA (2015) National Coastal Condition Assessment 2010. EPA 841-R-15-006. U.S. Environmental Protection Agency, Washington

[CR31] USEPA (2016a) Aquatic Life Ambient Water Quality Criterion for Selenium – Freshwater. EPA 822-R-16-006. U.S. Environmental Protection Agency, Washington

[CR32] USEPA (2016b) National Rivers and Streams Assessment 2008–2009: A Collaborative Survey. EPA 841-R-16-007. U.S. Environmental Protection Agency, Washington

[CR33] Uthe JF (1971). A simple field technique for obtaining small samples of muscle from living fish. J Fish Res Board Can.

[CR34] Waddell B, May T (1995). Selenium concentrations in the Razorback Sucker (*Xyrauchen texanus*): Substitution of non-lethal muscle plugs for muscle tissue in contaminant assessment. Arch Environ Contam Toxicol.

