# Supplementary material for: An Evaluation of Fish Tissue Monitoring Alternatives for Mercury and Selenium: Fish Muscle Biopsy Samples Versus Homogenized Whole Fillets
Source: Arch Environ Contam Toxicol. 2021 Jul 30;81(2):236–54. doi: 10.1007/s00244-021-00872-w (PMC8342331; doi:10.1007/s00244-021-00872-w)
Supplement: Supplementary file 1 — Supplementary file1 (PDF 712 KB) [file 244_2021_872_MOESM1_ESM.pdf]

Supplementary Information

Table SI1. Fish Plug Evaluation Study fillet plug sample (FP) and homogenized fillet sample (HF) mercury data

Sample ID	Sample Type	Analyte	MDL	QL	Result (ng/g)	Units	Sample Weight (g)	Site-Specimen ID	Specimen Replicate Number	Sample Replicate Number	Water Body Name	Species
GLERWA01FP1	FP	Mercury	0.43	4.8	141	ng/g	0.831	GLERWA01	1	1	Erie	Walleye
GLERWA01FP2	FP	Mercury	0.31	3.5	141	ng/g	1.144	GLERWA01	1	2	Erie	Walleye
GLERWA01FP3	FP	Mercury	0.37	4.2	144	ng/g	0.962	GLERWA01	1	3	Erie	Walleye
GLERWA01FP4	FP	Mercury	0.35	3.9	138	ng/g	1.031	GLERWA01	1	4	Erie	Walleye
GLERWA01FP5	FP	Mercury	0.43	4.8	125	ng/g	0.834	GLERWA01	1	5	Erie	Walleye
GLERWA02FP1	FP	Mercury	0.29	3.2	91.2	ng/g	1.263	GLERWA02	2	1	Erie	Walleye
GLERWA02FP2	FP	Mercury	0.26	2.9	90.9	ng/g	1.361	GLERWA02	2	2	Erie	Walleye
GLERWA02FP3	FP	Mercury	0.29	3.2	86.7	ng/g	1.251	GLERWA02	2	3	Erie	Walleye
GLERWA02FP4	FP	Mercury	0.30	3.3	91.3	ng/g	1.207	GLERWA02	2	4	Erie	Walleye
GLERWA02FP5	FP	Mercury	0.22	2.5	89	ng/g	1.619	GLERWA02	2	5	Erie	Walleye
GLERWA03FP1	FP	Mercury	0.23	2.6	96	ng/g	1.566	GLERWA03	3	1	Erie	Walleye
GLERWA03FP2	FP	Mercury	0.25	2.8	101	ng/g	1.450	GLERWA03	3	2	Erie	Walleye
GLERWA03FP3	FP	Mercury	0.28	3.1	98.4	ng/g	1.303	GLERWA03	3	3	Erie	Walleye
GLERWA03FP4	FP	Mercury	0.21	2.3	96.4	ng/g	1.752	GLERWA03	3	4	Erie	Walleye
GLERWA03FP5	FP	Mercury	0.22	2.4	99.8	ng/g	1.652	GLERWA03	3	5	Erie	Walleye
GLERWA04FP1	FP	Mercury	0.24	2.6	123	ng/g	1.524	GLERWA04	4	1	Erie	Walleye
GLERWA04FP2	FP	Mercury	0.27	3	118	ng/g	1.349	GLERWA04	4	2	Erie	Walleye
GLERWA04FP3	FP	Mercury	0.26	2.9	120	ng/g	1.376	GLERWA04	4	3	Erie	Walleye
GLERWA04FP4	FP	Mercury	0.26	2.8	116	ng/g	1.411	GLERWA04	4	4	Erie	Walleye
GLERWA04FP5	FP	Mercury	0.28	3.1	111	ng/g	1.300	GLERWA04	4	5	Erie	Walleye
GLERWA05FP1	FP	Mercury	0.28	3.1	218	ng/g	1.277	GLERWA05	5	1	Erie	Walleye
GLERWA05FP2	FP	Mercury	0.26	2.9	213	ng/g	1.383	GLERWA05	5	2	Erie	Walleye
GLERWA05FP3	FP	Mercury	0.29	3.2	210	ng/g	1.239	GLERWA05	5	3	Erie	Walleye
GLERWA05FP4	FP	Mercury	0.34	3.8	210	ng/g	1.045	GLERWA05	5	4	Erie	Walleye
GLERWA05FP5	FP	Mercury	0.34	3.8	215	ng/g	1.050	GLERWA05	5	5	Erie	Walleye
GLERWA06FP1	FP	Mercury	0.40	4.4	642	ng/g	0.910	GLERWA06	6	1	Erie	Walleye
GLERWA06FP2	FP	Mercury	0.33	3.7	637	ng/g	1.082	GLERWA06	6	2	Erie	Walleye
GLERWA06FP3	FP	Mercury	0.34	3.7	649	ng/g	1.069	GLERWA06	6	3	Erie	Walleye
GLERWA06FP4	FP	Mercury	0.35	3.9	643	ng/g	1.019	GLERWA06	6	4	Erie	Walleye
GLERWA06FP5	FP	Mercury	0.36	4	635	ng/g	0.989	GLERWA06	6	5	Erie	Walleye
GLERWA07FP1	FP	Mercury	0.30	3.4	99.4	ng/g	1.181	GLERWA07	7	1	Erie	Walleye
GLERWA07FP2	FP	Mercury	0.29	3.3	103	ng/g	1.223	GLERWA07	7	2	Erie	Walleye
GLERWA07FP3	FP	Mercury	0.26	2.9	101	ng/g	1.396	GLERWA07	7	3	Erie	Walleye
GLERWA07FP4	FP	Mercury	0.23	2.5	103	ng/g	1.573	GLERWA07	7	4	Erie	Walleye
GLERWA07FP5	FP	Mercury	0.24	2.7	109	ng/g	1.492	GLERWA07	7	5	Erie	Walleye
GLERWA08FP1	FP	Mercury	0.44	4.9	158	ng/g	0.814	GLERWA08	8	1	Erie	Walleye
GLERWA08FP2	FP	Mercury	0.38	4.2	132	ng/g	0.942	GLERWA08	8	2	Erie	Walleye
GLERWA08FP3	FP	Mercury	0.33	3.6	164	ng/g	1.107	GLERWA08	8	3	Erie	Walleye
GLERWA08FP4	FP	Mercury	0.34	3.8	161	ng/g	1.046	GLERWA08	8	4	Erie	Walleye
GLERWA08FP5	FP	Mercury	0.42	4.7	165	ng/g	0.856	GLERWA08	8	5	Erie	Walleye

Sample ID	Sample Type	Analyte	MDL	QL	Result (ng/g)	Units	Sample Weight (g)	Site-Specimen ID	Specimen Replicate Number	Sample Replicate Number	Water Body Name	Species
GLERWA09FP1	FP	Mercury	0.25	2.8	113	ng/g	1.416	GLERWA09	9	1	Erie	Walleye
GLERWA09FP2	FP	Mercury	0.26	2.9	112	ng/g	1.399	GLERWA09	9	2	Erie	Walleye
GLERWA09FP3	FP	Mercury	0.28	3.1	109	ng/g	1.300	GLERWA09	9	3	Erie	Walleye
GLERWA09FP4	FP	Mercury	0.26	2.9	115	ng/g	1.370	GLERWA09	9	4	Erie	Walleye
GLERWA09FP5	FP	Mercury	0.25	2.7	113	ng/g	1.469	GLERWA09	9	5	Erie	Walleye
GLERWA10FP1	FP	Mercury	0.35	3.9	306	ng/g	1.029	GLERWA10	10	1	Erie	Walleye
GLERWA10FP2	FP	Mercury	0.35	3.9	308	ng/g	1.020	GLERWA10	10	2	Erie	Walleye
GLERWA10FP3	FP	Mercury	0.36	4	320	ng/g	0.993	GLERWA10	10	3	Erie	Walleye
GLERWA10FP4	FP	Mercury	0.28	3.2	326	ng/g	1.267	GLERWA10	10	4	Erie	Walleye
GLERWA10FP5	FP	Mercury	0.35	3.9	304	ng/g	1.014	GLERWA10	10	5	Erie	Walleye
GLMILT01FP1	FP	Mercury	0.40	4.4	82.6	ng/g	0.908	GLMILT01	1	1	Michigan	Lake trout
GLMILT01FP2	FP	Mercury	0.41	4.5	84.7	ng/g	0.885	GLMILT01	1	2	Michigan	Lake trout
GLMILT01FP3	FP	Mercury	0.38	4.2	85.1	ng/g	0.958	GLMILT01	1	3	Michigan	Lake trout
GLMILT01FP4	FP	Mercury	0.33	3.7	92.1	ng/g	1.086	GLMILT01	1	4	Michigan	Lake trout
GLMILT01FP5	FP	Mercury	0.43	4.8	93.2	ng/g	0.838	GLMILT01	1	5	Michigan	Lake trout
GLMILT02FP1	FP	Mercury	0.51	5.7	144	ng/g	0.701	GLMILT02	2	1	Michigan	Lake trout
GLMILT02FP2	FP	Mercury	0.58	6.5	170	ng/g	0.619	GLMILT02	2	2	Michigan	Lake trout
GLMILT02FP3	FP	Mercury	0.52	5.8	141	ng/g	0.687	GLMILT02	2	3	Michigan	Lake trout
GLMILT02FP4	FP	Mercury	0.48	5.3	167	ng/g	0.756	GLMILT02	2	4	Michigan	Lake trout
GLMILT02FP5	FP	Mercury	0.46	5.1	161	ng/g	0.789	GLMILT02	2	5	Michigan	Lake trout
GLMILT03FP1	FP	Mercury	0.33	3.7	89.2	ng/g	1.085	GLMILT03	3	1	Michigan	Lake trout
GLMILT03FP2	FP	Mercury	0.32	3.6	82.4	ng/g	1.119	GLMILT03	3	2	Michigan	Lake trout
GLMILT03FP3	FP	Mercury	0.32	3.6	82.3	ng/g	1.118	GLMILT03	3	3	Michigan	Lake trout
GLMILT03FP4	FP	Mercury	0.33	3.6	94.1	ng/g	1.096	GLMILT03	3	4	Michigan	Lake trout
GLMILT03FP5	FP	Mercury	0.33	3.7	89.9	ng/g	1.080	GLMILT03	3	5	Michigan	Lake trout
GLMILT04FP1	FP	Mercury	0.35	3.9	153	ng/g	1.032	GLMILT04	4	1	Michigan	Lake trout
GLMILT04FP2	FP	Mercury	0.46	5.2	137	ng/g	0.775	GLMILT04	4	2	Michigan	Lake trout
GLMILT04FP3	FP	Mercury	0.47	5.3	142	ng/g	0.760	GLMILT04	4	3	Michigan	Lake trout
GLMILT04FP4	FP	Mercury	0.36	4	153	ng/g	0.990	GLMILT04	4	4	Michigan	Lake trout
GLMILT04FP5	FP	Mercury	0.35	3.8	157	ng/g	1.041	GLMILT04	4	5	Michigan	Lake trout
GLMILT05FP1	FP	Mercury	0.32	3.6	171	ng/g	1.110	GLMILT05	5	1	Michigan	Lake trout
GLMILT05FP2	FP	Mercury	0.36	4	174	ng/g	0.990	GLMILT05	5	2	Michigan	Lake trout
GLMILT05FP3	FP	Mercury	0.35	3.9	157	ng/g	1.015	GLMILT05	5	3	Michigan	Lake trout
GLMILT05FP4	FP	Mercury	0.27	3	162	ng/g	1.344	GLMILT05	5	4	Michigan	Lake trout
GLMILT05FP5	FP	Mercury	0.35	3.9	166	ng/g	1.029	GLMILT05	5	5	Michigan	Lake trout
GLMILT06FP1	FP	Mercury	0.28	3.1	131	ng/g	1.274	GLMILT06	6	1	Michigan	Lake trout
GLMILT06FP2	FP	Mercury	0.32	3.5	135	ng/g	1.141	GLMILT06	6	2	Michigan	Lake trout
GLMILT06FP3	FP	Mercury	0.35	3.9	143	ng/g	1.019	GLMILT06	6	3	Michigan	Lake trout
GLMILT06FP4	FP	Mercury	0.30	3.3	131	ng/g	1.216	GLMILT06	6	4	Michigan	Lake trout
GLMILT06FP5	FP	Mercury	0.33	3.6	150	ng/g	1.104	GLMILT06	6	5	Michigan	Lake trout

Sample ID	Sample Type	Analyte	MDL	QL	Result (ng/g)	Units	Sample Weight (g)	Site-Specimen ID	Specimen Replicate Number	Sample Replicate Number	Water Body Name	Species
GLMILT07FP1	FP	Mercury	0.53	5.9	98.7	ng/g	0.679	GLMILT07	7	1	Michigan	Lake trout
GLMILT07FP2	FP	Mercury	0.60	6.7	92.8	ng/g	0.598	GLMILT07	7	2	Michigan	Lake trout
GLMILT07FP3	FP	Mercury	0.54	6	105	ng/g	0.672	GLMILT07	7	3	Michigan	Lake trout
GLMILT07FP4	FP	Mercury	0.44	4.9	89.5	ng/g	0.822	GLMILT07	7	4	Michigan	Lake trout
GLMILT07FP5	FP	Mercury	0.37	4.1	108	ng/g	0.970	GLMILT07	7	5	Michigan	Lake trout
GLMILT08FP1	FP	Mercury	0.42	4.6	82.9	ng/g	0.863	GLMILT08	8	1	Michigan	Lake trout
GLMILT08FP2	FP	Mercury	0.36	4	86.3	ng/g	1.009	GLMILT08	8	2	Michigan	Lake trout
GLMILT08FP3	FP	Mercury	0.36	4	82.9	ng/g	0.991	GLMILT08	8	3	Michigan	Lake trout
GLMILT08FP4	FP	Mercury	0.36	4	81	ng/g	0.996	GLMILT08	8	4	Michigan	Lake trout
GLMILT08FP5	FP	Mercury	0.38	4.2	102	ng/g	0.943	GLMILT08	8	5	Michigan	Lake trout
GLMILT09FP1	FP	Mercury	0.28	3.1	131	ng/g	1.288	GLMILT09	9	1	Michigan	Lake trout
GLMILT09FP2	FP	Mercury	0.29	3.2	125	ng/g	1.257	GLMILT09	9	2	Michigan	Lake trout
GLMILT09FP3	FP	Mercury	0.28	3.1	128	ng/g	1.290	GLMILT09	9	3	Michigan	Lake trout
GLMILT09FP4	FP	Mercury	0.28	3.1	116	ng/g	1.286	GLMILT09	9	4	Michigan	Lake trout
GLMILT09FP5	FP	Mercury	0.27	3	116	ng/g	1.317	GLMILT09	9	5	Michigan	Lake trout
GLMILT10FP1	FP	Mercury	0.32	3.5	208	ng/g	1.129	GLMILT10	10	1	Michigan	Lake trout
GLMILT10FP2	FP	Mercury	0.30	3.3	211	ng/g	1.207	GLMILT10	10	2	Michigan	Lake trout
GLMILT10FP3	FP	Mercury	0.27	3	200	ng/g	1.328	GLMILT10	10	3	Michigan	Lake trout
GLMILT10FP4	FP	Mercury	0.30	3.3	206	ng/g	1.211	GLMILT10	10	4	Michigan	Lake trout
GLMILT10FP5	FP	Mercury	0.34	3.7	201	ng/g	1.072	GLMILT10	10	5	Michigan	Lake trout
GLONSA01FP1	FP	Mercury	0.31	3.4	115	ng/g	1.175	GLONSA01	1	1	Ontario	Chinook salmon
GLONSA01FP2	FP	Mercury	0.30	3.4	118	ng/g	1.182	GLONSA01	1	2	Ontario	Chinook salmon
GLONSA01FP3	FP	Mercury	0.34	3.8	115	ng/g	1.055	GLONSA01	1	3	Ontario	Chinook salmon
GLONSA01FP4	FP	Mercury	0.31	3.4	119	ng/g	1.169	GLONSA01	1	4	Ontario	Chinook salmon
GLONSA01FP5	FP	Mercury	0.29	3.2	123	ng/g	1.238	GLONSA01	1	5	Ontario	Chinook salmon
GLONSA02FP1	FP	Mercury	0.36	4	107	ng/g	1.008	GLONSA02	2	1	Ontario	Chinook salmon
GLONSA02FP2	FP	Mercury	0.30	3.3	112	ng/g	1.214	GLONSA02	2	2	Ontario	Chinook salmon
GLONSA02FP3	FP	Mercury	0.34	3.8	112	ng/g	1.054	GLONSA02	2	3	Ontario	Chinook salmon
GLONSA02FP4	FP	Mercury	0.31	3.4	113	ng/g	1.161	GLONSA02	2	4	Ontario	Chinook salmon
GLONSA02FP5	FP	Mercury	0.30	3.3	115	ng/g	1.207	GLONSA02	2	5	Ontario	Chinook salmon
GLONSA03FP1	FP	Mercury	0.33	3.6	122	ng/g	1.101	GLONSA03	3	1	Ontario	Chinook salmon
GLONSA03FP2	FP	Mercury	0.30	3.3	120	ng/g	1.198	GLONSA03	3	2	Ontario	Chinook salmon
GLONSA03FP3	FP	Mercury	0.37	4.1	117	ng/g	0.980	GLONSA03	3	3	Ontario	Chinook salmon
GLONSA03FP4	FP	Mercury	0.36	4.1	118	ng/g	0.987	GLONSA03	3	4	Ontario	Chinook salmon
GLONSA03FP5	FP	Mercury	0.27	3	118	ng/g	1.339	GLONSA03	3	5	Ontario	Chinook salmon
GLONSA04FP1	FP	Mercury	0.40	4.4	106	ng/g	0.908	GLONSA04	4	1	Ontario	Chinook salmon
GLONSA04FP2	FP	Mercury	0.32	3.5	111	ng/g	1.129	GLONSA04	4	2	Ontario	Chinook salmon
GLONSA04FP3	FP	Mercury	0.28	3.1	117	ng/g	1.275	GLONSA04	4	3	Ontario	Chinook salmon
GLONSA04FP4	FP	Mercury	0.35	3.9	107	ng/g	1.035	GLONSA04	4	4	Ontario	Chinook salmon
GLONSA04FP5	FP	Mercury	0.26	2.9	121	ng/g	1.382	GLONSA04	4	5	Ontario	Chinook salmon

Sample ID	Sample Type	Analyte	MDL	QL	Result (ng/g)	Units	Sample Weight (g)	Site-Specimen ID	Specimen Replicate Number	Sample Replicate Number	Water Body Name	Species
GLONSA05FP1	FP	Mercury	0.29	3.2	130	ng/g	1.233	GLONSA05	5	1	Ontario	Chinook salmon
GLONSA05FP2	FP	Mercury	0.29	3.3	129	ng/g	1.227	GLONSA05	5	2	Ontario	Chinook salmon
GLONSA05FP3	FP	Mercury	0.31	3.4	134	ng/g	1.170	GLONSA05	5	3	Ontario	Chinook salmon
GLONSA05FP4	FP	Mercury	0.27	3	135	ng/g	1.322	GLONSA05	5	4	Ontario	Chinook salmon
GLONSA05FP5	FP	Mercury	0.28	3.1	140	ng/g	1.289	GLONSA05	5	5	Ontario	Chinook salmon
GLONSA06FP1	FP	Mercury	0.32	3.5	110	ng/g	1.140	GLONSA06	6	1	Ontario	Chinook salmon
GLONSA06FP2	FP	Mercury	0.27	3	119	ng/g	1.321	GLONSA06	6	2	Ontario	Chinook salmon
GLONSA06FP3	FP	Mercury	0.36	3.9	118	ng/g	1.014	GLONSA06	6	3	Ontario	Chinook salmon
GLONSA06FP4	FP	Mercury	0.34	3.7	119	ng/g	1.071	GLONSA06	6	4	Ontario	Chinook salmon
GLONSA06FP5	FP	Mercury	0.29	3.2	126	ng/g	1.246	GLONSA06	6	5	Ontario	Chinook salmon
GLONSA07FP1	FP	Mercury	0.38	4.2	125	ng/g	0.956	GLONSA07	7	1	Ontario	Chinook salmon
GLONSA07FP2	FP	Mercury	0.31	3.4	137	ng/g	1.164	GLONSA07	7	2	Ontario	Chinook salmon
GLONSA07FP3	FP	Mercury	0.31	3.5	133	ng/g	1.144	GLONSA07	7	3	Ontario	Chinook salmon
GLONSA07FP4	FP	Mercury	0.38	4.2	143	ng/g	0.954	GLONSA07	7	4	Ontario	Chinook salmon
GLONSA07FP5	FP	Mercury	0.28	3.1	143	ng/g	1.272	GLONSA07	7	5	Ontario	Chinook salmon
GLONSA08FP1	FP	Mercury	0.28	3.1	109	ng/g	1.281	GLONSA08	8	1	Ontario	Chinook salmon
GLONSA08FP2	FP	Mercury	0.26	2.9	114	ng/g	1.384	GLONSA08	8	2	Ontario	Chinook salmon
GLONSA08FP3	FP	Mercury	0.30	3.4	116	ng/g	1.194	GLONSA08	8	3	Ontario	Chinook salmon
GLONSA08FP4	FP	Mercury	0.23	2.6	119	ng/g	1.534	GLONSA08	8	4	Ontario	Chinook salmon
GLONSA08FP5	FP	Mercury	0.29	3.2	116	ng/g	1.247	GLONSA08	8	5	Ontario	Chinook salmon
GLONSA09FP1	FP	Mercury	0.28	3.1	137	ng/g	1.289	GLONSA09	9	1	Ontario	Chinook salmon
GLONSA09FP2	FP	Mercury	0.27	3	137	ng/g	1.345	GLONSA09	9	2	Ontario	Chinook salmon
GLONSA09FP3	FP	Mercury	0.31	3.4	136	ng/g	1.172	GLONSA09	9	3	Ontario	Chinook salmon
GLONSA09FP4	FP	Mercury	0.19	2.1	142	ng/g	1.931	GLONSA09	9	4	Ontario	Chinook salmon
GLONSA09FP5	FP	Mercury	0.23	2.5	142	ng/g	1.598	GLONSA09	9	5	Ontario	Chinook salmon
GLONSA10FP1	FP	Mercury	0.29	3.2	120	ng/g	1.262	GLONSA10	10	1	Ontario	Chinook salmon
GLONSA10FP2	FP	Mercury	0.27	3	120	ng/g	1.352	GLONSA10	10	2	Ontario	Chinook salmon
GLONSA10FP3	FP	Mercury	0.25	2.8	120	ng/g	1.432	GLONSA10	10	3	Ontario	Chinook salmon
GLONSA10FP4	FP	Mercury	0.29	3.3	124	ng/g	1.225	GLONSA10	10	4	Ontario	Chinook salmon
GLONSA10FP5	FP	Mercury	0.28	3.1	121	ng/g	1.293	GLONSA10	10	5	Ontario	Chinook salmon
RVANBC01FP1	FP	Mercury	0.78	8.7	118	ng/g	0.461	RVANBC01	1	1	Anacostia	Blue catfish
RVANBC01FP2	FP	Mercury	0.60	6.7	126	ng/g	0.601	RVANBC01	1	2	Anacostia	Blue catfish
RVANBC01FP3	FP	Mercury	0.69	7.7	129	ng/g	0.518	RVANBC01	1	3	Anacostia	Blue catfish
RVANBC01FP4	FP	Mercury	0.60	6.7	130	ng/g	0.598	RVANBC01	1	4	Anacostia	Blue catfish
RVANBC01FP5	FP	Mercury	0.53	5.9	136	ng/g	0.683	RVANBC01	1	5	Anacostia	Blue catfish
RVANBC02FP1	FP	Mercury	0.49	5.4	66.2	ng/g	0.737	RVANBC02	2	1	Anacostia	Blue catfish
RVANBC02FP2	FP	Mercury	0.50	5.6	64.1	ng/g	0.713	RVANBC02	2	2	Anacostia	Blue catfish
RVANBC02FP3	FP	Mercury	0.62	6.9	60.2	ng/g	0.583	RVANBC02	2	3	Anacostia	Blue catfish
RVANBC02FP4	FP	Mercury	0.91	10.2	58	ng/g	0.394	RVANBC02	2	4	Anacostia	Blue catfish
RVANBC02FP5	FP	Mercury	0.48	5.3	63.9	ng/g	0.756	RVANBC02	2	5	Anacostia	Blue catfish

Sample ID	Sample Type	Analyte	MDL	QL	Result (ng/g)	Units	Sample Weight (g)	Site-Specimen ID	Specimen Replicate Number	Sample Replicate Number	Water Body Name	Species
RVANBC03FP1	FP	Mercury	0.46	5.1	78.8	ng/g	0.783	RVANBC03	3	1	Anacostia	Blue catfish
RVANBC03FP2	FP	Mercury	0.52	5.8	69.6	ng/g	0.692	RVANBC03	3	2	Anacostia	Blue catfish
RVANBC03FP3	FP	Mercury	0.49	5.4	82.5	ng/g	0.734	RVANBC03	3	3	Anacostia	Blue catfish
RVANBC03FP4	FP	Mercury	0.43	4.7	80.5	ng/g	0.844	RVANBC03	3	4	Anacostia	Blue catfish
RVANBC03FP5	FP	Mercury	0.44	4.8	87.9	ng/g	0.825	RVANBC03	3	5	Anacostia	Blue catfish
RVANBC04FP1	FP	Mercury	0.59	6.6	44.2	ng/g	0.607	RVANBC04	4	1	Anacostia	Blue catfish
RVANBC04FP2	FP	Mercury	0.63	7	48.5	ng/g	0.569	RVANBC04	4	2	Anacostia	Blue catfish
RVANBC04FP3	FP	Mercury	0.62	6.8	48.1	ng/g	0.585	RVANBC04	4	3	Anacostia	Blue catfish
RVANBC04FP4	FP	Mercury	0.51	5.7	51.1	ng/g	0.702	RVANBC04	4	4	Anacostia	Blue catfish
RVANBC04FP5	FP	Mercury	0.53	5.9	50.5	ng/g	0.674	RVANBC04	4	5	Anacostia	Blue catfish
RVANBC05FP1	FP	Mercury	0.48	5.3	102	ng/g	0.749	RVANBC05	5	1	Anacostia	Blue catfish
RVANBC05FP2	FP	Mercury	0.52	5.7	101	ng/g	0.699	RVANBC05	5	2	Anacostia	Blue catfish
RVANBC05FP3	FP	Mercury	0.46	5.1	108	ng/g	0.787	RVANBC05	5	3	Anacostia	Blue catfish
RVANBC05FP4	FP	Mercury	0.56	6.2	96.8	ng/g	0.643	RVANBC05	5	4	Anacostia	Blue catfish
RVANBC05FP5	FP	Mercury	0.53	5.9	106	ng/g	0.677	RVANBC05	5	5	Anacostia	Blue catfish
RVANBC06FP1	FP	Mercury	0.49	5.4	241	ng/g	0.737	RVANBC06	6	1	Anacostia	Blue catfish
RVANBC06FP2	FP	Mercury	0.55	6.2	265	ng/g	0.649	RVANBC06	6	2	Anacostia	Blue catfish
RVANBC06FP3	FP	Mercury	0.41	4.6	271	ng/g	0.879	RVANBC06	6	3	Anacostia	Blue catfish
RVANBC06FP4	FP	Mercury	0.47	5.2	308	ng/g	0.772	RVANBC06	6	4	Anacostia	Blue catfish
RVANBC06FP5	FP	Mercury	0.50	5.5	276	ng/g	0.721	RVANBC06	6	5	Anacostia	Blue catfish
RVANBC07FP1	FP	Mercury	0.64	7.1	63	ng/g	0.564	RVANBC07	7	1	Anacostia	Blue catfish
RVANBC07FP2	FP	Mercury	0.81	9	65.7	ng/g	0.443	RVANBC07	7	2	Anacostia	Blue catfish
RVANBC07FP3	FP	Mercury	0.58	6.5	68.5	ng/g	0.616	RVANBC07	7	3	Anacostia	Blue catfish
RVANBC07FP4	FP	Mercury	0.47	5.2	80.2	ng/g	0.766	RVANBC07	7	4	Anacostia	Blue catfish
RVANBC07FP5	FP	Mercury	0.60	6.6	70.1	ng/g	0.603	RVANBC07	7	5	Anacostia	Blue catfish
RVANBC08FP1	FP	Mercury	0.46	5.1	96.2	ng/g	0.784	RVANBC08	8	1	Anacostia	Blue catfish
RVANBC08FP2	FP	Mercury	0.52	5.7	87.1	ng/g	0.696	RVANBC08	8	2	Anacostia	Blue catfish
RVANBC08FP3	FP	Mercury	0.40	4.5	107	ng/g	0.891	RVANBC08	8	3	Anacostia	Blue catfish
RVANBC08FP4	FP	Mercury	0.25	2.8	98.6	ng/g	1.429	RVANBC08	8	4	Anacostia	Blue catfish
RVANBC08FP5	FP	Mercury	0.29	3.2	105	ng/g	1.247	RVANBC08	8	5	Anacostia	Blue catfish
RVANBC09FP1	FP	Mercury	0.45	5	81.9	ng/g	0.806	RVANBC09	9	1	Anacostia	Blue catfish
RVANBC09FP2	FP	Mercury	0.52	5.7	74	ng/g	0.699	RVANBC09	9	2	Anacostia	Blue catfish
RVANBC09FP3	FP	Mercury	0.51	5.7	88.5	ng/g	0.702	RVANBC09	9	3	Anacostia	Blue catfish
RVANBC09FP4	FP	Mercury	0.53	5.9	75.9	ng/g	0.678	RVANBC09	9	4	Anacostia	Blue catfish
RVANBC09FP5	FP	Mercury	0.56	6.3	82.9	ng/g	0.639	RVANBC09	9	5	Anacostia	Blue catfish
RVANBC10FP1	FP	Mercury	0.57	6.4	128	ng/g	0.627	RVANBC10	10	1	Anacostia	Blue catfish
RVANBC10FP2	FP	Mercury	0.64	7.1	136	ng/g	0.561	RVANBC10	10	2	Anacostia	Blue catfish
RVANBC10FP3	FP	Mercury	0.46	5.1	125	ng/g	0.778	RVANBC10	10	3	Anacostia	Blue catfish
RVANBC10FP4	FP	Mercury	0.51	5.6	140	ng/g	0.712	RVANBC10	10	4	Anacostia	Blue catfish
RVANBC10FP5	FP	Mercury	0.41	4.5	131	ng/g	0.886	RVANBC10	10	5	Anacostia	Blue catfish

Sample ID	Sample Type	Analyte	MDL	QL	Result (ng/g)	Units	Sample Weight (g)	Site-Specimen ID	Specimen Replicate Number	Sample Replicate Number	Water Body Name	Species
RVPTLB01FP1	FP	Mercury	0.51	5.6	185	ng/g	1.708	RVPTLB01	1	1	Potomac	Largemouth bass
RVPTLB01FP2	FP	Mercury	0.31	3.4	186	ng/g	1.173	RVPTLB01	1	2	Potomac	Largemouth bass
RVPTLB01FP3	FP	Mercury	0.48	5.3	193	ng/g	0.752	RVPTLB01	1	3	Potomac	Largemouth bass
RVPTLB01FP4	FP	Mercury	0.45	5	183	ng/g	0.801	RVPTLB01	1	4	Potomac	Largemouth bass
RVPTLB01FP5	FP	Mercury	0.45	5	187	ng/g	0.808	RVPTLB01	1	5	Potomac	Largemouth bass
RVPTLB02FP1	FP	Mercury	0.50	5.5	78.2	ng/g	0.723	RVPTLB02	2	1	Potomac	Largemouth bass
RVPTLB02FP2	FP	Mercury	0.54	6	74	ng/g	0.666	RVPTLB02	2	2	Potomac	Largemouth bass
RVPTLB02FP3	FP	Mercury	0.55	6.1	75.8	ng/g	0.656	RVPTLB02	2	3	Potomac	Largemouth bass
RVPTLB02FP4	FP	Mercury	0.52	5.8	74.3	ng/g	0.690	RVPTLB02	2	4	Potomac	Largemouth bass
RVPTLB02FP5	FP	Mercury	0.61	6.8	75.6	ng/g	0.591	RVPTLB02	2	5	Potomac	Largemouth bass
RVPTLB03FP1	FP	Mercury	0.44	4.8	133	ng/g	0.826	RVPTLB03	3	1	Potomac	Largemouth bass
RVPTLB03FP2	FP	Mercury	0.42	4.6	135	ng/g	0.862	RVPTLB03	3	2	Potomac	Largemouth bass
RVPTLB03FP3	FP	Mercury	0.42	4.6	131	ng/g	0.861	RVPTLB03	3	3	Potomac	Largemouth bass
RVPTLB03FP4	FP	Mercury	0.43	4.8	129	ng/g	0.840	RVPTLB03	3	4	Potomac	Largemouth bass
RVPTLB03FP5	FP	Mercury	0.42	4.7	116	ng/g	0.857	RVPTLB03	3	5	Potomac	Largemouth bass
RVPTLB04FP1	FP	Mercury	0.43	4.7	107	ng/g	0.843	RVPTLB04	4	1	Potomac	Largemouth bass
RVPTLB04FP2	FP	Mercury	0.42	4.7	107	ng/g	0.848	RVPTLB04	4	2	Potomac	Largemouth bass
RVPTLB04FP3	FP	Mercury	0.41	4.5	103	ng/g	0.880	RVPTLB04	4	3	Potomac	Largemouth bass
RVPTLB04FP4	FP	Mercury	0.41	4.6	101	ng/g	0.878	RVPTLB04	4	4	Potomac	Largemouth bass
RVPTLB04FP5	FP	Mercury	0.39	4.3	103	ng/g	0.926	RVPTLB04	4	5	Potomac	Largemouth bass
RVPTLB05FP1	FP	Mercury	0.46	5.1	84.1	ng/g	0.785	RVPTLB05	5	1	Potomac	Largemouth bass
RVPTLB05FP2	FP	Mercury	0.53	5.9	81.8	ng/g	0.683	RVPTLB05	5	2	Potomac	Largemouth bass
RVPTLB05FP3	FP	Mercury	0.50	5.5	82.5	ng/g	0.726	RVPTLB05	5	3	Potomac	Largemouth bass
RVPTLB05FP4	FP	Mercury	0.43	4.7	82	ng/g	0.847	RVPTLB05	5	4	Potomac	Largemouth bass
RVPTLB05FP5	FP	Mercury	0.40	4.4	85.5	ng/g	0.907	RVPTLB05	5	5	Potomac	Largemouth bass
RVPTLB06FP1	FP	Mercury	0.57	6.3	94.6	ng/g	0.630	RVPTLB06	6	1	Potomac	Largemouth bass
RVPTLB06FP2	FP	Mercury	0.44	4.9	96.4	ng/g	0.811	RVPTLB06	6	2	Potomac	Largemouth bass
RVPTLB06FP3	FP	Mercury	0.44	4.9	98.1	ng/g	0.813	RVPTLB06	6	3	Potomac	Largemouth bass
RVPTLB06FP4	FP	Mercury	0.51	5.7	97.4	ng/g	0.706	RVPTLB06	6	4	Potomac	Largemouth bass
RVPTLB06FP5	FP	Mercury	0.46	5.1	102	ng/g	0.782	RVPTLB06	6	5	Potomac	Largemouth bass
RVPTLB07FP1	FP	Mercury	0.28	3.1	109	ng/g	1.309	RVPTLB07	7	1	Potomac	Largemouth bass
RVPTLB07FP2	FP	Mercury	0.27	3	111	ng/g	1.344	RVPTLB07	7	2	Potomac	Largemouth bass
RVPTLB07FP3	FP	Mercury	0.26	2.9	109	ng/g	1.371	RVPTLB07	7	3	Potomac	Largemouth bass
RVPTLB07FP4	FP	Mercury	0.26	2.9	106	ng/g	1.379	RVPTLB07	7	4	Potomac	Largemouth bass
RVPTLB07FP5	FP	Mercury	0.29	3.2	102	ng/g	1.249	RVPTLB07	7	5	Potomac	Largemouth bass
RVPTLB08FP1	FP	Mercury	0.41	4.5	506	ng/g	0.883	RVPTLB08	8	1	Potomac	Largemouth bass
RVPTLB08FP2	FP	Mercury	0.33	3.6	501	ng/g	1.102	RVPTLB08	8	2	Potomac	Largemouth bass
RVPTLB08FP3	FP	Mercury	0.40	4.4	501	ng/g	0.911	RVPTLB08	8	3	Potomac	Largemouth bass
RVPTLB08FP4	FP	Mercury	0.45	5	500	ng/g	0.798	RVPTLB08	8	4	Potomac	Largemouth bass
RVPTLB08FP5	FP	Mercury	0.46	5.1	526	ng/g	0.790	RVPTLB08	8	5	Potomac	Largemouth bass

Sample ID	Sample Type	Analyte	MDL	QL	Result (ng/g)	Units	Sample Weight (g)	Site-Specimen ID	Specimen Replicate Number	Sample Replicate Number	Water Body Name	Species
RVPTLB09FP1	FP	Mercury	0.28	3.2	382	ng/g	1.269	RVPTLB09	9	1	Potomac	Largemouth bass
RVPTLB09FP2	FP	Mercury	0.36	4	376	ng/g	0.993	RVPTLB09	9	2	Potomac	Largemouth bass
RVPTLB09FP3	FP	Mercury	0.38	4.2	374	ng/g	0.949	RVPTLB09	9	3	Potomac	Largemouth bass
RVPTLB09FP4	FP	Mercury	0.33	3.7	364	ng/g	1.076	RVPTLB09	9	4	Potomac	Largemouth bass
RVPTLB09FP5	FP	Mercury	0.37	4.1	366	ng/g	0.968	RVPTLB09	9	5	Potomac	Largemouth bass
RVPTLB10FP1	FP	Mercury	0.40	4.5	108	ng/g	0.898	RVPTLB10	10	1	Potomac	Largemouth bass
RVPTLB10FP2	FP	Mercury	0.39	4.4	109	ng/g	0.915	RVPTLB10	10	2	Potomac	Largemouth bass
RVPTLB10FP3	FP	Mercury	0.40	4.5	108	ng/g	0.895	RVPTLB10	10	3	Potomac	Largemouth bass
RVPTLB10FP4	FP	Mercury	0.40	4.4	112	ng/g	0.906	RVPTLB10	10	4	Potomac	Largemouth bass
RVPTLB10FP5	FP	Mercury	0.30	3.3	120	ng/g	1.198	RVPTLB10	10	5	Potomac	Largemouth bass
RVSLSB01FP1	FP	Mercury	0.33	3.7	179	ng/g	1.087	RVSLSB01	1	1	St Lawrence	Smallmouth bass
RVSLSB01FP2	FP	Mercury	0.42	4.6	197	ng/g	0.865	RVSLSB01	1	2	St Lawrence	Smallmouth bass
RVSLSB01FP3	FP	Mercury	0.34	3.8	184	ng/g	1.054	RVSLSB01	1	3	St Lawrence	Smallmouth bass
RVSLSB01FP4	FP	Mercury	0.38	4.2	195	ng/g	0.960	RVSLSB01	1	4	St Lawrence	Smallmouth bass
RVSLSB01FP5	FP	Mercury	0.29	3.2	184	ng/g	1.260	RVSLSB01	1	5	St Lawrence	Smallmouth bass
RVSLSB02FP1	FP	Mercury	0.29	3.2	264	ng/g	1.250	RVSLSB02	2	1	St Lawrence	Smallmouth bass
RVSLSB02FP2	FP	Mercury	0.36	4.1	237	ng/g	0.987	RVSLSB02	2	2	St Lawrence	Smallmouth bass
RVSLSB02FP3	FP	Mercury	0.35	3.9	266	ng/g	1.015	RVSLSB02	2	3	St Lawrence	Smallmouth bass
RVSLSB02FP4	FP	Mercury	0.35	3.9	236	ng/g	1.034	RVSLSB02	2	4	St Lawrence	Smallmouth bass
RVSLSB02FP5	FP	Mercury	0.33	3.7	273	ng/g	1.076	RVSLSB02	2	5	St Lawrence	Smallmouth bass
RVSLSB03FP1	FP	Mercury	0.38	4.2	100	ng/g	0.955	RVSLSB03	3	1	St Lawrence	Smallmouth bass
RVSLSB03FP2	FP	Mercury	0.44	4.8	109	ng/g	0.825	RVSLSB03	3	2	St Lawrence	Smallmouth bass
RVSLSB03FP3	FP	Mercury	0.33	3.6	99.7	ng/g	1.098	RVSLSB03	3	3	St Lawrence	Smallmouth bass
RVSLSB03FP4	FP	Mercury	0.32	3.5	111	ng/g	1.133	RVSLSB03	3	4	St Lawrence	Smallmouth bass
RVSLSB03FP5	FP	Mercury	0.32	3.6	99.6	ng/g	1.113	RVSLSB03	3	5	St Lawrence	Smallmouth bass
RVSLSB04FP1	FP	Mercury	0.39	4.3	322	ng/g	0.922	RVSLSB04	4	1	St Lawrence	Smallmouth bass
RVSLSB04FP2	FP	Mercury	0.44	4.9	285	ng/g	0.810	RVSLSB04	4	2	St Lawrence	Smallmouth bass
RVSLSB04FP3	FP	Mercury	0.34	3.8	322	ng/g	1.050	RVSLSB04	4	3	St Lawrence	Smallmouth bass
RVSLSB04FP4	FP	Mercury	0.34	3.7	288	ng/g	1.074	RVSLSB04	4	4	St Lawrence	Smallmouth bass
RVSLSB04FP5	FP	Mercury	0.30	3.3	310	ng/g	1.216	RVSLSB04	4	5	St Lawrence	Smallmouth bass
RVSLSB05FP1	FP	Mercury	0.32	3.6	170	ng/g	1.117	RVSLSB05	5	1	St Lawrence	Smallmouth bass
RVSLSB05FP2	FP	Mercury	0.40	4.4	153	ng/g	0.900	RVSLSB05	5	2	St Lawrence	Smallmouth bass
RVSLSB05FP3	FP	Mercury	0.35	3.8	172	ng/g	1.040	RVSLSB05	5	3	St Lawrence	Smallmouth bass
RVSLSB05FP4	FP	Mercury	0.26	2.9	152	ng/g	1.364	RVSLSB05	5	4	St Lawrence	Smallmouth bass
RVSLSB05FP5	FP	Mercury	0.42	4.7	175	ng/g	0.851	RVSLSB05	5	5	St Lawrence	Smallmouth bass
RVSLSB06FP1	FP	Mercury	0.39	4.3	119	ng/g	0.923	RVSLSB06	6	1	St Lawrence	Smallmouth bass
RVSLSB06FP2	FP	Mercury	0.42	4.7	132	ng/g	0.853	RVSLSB06	6	2	St Lawrence	Smallmouth bass
RVSLSB06FP3	FP	Mercury	0.43	4.8	121	ng/g	0.838	RVSLSB06	6	3	St Lawrence	Smallmouth bass
RVSLSB06FP4	FP	Mercury	0.37	4.1	138	ng/g	0.976	RVSLSB06	6	4	St Lawrence	Smallmouth bass
RVSLSB06FP5	FP	Mercury	0.32	3.5	123	ng/g	1.127	RVSLSB06	6	5	St Lawrence	Smallmouth bass

Sample ID	Sample Type	Analyte	MDL	QL	Result (ng/g)	Units	Sample Weight (g)	Site-Specimen ID	Specimen Replicate Number	Sample Replicate Number	Water Body Name	Species
RVSLSB07FP1	FP	Mercury	0.35	3.9	274	ng/g	1.017	RVSLSB07	7	1	St Lawrence	Smallmouth bass
RVSLSB07FP2	FP	Mercury	0.51	5.6	228	ng/g	0.710	RVSLSB07	7	2	St Lawrence	Smallmouth bass
RVSLSB07FP3	FP	Mercury	0.45	5	256	ng/g	0.806	RVSLSB07	7	3	St Lawrence	Smallmouth bass
RVSLSB07FP4	FP	Mercury	0.44	4.9	229	ng/g	0.818	RVSLSB07	7	4	St Lawrence	Smallmouth bass
RVSLSB07FP5	FP	Mercury	0.45	5	271	ng/g	0.801	RVSLSB07	7	5	St Lawrence	Smallmouth bass
RVSLSB08FP1	FP	Mercury	0.43	4.7	174	ng/g	0.844	RVSLSB08	8	1	St Lawrence	Smallmouth bass
RVSLSB08FP2	FP	Mercury	0.41	4.6	201	ng/g	0.872	RVSLSB08	8	2	St Lawrence	Smallmouth bass
RVSLSB08FP3	FP	Mercury	0.38	4.2	179	ng/g	0.959	RVSLSB08	8	3	St Lawrence	Smallmouth bass
RVSLSB08FP4	FP	Mercury	0.43	4.7	198	ng/g	0.844	RVSLSB08	8	4	St Lawrence	Smallmouth bass
RVSLSB08FP5	FP	Mercury	0.38	4.2	183	ng/g	0.949	RVSLSB08	8	5	St Lawrence	Smallmouth bass
RVSLSB09FP1	FP	Mercury	0.37	4.1	168	ng/g	0.977	RVSLSB09	9	1	St Lawrence	Smallmouth bass
RVSLSB09FP2	FP	Mercury	0.36	4	187	ng/g	0.994	RVSLSB09	9	2	St Lawrence	Smallmouth bass
RVSLSB09FP3	FP	Mercury	0.42	4.7	160	ng/g	0.850	RVSLSB09	9	3	St Lawrence	Smallmouth bass
RVSLSB09FP4	FP	Mercury	0.32	3.6	190	ng/g	1.108	RVSLSB09	9	4	St Lawrence	Smallmouth bass
RVSLSB09FP5	FP	Mercury	0.29	3.2	168	ng/g	1.244	RVSLSB09	9	5	St Lawrence	Smallmouth bass
RVSLSB10FP1	FP	Mercury	0.33	3.6	213	ng/g	1.101	RVSLSB10	10	1	St Lawrence	Smallmouth bass
RVSLSB10FP2	FP	Mercury	0.36	4	184	ng/g	1.009	RVSLSB10	10	2	St Lawrence	Smallmouth bass
RVSLSB10FP3	FP	Mercury	0.37	4.1	208	ng/g	0.976	RVSLSB10	10	3	St Lawrence	Smallmouth bass
RVSLSB10FP4	FP	Mercury	0.39	4.3	188	ng/g	0.920	RVSLSB10	10	4	St Lawrence	Smallmouth bass
RVSLSB10FP5	FP	Mercury	0.41	4.5	207	ng/g	0.888	RVSLSB10	10	5	St Lawrence	Smallmouth bass
GLERWA01HF1	HF	Mercury	0.35	3.9	146	ng/g	1.023	GLERWA01	1	1	Erie	Walleye
GLERWA01HF2	HF	Mercury	0.35	3.9	135	ng/g	1.039	GLERWA01	1	2	Erie	Walleye
GLERWA01HF3	HF	Mercury	0.34	3.8	157	ng/g	1.064	GLERWA01	1	3	Erie	Walleye
GLERWA01HF4	HF	Mercury	0.35	3.9	142	ng/g	1.032	GLERWA01	1	4	Erie	Walleye
GLERWA01HF5	HF	Mercury	0.35	3.9	159	ng/g	1.018	GLERWA01	1	5	Erie	Walleye
GLERWA02HF1	HF	Mercury	0.36	3.9	93.2	ng/g	1.014	GLERWA02	2	1	Erie	Walleye
GLERWA02HF2	HF	Mercury	0.35	3.9	87.5	ng/g	1.030	GLERWA02	2	2	Erie	Walleye
GLERWA02HF3	HF	Mercury	0.36	4	96.8	ng/g	1.008	GLERWA02	2	3	Erie	Walleye
GLERWA02HF4	HF	Mercury	0.36	4	90	ng/g	1.011	GLERWA02	2	4	Erie	Walleye
GLERWA02HF5	HF	Mercury	0.35	3.9	95.8	ng/g	1.016	GLERWA02	2	5	Erie	Walleye
GLERWA03HF1	HF	Mercury	0.33	3.7	101	ng/g	1.084	GLERWA03	3	1	Erie	Walleye
GLERWA03HF2	HF	Mercury	0.33	3.6	101	ng/g	1.104	GLERWA03	3	2	Erie	Walleye
GLERWA03HF3	HF	Mercury	0.33	3.7	91	ng/g	1.091	GLERWA03	3	3	Erie	Walleye
GLERWA03HF4	HF	Mercury	0.31	3.4	101	ng/g	1.163	GLERWA03	3	4	Erie	Walleye
GLERWA03HF5	HF	Mercury	0.34	3.8	91.1	ng/g	1.060	GLERWA03	3	5	Erie	Walleye
GLERWA04HF1	HF	Mercury	0.33	3.7	121	ng/g	1.052	GLERWA04	4	1	Erie	Walleye
GLERWA04HF2	HF	Mercury	0.36	4	112	ng/g	1.009	GLERWA04	4	2	Erie	Walleye
GLERWA04HF3	HF	Mercury	0.31	3.4	122	ng/g	1.167	GLERWA04	4	3	Erie	Walleye
GLERWA04HF4	HF	Mercury	0.32	3.6	111	ng/g	1.112	GLERWA04	4	4	Erie	Walleye
GLERWA04HF5	HF	Mercury	0.33	3.7	121	ng/g	1.083	GLERWA04	4	5	Erie	Walleye

Sample ID	Sample Type	Analyte	MDL	QL	Result (ng/g)	Units	Sample Weight (g)	Site-Specimen ID	Specimen Replicate Number	Sample Replicate Number	Water Body Name	Species
GLERWA05HF1	HF	Mercury	0.35	3.9	212	ng/g	1.022	GLERWA05	5	1	Erie	Walleye
GLERWA05HF2	HF	Mercury	0.35	3.9	219	ng/g	1.033	GLERWA05	5	2	Erie	Walleye
GLERWA05HF3	HF	Mercury	0.35	3.9	194	ng/g	1.027	GLERWA05	5	3	Erie	Walleye
GLERWA05HF4	HF	Mercury	0.36	4	207	ng/g	1.004	GLERWA05	5	4	Erie	Walleye
GLERWA05HF5	HF	Mercury	0.35	3.9	195	ng/g	1.028	GLERWA05	5	5	Erie	Walleye
GLERWA06HF1	HF	Mercury	0.34	3.8	554	ng/g	1.046	GLERWA06	6	1	Erie	Walleye
GLERWA06HF2	HF	Mercury	0.35	3.8	519	ng/g	1.040	GLERWA06	6	2	Erie	Walleye
GLERWA06HF3	HF	Mercury	0.34	3.8	556	ng/g	1.048	GLERWA06	6	3	Erie	Walleye
GLERWA06HF4	HF	Mercury	0.35	3.9	497	ng/g	1.034	GLERWA06	6	4	Erie	Walleye
GLERWA06HF5	HF	Mercury	0.33	3.7	536	ng/g	1.079	GLERWA06	6	5	Erie	Walleye
GLERWA07HF1	HF	Mercury	0.35	3.9	108	ng/g	1.038	GLERWA07	7	1	Erie	Walleye
GLERWA07HF2	HF	Mercury	0.35	3.9	109	ng/g	1.011	GLERWA07	7	2	Erie	Walleye
GLERWA07HF3	HF	Mercury	0.35	3.9	97.1	ng/g	1.033	GLERWA07	7	3	Erie	Walleye
GLERWA07HF4	HF	Mercury	0.36	4	109	ng/g	1.021	GLERWA07	7	4	Erie	Walleye
GLERWA07HF5	HF	Mercury	0.36	4	98.5	ng/g	1.006	GLERWA07	7	5	Erie	Walleye
GLERWA08HF1	HF	Mercury	0.34	3.7	162	ng/g	1.023	GLERWA08	8	1	Erie	Walleye
GLERWA08HF2	HF	Mercury	0.34	3.8	144	ng/g	1.051	GLERWA08	8	2	Erie	Walleye
GLERWA08HF3	HF	Mercury	0.34	3.7	163	ng/g	1.068	GLERWA08	8	3	Erie	Walleye
GLERWA08HF4	HF	Mercury	0.34	3.8	150	ng/g	1.054	GLERWA08	8	4	Erie	Walleye
GLERWA08HF5	HF	Mercury	0.33	3.7	163	ng/g	1.091	GLERWA08	8	5	Erie	Walleye
GLERWA09HF1	HF	Mercury	0.34	3.7	124	ng/g	1.067	GLERWA09	9	1	Erie	Walleye
GLERWA09HF2	HF	Mercury	0.34	3.8	125	ng/g	1.091	GLERWA09	9	2	Erie	Walleye
GLERWA09HF3	HF	Mercury	0.35	3.9	115	ng/g	1.056	GLERWA09	9	3	Erie	Walleye
GLERWA09HF4	HF	Mercury	0.33	3.7	123	ng/g	1.084	GLERWA09	9	4	Erie	Walleye
GLERWA09HF5	HF	Mercury	0.34	3.7	114	ng/g	1.068	GLERWA09	9	5	Erie	Walleye
GLERWA10HF1	HF	Mercury	0.33	3.7	323	ng/g	1.081	GLERWA10	10	1	Erie	Walleye
GLERWA10HF2	HF	Mercury	0.36	4	307	ng/g	1.005	GLERWA10	10	2	Erie	Walleye
GLERWA10HF3	HF	Mercury	0.35	3.9	341	ng/g	1.034	GLERWA10	10	3	Erie	Walleye
GLERWA10HF4	HF	Mercury	0.36	4	303	ng/g	1.008	GLERWA10	10	4	Erie	Walleye
GLERWA10HF5	HF	Mercury	0.35	3.9	323	ng/g	1.017	GLERWA10	10	5	Erie	Walleye
GLMILT01HF1	HF	Mercury	0.34	3.8	98.6	ng/g	1.047	GLMILT01	1	1	Michigan	Lake trout
GLMILT01HF2	HF	Mercury	0.35	3.9	95.2	ng/g	1.021	GLMILT01	1	2	Michigan	Lake trout
GLMILT01HF3	HF	Mercury	0.36	3.9	108	ng/g	1.013	GLMILT01	1	3	Michigan	Lake trout
GLMILT01HF4	HF	Mercury	0.34	3.8	96.4	ng/g	1.060	GLMILT01	1	4	Michigan	Lake trout
GLMILT01HF5	HF	Mercury	0.34	3.8	106	ng/g	1.047	GLMILT01	1	5	Michigan	Lake trout
GLMILT02HF1	HF	Mercury	0.34	3.8	180	ng/g	1.055	GLMILT02	2	1	Michigan	Lake trout
GLMILT02HF2	HF	Mercury	0.35	3.9	184	ng/g	1.033	GLMILT02	2	2	Michigan	Lake trout
GLMILT02HF3	HF	Mercury	0.34	3.8	174	ng/g	1.062	GLMILT02	2	3	Michigan	Lake trout
GLMILT02HF4	HF	Mercury	0.33	3.7	179	ng/g	1.085	GLMILT02	2	4	Michigan	Lake trout
GLMILT02HF5	HF	Mercury	0.34	3.7	169	ng/g	1.074	GLMILT02	2	5	Michigan	Lake trout

Sample ID	Sample Type	Analyte	MDL	QL	Result (ng/g)	Units	Sample Weight (g)	Site-Specimen ID	Specimen Replicate Number	Sample Replicate Number	Water Body Name	Species
GLMILT03HF1	HF	Mercury	0.34	3.7	109	ng/g	1.067	GLMILT03	3	1	Michigan	Lake trout
GLMILT03HF2	HF	Mercury	0.34	3.8	113	ng/g	1.056	GLMILT03	3	2	Michigan	Lake trout
GLMILT03HF3	HF	Mercury	0.34	3.8	101	ng/g	1.038	GLMILT03	3	3	Michigan	Lake trout
GLMILT03HF4	HF	Mercury	0.35	3.8	110	ng/g	1.088	GLMILT03	3	4	Michigan	Lake trout
GLMILT03HF5	HF	Mercury	0.34	3.8	102	ng/g	1.078	GLMILT03	3	5	Michigan	Lake trout
GLMILT04HF1	HF	Mercury	0.35	3.9	187	ng/g	1.020	GLMILT04	4	1	Michigan	Lake trout
GLMILT04HF2	HF	Mercury	0.36	4	169	ng/g	1.067	GLMILT04	4	2	Michigan	Lake trout
GLMILT04HF3	HF	Mercury	0.35	3.9	183	ng/g	1.085	GLMILT04	4	3	Michigan	Lake trout
GLMILT04HF4	HF	Mercury	0.34	3.7	180	ng/g	1.044	GLMILT04	4	4	Michigan	Lake trout
GLMILT04HF5	HF	Mercury	0.34	3.8	183	ng/g	1.034	GLMILT04	4	5	Michigan	Lake trout
GLMILT05HF1	HF	Mercury	0.35	3.8	202	ng/g	1.043	GLMILT05	5	1	Michigan	Lake trout
GLMILT05HF2	HF	Mercury	0.33	3.7	191	ng/g	1.085	GLMILT05	5	2	Michigan	Lake trout
GLMILT05HF3	HF	Mercury	0.35	3.8	171	ng/g	1.041	GLMILT05	5	3	Michigan	Lake trout
GLMILT05HF4	HF	Mercury	0.33	3.7	197	ng/g	1.089	GLMILT05	5	4	Michigan	Lake trout
GLMILT05HF5	HF	Mercury	0.33	3.7	186	ng/g	1.083	GLMILT05	5	5	Michigan	Lake trout
GLMILT06HF1	HF	Mercury	0.35	3.9	163	ng/g	1.015	GLMILT06	6	1	Michigan	Lake trout
GLMILT06HF2	HF	Mercury	0.36	4	154	ng/g	1.005	GLMILT06	6	2	Michigan	Lake trout
GLMILT06HF3	HF	Mercury	0.33	3.7	159	ng/g	1.090	GLMILT06	6	3	Michigan	Lake trout
GLMILT06HF4	HF	Mercury	0.33	3.7	156	ng/g	1.090	GLMILT06	6	4	Michigan	Lake trout
GLMILT06HF5	HF	Mercury	0.33	3.7	160	ng/g	1.085	GLMILT06	6	5	Michigan	Lake trout
GLMILT07HF1	HF	Mercury	0.34	3.8	102	ng/g	1.055	GLMILT07	7	1	Michigan	Lake trout
GLMILT07HF2	HF	Mercury	0.35	3.8	103	ng/g	1.040	GLMILT07	7	2	Michigan	Lake trout
GLMILT07HF3	HF	Mercury	0.34	3.8	96.2	ng/g	1.058	GLMILT07	7	3	Michigan	Lake trout
GLMILT07HF4	HF	Mercury	0.35	3.9	108	ng/g	1.037	GLMILT07	7	4	Michigan	Lake trout
GLMILT07HF5	HF	Mercury	0.34	3.8	97.5	ng/g	1.044	GLMILT07	7	5	Michigan	Lake trout
GLMILT08HF1	HF	Mercury	0.35	3.9	104	ng/g	1.029	GLMILT08	8	1	Michigan	Lake trout
GLMILT08HF2	HF	Mercury	0.33	3.7	95.8	ng/g	1.079	GLMILT08	8	2	Michigan	Lake trout
GLMILT08HF3	HF	Mercury	0.33	3.7	104	ng/g	1.077	GLMILT08	8	3	Michigan	Lake trout
GLMILT08HF4	HF	Mercury	0.36	4	97.1	ng/g	1.011	GLMILT08	8	4	Michigan	Lake trout
GLMILT08HF5	HF	Mercury	0.34	3.8	102	ng/g	1.063	GLMILT08	8	5	Michigan	Lake trout
GLMILT09HF1	HF	Mercury	0.36	3.9	149	ng/g	1.055	GLMILT09	9	1	Michigan	Lake trout
GLMILT09HF2	HF	Mercury	0.35	3.9	153	ng/g	1.040	GLMILT09	9	2	Michigan	Lake trout
GLMILT09HF3	HF	Mercury	0.35	3.9	136	ng/g	1.058	GLMILT09	9	3	Michigan	Lake trout
GLMILT09HF4	HF	Mercury	0.36	3.9	156	ng/g	1.037	GLMILT09	9	4	Michigan	Lake trout
GLMILT09HF5	HF	Mercury	0.34	3.8	141	ng/g	1.044	GLMILT09	9	5	Michigan	Lake trout
GLMILT10HF1	HF	Mercury	0.34	3.8	222	ng/g	1.029	GLMILT10	10	1	Michigan	Lake trout
GLMILT10HF2	HF	Mercury	0.34	3.8	215	ng/g	1.079	GLMILT10	10	2	Michigan	Lake trout
GLMILT10HF3	HF	Mercury	0.33	3.7	220	ng/g	1.077	GLMILT10	10	3	Michigan	Lake trout
GLMILT10HF4	HF	Mercury	0.33	3.6	214	ng/g	1.011	GLMILT10	10	4	Michigan	Lake trout
GLMILT10HF5	HF	Mercury	0.34	3.8	221	ng/g	1.063	GLMILT10	10	5	Michigan	Lake trout

Sample ID	Sample Type	Analyte	MDL	QL	Result (ng/g)	Units	Sample Weight (g)	Site-Specimen ID	Specimen Replicate Number	Sample Replicate Number	Water Body Name	Species
GLONSA01HF1	HF	Mercury	0.35	3.8	154	ng/g	1.067	GLONSA01	1	1	Ontario	Chinook salmon
GLONSA01HF2	HF	Mercury	0.33	3.7	146	ng/g	1.056	GLONSA01	1	2	Ontario	Chinook salmon
GLONSA01HF3	HF	Mercury	0.35	3.9	140	ng/g	1.016	GLONSA01	1	3	Ontario	Chinook salmon
GLONSA01HF4	HF	Mercury	0.35	3.9	148	ng/g	1.084	GLONSA01	1	4	Ontario	Chinook salmon
GLONSA01HF5	HF	Mercury	0.36	4	135	ng/g	1.068	GLONSA01	1	5	Ontario	Chinook salmon
GLONSA02HF1	HF	Mercury	0.36	4	131	ng/g	1.081	GLONSA02	2	1	Ontario	Chinook salmon
GLONSA02HF2	HF	Mercury	0.35	3.8	119	ng/g	1.005	GLONSA02	2	2	Ontario	Chinook salmon
GLONSA02HF3	HF	Mercury	0.33	3.6	132	ng/g	1.034	GLONSA02	2	3	Ontario	Chinook salmon
GLONSA02HF4	HF	Mercury	0.34	3.8	123	ng/g	1.008	GLONSA02	2	4	Ontario	Chinook salmon
GLONSA02HF5	HF	Mercury	0.35	3.9	143	ng/g	1.017	GLONSA02	2	5	Ontario	Chinook salmon
GLONSA03HF1	HF	Mercury	0.35	3.9	153	ng/g	1.025	GLONSA03	3	1	Ontario	Chinook salmon
GLONSA03HF2	HF	Mercury	0.36	4	140	ng/g	1.005	GLONSA03	3	2	Ontario	Chinook salmon
GLONSA03HF3	HF	Mercury	0.34	3.8	142	ng/g	1.007	GLONSA03	3	3	Ontario	Chinook salmon
GLONSA03HF4	HF	Mercury	0.35	3.9	150	ng/g	1.065	GLONSA03	3	4	Ontario	Chinook salmon
GLONSA03HF5	HF	Mercury	0.34	3.7	135	ng/g	1.061	GLONSA03	3	5	Ontario	Chinook salmon
GLONSA04HF1	HF	Mercury	0.33	3.7	151	ng/g	0.940	GLONSA04	4	1	Ontario	Chinook salmon
GLONSA04HF2	HF	Mercury	0.34	3.8	139	ng/g	1.078	GLONSA04	4	2	Ontario	Chinook salmon
GLONSA04HF3	HF	Mercury	0.36	4	153	ng/g	1.015	GLONSA04	4	3	Ontario	Chinook salmon
GLONSA04HF4	HF	Mercury	0.35	3.9	135	ng/g	1.005	GLONSA04	4	4	Ontario	Chinook salmon
GLONSA04HF5	HF	Mercury	0.35	3.8	157	ng/g	1.031	GLONSA04	4	5	Ontario	Chinook salmon
GLONSA05HF1	HF	Mercury	0.34	3.8	165	ng/g	1.061	GLONSA05	5	1	Ontario	Chinook salmon
GLONSA05HF2	HF	Mercury	0.34	3.8	166	ng/g	1.063	GLONSA05	5	2	Ontario	Chinook salmon
GLONSA05HF3	HF	Mercury	0.35	3.9	155	ng/g	1.038	GLONSA05	5	3	Ontario	Chinook salmon
GLONSA05HF4	HF	Mercury	0.36	4	172	ng/g	1.005	GLONSA05	5	4	Ontario	Chinook salmon
GLONSA05HF5	HF	Mercury	0.33	3.7	149	ng/g	1.091	GLONSA05	5	5	Ontario	Chinook salmon
GLONSA06HF1	HF	Mercury	0.33	3.7	154	ng/g	1.092	GLONSA06	6	1	Ontario	Chinook salmon
GLONSA06HF2	HF	Mercury	0.33	3.7	150	ng/g	1.076	GLONSA06	6	2	Ontario	Chinook salmon
GLONSA06HF3	HF	Mercury	0.34	3.7	144	ng/g	1.074	GLONSA06	6	3	Ontario	Chinook salmon
GLONSA06HF4	HF	Mercury	0.35	3.9	149	ng/g	1.032	GLONSA06	6	4	Ontario	Chinook salmon
GLONSA06HF5	HF	Mercury	0.35	3.9	142	ng/g	1.015	GLONSA06	6	5	Ontario	Chinook salmon
GLONSA07HF1	HF	Mercury	0.34	3.7	171	ng/g	1.069	GLONSA07	7	1	Ontario	Chinook salmon
GLONSA07HF2	HF	Mercury	0.35	3.9	170	ng/g	1.016	GLONSA07	7	2	Ontario	Chinook salmon
GLONSA07HF3	HF	Mercury	0.34	3.8	159	ng/g	1.065	GLONSA07	7	3	Ontario	Chinook salmon
GLONSA07HF4	HF	Mercury	0.35	3.9	168	ng/g	1.022	GLONSA07	7	4	Ontario	Chinook salmon
GLONSA07HF5	HF	Mercury	0.35	3.9	161	ng/g	1.022	GLONSA07	7	5	Ontario	Chinook salmon
GLONSA08HF1	HF	Mercury	0.35	3.9	139	ng/g	1.036	GLONSA08	8	1	Ontario	Chinook salmon
GLONSA08HF2	HF	Mercury	0.35	3.8	137	ng/g	1.043	GLONSA08	8	2	Ontario	Chinook salmon
GLONSA08HF3	HF	Mercury	0.36	4	149	ng/g	1.011	GLONSA08	8	3	Ontario	Chinook salmon
GLONSA08HF4	HF	Mercury	0.33	3.7	137	ng/g	1.077	GLONSA08	8	4	Ontario	Chinook salmon
GLONSA08HF5	HF	Mercury	0.33	3.7	145	ng/g	1.094	GLONSA08	8	5	Ontario	Chinook salmon

Sample ID	Sample Type	Analyte	MDL	QL	Result (ng/g)	Units	Sample Weight (g)	Site-Specimen ID	Specimen Replicate Number	Sample Replicate Number	Water Body Name	Species
GLONSA09HF1	HF	Mercury	0.36	4	163	ng/g	1.008	GLONSA09	9	1	Ontario	Chinook salmon
GLONSA09HF2	HF	Mercury	0.35	3.9	160	ng/g	1.028	GLONSA09	9	2	Ontario	Chinook salmon
GLONSA09HF3	HF	Mercury	0.35	3.9	128	ng/g	1.027	GLONSA09	9	3	Ontario	Chinook salmon
GLONSA09HF4	HF	Mercury	0.34	3.7	158	ng/g	1.071	GLONSA09	9	4	Ontario	Chinook salmon
GLONSA09HF5	HF	Mercury	0.34	3.7	152	ng/g	1.073	GLONSA09	9	5	Ontario	Chinook salmon
GLONSA10HF1	HF	Mercury	0.36	3.9	153	ng/g	1.014	GLONSA10	10	1	Ontario	Chinook salmon
GLONSA10HF2	HF	Mercury	0.36	3.9	133	ng/g	1.013	GLONSA10	10	2	Ontario	Chinook salmon
GLONSA10HF3	HF	Mercury	0.33	3.7	151	ng/g	1.077	GLONSA10	10	3	Ontario	Chinook salmon
GLONSA10HF4	HF	Mercury	0.36	4	138	ng/g	1.005	GLONSA10	10	4	Ontario	Chinook salmon
GLONSA10HF5	HF	Mercury	0.35	3.8	150	ng/g	1.043	GLONSA10	10	5	Ontario	Chinook salmon
RVANBC01HF1	HF	Mercury	0.35	3.9	153	ng/g	1.001	RVANBC01	1	1	Anacostia	Blue catfish
RVANBC01HF2	HF	Mercury	0.35	3.9	136	ng/g	1.049	RVANBC01	1	2	Anacostia	Blue catfish
RVANBC01HF3	HF	Mercury	0.34	3.8	145	ng/g	1.002	RVANBC01	1	3	Anacostia	Blue catfish
RVANBC01HF4	HF	Mercury	0.34	3.7	135	ng/g	1.030	RVANBC01	1	4	Anacostia	Blue catfish
RVANBC01HF5	HF	Mercury	0.34	3.8	135	ng/g	1.068	RVANBC01	1	5	Anacostia	Blue catfish
RVANBC02HF1	HF	Mercury	0.35	3.9	63.9	ng/g	1.007	RVANBC02	2	1	Anacostia	Blue catfish
RVANBC02HF2	HF	Mercury	0.35	3.9	66.7	ng/g	1.013	RVANBC02	2	2	Anacostia	Blue catfish
RVANBC02HF3	HF	Mercury	0.34	3.8	62.5	ng/g	1.070	RVANBC02	2	3	Anacostia	Blue catfish
RVANBC02HF4	HF	Mercury	0.33	3.7	63.7	ng/g	1.001	RVANBC02	2	4	Anacostia	Blue catfish
RVANBC02HF5	HF	Mercury	0.33	3.7	61.5	ng/g	1.098	RVANBC02	2	5	Anacostia	Blue catfish
RVANBC03HF1	HF	Mercury	0.36	4	84.9	ng/g	1.002	RVANBC03	3	1	Anacostia	Blue catfish
RVANBC03HF2	HF	Mercury	0.35	3.9	84.6	ng/g	1.022	RVANBC03	3	2	Anacostia	Blue catfish
RVANBC03HF3	HF	Mercury	0.34	3.8	79	ng/g	1.058	RVANBC03	3	3	Anacostia	Blue catfish
RVANBC03HF4	HF	Mercury	0.34	3.8	80.8	ng/g	1.048	RVANBC03	3	4	Anacostia	Blue catfish
RVANBC03HF5	HF	Mercury	0.36	4	81.8	ng/g	1.007	RVANBC03	3	5	Anacostia	Blue catfish
RVANBC04HF1	HF	Mercury	0.34	3.7	53.1	ng/g	1.067	RVANBC04	4	1	Anacostia	Blue catfish
RVANBC04HF2	HF	Mercury	0.34	3.8	48.2	ng/g	1.063	RVANBC04	4	2	Anacostia	Blue catfish
RVANBC04HF3	HF	Mercury	0.34	3.7	52.7	ng/g	1.067	RVANBC04	4	3	Anacostia	Blue catfish
RVANBC04HF4	HF	Mercury	0.35	3.9	49.5	ng/g	1.017	RVANBC04	4	4	Anacostia	Blue catfish
RVANBC04HF5	HF	Mercury	0.34	3.8	52.5	ng/g	1.055	RVANBC04	4	5	Anacostia	Blue catfish
RVANBC05HF1	HF	Mercury	0.35	3.9	97	ng/g	1.032	RVANBC05	5	1	Anacostia	Blue catfish
RVANBC05HF2	HF	Mercury	0.33	3.7	94.5	ng/g	1.076	RVANBC05	5	2	Anacostia	Blue catfish
RVANBC05HF3	HF	Mercury	0.36	3.9	99.2	ng/g	1.013	RVANBC05	5	3	Anacostia	Blue catfish
RVANBC05HF4	HF	Mercury	0.34	3.7	92.3	ng/g	1.074	RVANBC05	5	4	Anacostia	Blue catfish
RVANBC05HF5	HF	Mercury	0.33	3.7	88.1	ng/g	1.082	RVANBC05	5	5	Anacostia	Blue catfish
RVANBC06HF1	HF	Mercury	0.34	3.8	263	ng/g	1.052	RVANBC06	6	1	Anacostia	Blue catfish
RVANBC06HF2	HF	Mercury	0.33	3.7	276	ng/g	1.082	RVANBC06	6	2	Anacostia	Blue catfish
RVANBC06HF3	HF	Mercury	0.34	3.8	252	ng/g	1.063	RVANBC06	6	3	Anacostia	Blue catfish
RVANBC06HF4	HF	Mercury	0.34	3.8	284	ng/g	1.044	RVANBC06	6	4	Anacostia	Blue catfish
RVANBC06HF5	HF	Mercury	0.35	3.9	262	ng/g	1.024	RVANBC06	6	5	Anacostia	Blue catfish

Sample ID	Sample Type	Analyte	MDL	QL	Result (ng/g)	Units	Sample Weight (g)	Site-Specimen ID	Specimen Replicate Number	Sample Replicate Number	Water Body Name	Species
RVANBC07HF1	HF	Mercury	0.34	3.8	75.5	ng/g	1.055	RVANBC07	7	1	Anacostia	Blue catfish
RVANBC07HF2	HF	Mercury	0.33	3.7	72.9	ng/g	1.083	RVANBC07	7	2	Anacostia	Blue catfish
RVANBC07HF3	HF	Mercury	0.33	3.7	69.6	ng/g	1.088	RVANBC07	7	3	Anacostia	Blue catfish
RVANBC07HF4	HF	Mercury	0.35	3.8	76.3	ng/g	1.043	RVANBC07	7	4	Anacostia	Blue catfish
RVANBC07HF5	HF	Mercury	0.35	3.9	69.4	ng/g	1.037	RVANBC07	7	5	Anacostia	Blue catfish
RVANBC08HF1	HF	Mercury	0.33	3.7	106	ng/g	1.092	RVANBC08	8	1	Anacostia	Blue catfish
RVANBC08HF2	HF	Mercury	0.34	3.8	92.7	ng/g	1.055	RVANBC08	8	2	Anacostia	Blue catfish
RVANBC08HF3	HF	Mercury	0.35	3.9	100	ng/g	1.037	RVANBC08	8	3	Anacostia	Blue catfish
RVANBC08HF4	HF	Mercury	0.35	3.9	96	ng/g	1.023	RVANBC08	8	4	Anacostia	Blue catfish
RVANBC08HF5	HF	Mercury	0.33	3.7	98.2	ng/g	1.090	RVANBC08	8	5	Anacostia	Blue catfish
RVANBC09HF1	HF	Mercury	0.36	4	76.5	ng/g	1.005	RVANBC09	9	1	Anacostia	Blue catfish
RVANBC09HF2	HF	Mercury	0.35	3.9	81.1	ng/g	1.020	RVANBC09	9	2	Anacostia	Blue catfish
RVANBC09HF3	HF	Mercury	0.34	3.7	76	ng/g	1.068	RVANBC09	9	3	Anacostia	Blue catfish
RVANBC09HF4	HF	Mercury	0.35	3.8	77	ng/g	1.042	RVANBC09	9	4	Anacostia	Blue catfish
RVANBC09HF5	HF	Mercury	0.36	4	23	ng/g	1.007	RVANBC09	9	5	Anacostia	Blue catfish
RVANBC10HF1	HF	Mercury	0.35	3.9	140	ng/g	1.027	RVANBC10	10	1	Anacostia	Blue catfish
RVANBC10HF2	HF	Mercury	0.36	4	126	ng/g	1.011	RVANBC10	10	2	Anacostia	Blue catfish
RVANBC10HF3	HF	Mercury	0.35	3.9	134	ng/g	1.036	RVANBC10	10	3	Anacostia	Blue catfish
RVANBC10HF4	HF	Mercury	0.35	3.9	124	ng/g	1.023	RVANBC10	10	4	Anacostia	Blue catfish
RVANBC10HF5	HF	Mercury	0.34	3.7	132	ng/g	1.071	RVANBC10	10	5	Anacostia	Blue catfish
RVPTLB01HF1	HF	Mercury	0.34	3.7	213	ng/g	1.069	RVPTLB01	1	1	Potomac	Largemouth bass
RVPTLB01HF2	HF	Mercury	0.35	3.9	216	ng/g	1.029	RVPTLB01	1	2	Potomac	Largemouth bass
RVPTLB01HF3	HF	Mercury	0.35	3.9	205	ng/g	1.020	RVPTLB01	1	3	Potomac	Largemouth bass
RVPTLB01HF4	HF	Mercury	0.34	3.8	219	ng/g	1.057	RVPTLB01	1	4	Potomac	Largemouth bass
RVPTLB01HF5	HF	Mercury	0.36	4	205	ng/g	1.012	RVPTLB01	1	5	Potomac	Largemouth bass
RVPTLB02HF1	HF	Mercury	0.36	4	95.8	ng/g	1.009	RVPTLB02	2	1	Potomac	Largemouth bass
RVPTLB02HF2	HF	Mercury	0.36	4	91.7	ng/g	1.009	RVPTLB02	2	2	Potomac	Largemouth bass
RVPTLB02HF3	HF	Mercury	0.34	3.7	94.8	ng/g	1.071	RVPTLB02	2	3	Potomac	Largemouth bass
RVPTLB02HF4	HF	Mercury	0.36	4	86.8	ng/g	1.007	RVPTLB02	2	4	Potomac	Largemouth bass
RVPTLB02HF5	HF	Mercury	0.36	4	93.9	ng/g	1.009	RVPTLB02	2	5	Potomac	Largemouth bass
RVPTLB03HF1	HF	Mercury	0.36	4	158	ng/g	1.012	RVPTLB03	3	1	Potomac	Largemouth bass
RVPTLB03HF2	HF	Mercury	0.35	3.9	152	ng/g	1.031	RVPTLB03	3	2	Potomac	Largemouth bass
RVPTLB03HF3	HF	Mercury	0.34	3.8	145	ng/g	1.054	RVPTLB03	3	3	Potomac	Largemouth bass
RVPTLB03HF4	HF	Mercury	0.34	3.8	111	ng/g	1.045	RVPTLB03	3	4	Potomac	Largemouth bass
RVPTLB03HF5	HF	Mercury	0.34	3.8	143	ng/g	1.047	RVPTLB03	3	5	Potomac	Largemouth bass
RVPTLB04HF1	HF	Mercury	0.34	3.8	128	ng/g	1.056	RVPTLB04	4	1	Potomac	Largemouth bass
RVPTLB04HF2	HF	Mercury	0.35	3.9	117	ng/g	1.021	RVPTLB04	4	2	Potomac	Largemouth bass
RVPTLB04HF3	HF	Mercury	0.35	3.9	130	ng/g	1.026	RVPTLB04	4	3	Potomac	Largemouth bass
RVPTLB04HF4	HF	Mercury	0.35	3.9	118	ng/g	1.028	RVPTLB04	4	4	Potomac	Largemouth bass
RVPTLB04HF5	HF	Mercury	0.36	4	121	ng/g	1.008	RVPTLB04	4	5	Potomac	Largemouth bass

Sample ID	Sample Type	Analyte	MDL	QL	Result (ng/g)	Units	Sample Weight (g)	Site-Specimen ID	Specimen Replicate Number	Sample Replicate Number	Water Body Name	Species
RVPTLB05HF1	HF	Mercury	0.34	3.8	97.8	ng/g	1.054	RVPTLB05	5	1	Potomac	Largemouth bass
RVPTLB05HF2	HF	Mercury	0.35	3.9	96	ng/g	1.017	RVPTLB05	5	2	Potomac	Largemouth bass
RVPTLB05HF3	HF	Mercury	0.35	3.8	91.7	ng/g	1.039	RVPTLB05	5	3	Potomac	Largemouth bass
RVPTLB05HF4	HF	Mercury	0.35	3.9	95.9	ng/g	1.032	RVPTLB05	5	4	Potomac	Largemouth bass
RVPTLB05HF5	HF	Mercury	0.36	3.9	90.7	ng/g	1.013	RVPTLB05	5	5	Potomac	Largemouth bass
RVPTLB06HF1	HF	Mercury	0.34	3.8	120	ng/g	1.051	RVPTLB06	6	1	Potomac	Largemouth bass
RVPTLB06HF2	HF	Mercury	0.34	3.7	112	ng/g	1.069	RVPTLB06	6	2	Potomac	Largemouth bass
RVPTLB06HF3	HF	Mercury	0.35	3.9	118	ng/g	1.020	RVPTLB06	6	3	Potomac	Largemouth bass
RVPTLB06HF4	HF	Mercury	0.35	3.9	109	ng/g	1.022	RVPTLB06	6	4	Potomac	Largemouth bass
RVPTLB06HF5	HF	Mercury	0.33	3.6	122	ng/g	1.097	RVPTLB06	6	5	Potomac	Largemouth bass
RVPTLB07HF1	HF	Mercury	0.33	3.7	102	ng/g	1.080	RVPTLB07	7	1	Potomac	Largemouth bass
RVPTLB07HF2	HF	Mercury	0.34	3.8	102	ng/g	1.048	RVPTLB07	7	2	Potomac	Largemouth bass
RVPTLB07HF3	HF	Mercury	0.36	4	108	ng/g	1.008	RVPTLB07	7	3	Potomac	Largemouth bass
RVPTLB07HF4	HF	Mercury	0.34	3.8	100	ng/g	1.063	RVPTLB07	7	4	Potomac	Largemouth bass
RVPTLB07HF5	HF	Mercury	0.35	3.9	110	ng/g	1.035	RVPTLB07	7	5	Potomac	Largemouth bass
RVPTLB08HF1	HF	Mercury	0.35	3.9	446	ng/g	1.026	RVPTLB08	8	1	Potomac	Largemouth bass
RVPTLB08HF2	HF	Mercury	0.34	3.7	460	ng/g	1.068	RVPTLB08	8	2	Potomac	Largemouth bass
RVPTLB08HF3	HF	Mercury	0.35	3.9	430	ng/g	1.027	RVPTLB08	8	3	Potomac	Largemouth bass
RVPTLB08HF4	HF	Mercury	0.36	4	475	ng/g	1.008	RVPTLB08	8	4	Potomac	Largemouth bass
RVPTLB08HF5	HF	Mercury	0.34	3.8	449	ng/g	1.055	RVPTLB08	8	5	Potomac	Largemouth bass
RVPTLB09HF1	HF	Mercury	0.33	3.7	319	ng/g	1.083	RVPTLB09	9	1	Potomac	Largemouth bass
RVPTLB09HF2	HF	Mercury	0.35	3.9	326	ng/g	1.021	RVPTLB09	9	2	Potomac	Largemouth bass
RVPTLB09HF3	HF	Mercury	0.36	4	330	ng/g	1.005	RVPTLB09	9	3	Potomac	Largemouth bass
RVPTLB09HF4	HF	Mercury	0.35	3.9	323	ng/g	1.024	RVPTLB09	9	4	Potomac	Largemouth bass
RVPTLB09HF5	HF	Mercury	0.36	4	325	ng/g	1.010	RVPTLB09	9	5	Potomac	Largemouth bass
RVPTLB10HF1	HF	Mercury	0.35	3.9	102	ng/g	1.032	RVPTLB10	10	1	Potomac	Largemouth bass
RVPTLB10HF2	HF	Mercury	0.35	3.9	110	ng/g	1.036	RVPTLB10	10	2	Potomac	Largemouth bass
RVPTLB10HF3	HF	Mercury	0.34	3.8	107	ng/g	1.060	RVPTLB10	10	3	Potomac	Largemouth bass
RVPTLB10HF4	HF	Mercury	0.34	3.8	112	ng/g	1.056	RVPTLB10	10	4	Potomac	Largemouth bass
RVPTLB10HF5	HF	Mercury	0.36	4	106	ng/g	1.007	RVPTLB10	10	5	Potomac	Largemouth bass
RVSLSB01HF1	HF	Mercury	0.36	4	189	ng/g	1.040	RVSLSB01	1	1	St Lawrence	Smallmouth bass
RVSLSB01HF2	HF	Mercury	0.35	3.9	191	ng/g	1.019	RVSLSB01	1	2	St Lawrence	Smallmouth bass
RVSLSB01HF3	HF	Mercury	0.34	3.7	183	ng/g	1.071	RVSLSB01	1	3	St Lawrence	Smallmouth bass
RVSLSB01HF4	HF	Mercury	0.35	3.8	187	ng/g	1.030	RVSLSB01	1	4	St Lawrence	Smallmouth bass
RVSLSB01HF5	HF	Mercury	0.36	4	187	ng/g	1.043	RVSLSB01	1	5	St Lawrence	Smallmouth bass
RVSLSB02HF1	HF	Mercury	0.35	3.9	250	ng/g	1.083	RVSLSB02	2	1	St Lawrence	Smallmouth bass
RVSLSB02HF2	HF	Mercury	0.36	4	241	ng/g	1.008	RVSLSB02	2	2	St Lawrence	Smallmouth bass
RVSLSB02HF3	HF	Mercury	0.35	3.9	247	ng/g	1.002	RVSLSB02	2	3	St Lawrence	Smallmouth bass
RVSLSB02HF4	HF	Mercury	0.35	3.9	237	ng/g	1.038	RVSLSB02	2	4	St Lawrence	Smallmouth bass
RVSLSB02HF5	HF	Mercury	0.34	3.7	244	ng/g	1.060	RVSLSB02	2	5	St Lawrence	Smallmouth bass

Sample ID	Sample Type	Analyte	MDL	QL	Result (ng/g)	Units	Sample Weight (g)	Site-Specimen ID	Specimen Replicate Number	Sample Replicate Number	Water Body Name	Species
RVSLSB03HF1	HF	Mercury	0.35	3.9	105	ng/g	1.022	RVSLSB03	3	1	St Lawrence	Smallmouth bass
RVSLSB03HF2	HF	Mercury	0.35	3.9	108	ng/g	1.033	RVSLSB03	3	2	St Lawrence	Smallmouth bass
RVSLSB03HF3	HF	Mercury	0.33	3.7	113	ng/g	1.085	RVSLSB03	3	3	St Lawrence	Smallmouth bass
RVSLSB03HF4	HF	Mercury	0.35	3.9	105	ng/g	1.025	RVSLSB03	3	4	St Lawrence	Smallmouth bass
RVSLSB03HF5	HF	Mercury	0.36	4	114	ng/g	1.004	RVSLSB03	3	5	St Lawrence	Smallmouth bass
RVSLSB04HF1	HF	Mercury	0.33	3.6	287	ng/g	1.098	RVSLSB04	4	1	St Lawrence	Smallmouth bass
RVSLSB04HF2	HF	Mercury	0.36	4	315	ng/g	1.011	RVSLSB04	4	2	St Lawrence	Smallmouth bass
RVSLSB04HF3	HF	Mercury	0.34	3.8	297	ng/g	1.048	RVSLSB04	4	3	St Lawrence	Smallmouth bass
RVSLSB04HF4	HF	Mercury	0.36	4	317	ng/g	1.006	RVSLSB04	4	4	St Lawrence	Smallmouth bass
RVSLSB04HF5	HF	Mercury	0.36	4	303	ng/g	1.012	RVSLSB04	4	5	St Lawrence	Smallmouth bass
RVSLSB05HF1	HF	Mercury	0.36	4	185	ng/g	1.007	RVSLSB05	5	1	St Lawrence	Smallmouth bass
RVSLSB05HF2	HF	Mercury	0.35	3.8	185	ng/g	1.039	RVSLSB05	5	2	St Lawrence	Smallmouth bass
RVSLSB05HF3	HF	Mercury	0.35	3.9	169	ng/g	1.026	RVSLSB05	5	3	St Lawrence	Smallmouth bass
RVSLSB05HF4	HF	Mercury	0.35	3.8	184	ng/g	1.040	RVSLSB05	5	4	St Lawrence	Smallmouth bass
RVSLSB05HF5	HF	Mercury	0.34	3.8	173	ng/g	1.045	RVSLSB05	5	5	St Lawrence	Smallmouth bass
RVSLSB06HF1	HF	Mercury	0.35	3.9	141	ng/g	1.035	RVSLSB06	6	1	St Lawrence	Smallmouth bass
RVSLSB06HF2	HF	Mercury	0.35	3.9	132	ng/g	1.018	RVSLSB06	6	2	St Lawrence	Smallmouth bass
RVSLSB06HF3	HF	Mercury	0.34	3.8	146	ng/g	1.060	RVSLSB06	6	3	St Lawrence	Smallmouth bass
RVSLSB06HF4	HF	Mercury	0.36	4	135	ng/g	1.007	RVSLSB06	6	4	St Lawrence	Smallmouth bass
RVSLSB06HF5	HF	Mercury	0.33	3.7	144	ng/g	1.077	RVSLSB06	6	5	St Lawrence	Smallmouth bass
RVSLSB07HF1	HF	Mercury	0.35	3.9	263	ng/g	1.015	RVSLSB07	7	1	St Lawrence	Smallmouth bass
RVSLSB07HF2	HF	Mercury	0.36	4	262	ng/g	1.001	RVSLSB07	7	2	St Lawrence	Smallmouth bass
RVSLSB07HF3	HF	Mercury	0.33	3.7	239	ng/g	1.094	RVSLSB07	7	3	St Lawrence	Smallmouth bass
RVSLSB07HF4	HF	Mercury	0.34	3.8	253	ng/g	1.048	RVSLSB07	7	4	St Lawrence	Smallmouth bass
RVSLSB07HF5	HF	Mercury	0.35	3.8	254	ng/g	1.042	RVSLSB07	7	5	St Lawrence	Smallmouth bass
RVSLSB08HF1	HF	Mercury	0.36	4	208	ng/g	1.002	RVSLSB08	8	1	St Lawrence	Smallmouth bass
RVSLSB08HF2	HF	Mercury	0.34	3.8	194	ng/g	1.062	RVSLSB08	8	2	St Lawrence	Smallmouth bass
RVSLSB08HF3	HF	Mercury	0.35	3.8	210	ng/g	1.042	RVSLSB08	8	3	St Lawrence	Smallmouth bass
RVSLSB08HF4	HF	Mercury	0.34	3.8	188	ng/g	1.048	RVSLSB08	8	4	St Lawrence	Smallmouth bass
RVSLSB08HF5	HF	Mercury	0.33	3.7	207	ng/g	1.076	RVSLSB08	8	5	St Lawrence	Smallmouth bass
RVSLSB09HF1	HF	Mercury	0.35	3.9	173	ng/g	1.030	RVSLSB09	9	1	St Lawrence	Smallmouth bass
RVSLSB09HF2	HF	Mercury	0.35	3.9	175	ng/g	1.033	RVSLSB09	9	2	St Lawrence	Smallmouth bass
RVSLSB09HF3	HF	Mercury	0.35	3.8	185	ng/g	1.042	RVSLSB09	9	3	St Lawrence	Smallmouth bass
RVSLSB09HF4	HF	Mercury	0.34	3.7	169	ng/g	1.067	RVSLSB09	9	4	St Lawrence	Smallmouth bass
RVSLSB09HF5	HF	Mercury	0.36	4	186	ng/g	1.011	RVSLSB09	9	5	St Lawrence	Smallmouth bass
RVSLSB10HF1	HF	Mercury	0.34	3.8	192	ng/g	1.060	RVSLSB10	10	1	St Lawrence	Smallmouth bass
RVSLSB10HF2	HF	Mercury	0.36	4	214	ng/g	1.002	RVSLSB10	10	2	St Lawrence	Smallmouth bass
RVSLSB10HF3	HF	Mercury	0.34	3.8	197	ng/g	1.056	RVSLSB10	10	3	St Lawrence	Smallmouth bass
RVSLSB10HF4	HF	Mercury	0.35	3.8	208	ng/g	1.043	RVSLSB10	10	4	St Lawrence	Smallmouth bass
RVSLSB10HF5	HF	Mercury	0.33	3.7	194	ng/g	1.093	RVSLSB10	10	5	St Lawrence	Smallmouth bass

Supplementary Information

Table SI2. Fish Plug Evaluation Study fillet plug sample (FP), homogenized fillet sample (HF) selenium and total solids data

Sample ID	Sample Type	Analyte	MDL	QL	Selenium WW result	Selenium DW result	Se Results Units	Se Sample Weight (g)	Total Solids (%)	Site-Specimen ID	Specimen Replicate Number	Sample Replicate Number	Water Body Name	Species
GLERWA01FP1	FP	Selenium	41.7	126.0	455	2159	ng/g	1.222	21.07	GLERWA01	1	1	Erie	Walleye
GLERWA01FP2	FP	Selenium	37.7	114.0	448	1978	ng/g	1.351	22.65	GLERWA01	1	2	Erie	Walleye
GLERWA01FP3	FP	Selenium	39.0	118.1	445	2042	ng/g	1.304	21.79	GLERWA01	1	3	Erie	Walleye
GLERWA01FP4	FP	Selenium	36.5	110.3	453	1986	ng/g	1.396	22.81	GLERWA01	1	4	Erie	Walleye
GLERWA02FP1	FP	Selenium	38.6	116.7	459	2096	ng/g	1.320	21.90	GLERWA02	2	1	Erie	Walleye
GLERWA02FP2	FP	Selenium	46.4	140.4	467	2176	ng/g	1.097	21.46	GLERWA02	2	2	Erie	Walleye
GLERWA02FP3	FP	Selenium	36.6	110.6	448	2039	ng/g	1.392	21.97	GLERWA02	2	3	Erie	Walleye
GLERWA02FP4	FP	Selenium	46.1	139.6	446	2030	ng/g	1.103	21.97	GLERWA02	2	4	Erie	Walleye
GLERWA03FP1	FP	Selenium	47.7	144.5	436	1934	ng/g	1.066	22.54	GLERWA03	3	1	Erie	Walleye
GLERWA03FP2	FP	Selenium	54.7	165.6	438	1783	ng/g	0.930	24.56	GLERWA03	3	2	Erie	Walleye
GLERWA03FP3	FP	Selenium	60.6	183.3	469	2133	ng/g	0.840	21.99	GLERWA03	3	3	Erie	Walleye
GLERWA03FP4	FP	Selenium	54.3	164.4	436	1859	ng/g	0.937	23.45	GLERWA03	3	4	Erie	Walleye
GLERWA04FP1	FP	Selenium	51.7	156.5	390	1755	ng/g	0.984	22.22	GLERWA04	4	1	Erie	Walleye
GLERWA04FP2	FP	Selenium	32.1	97.2	380	1675	ng/g	1.584	22.68	GLERWA04	4	2	Erie	Walleye
GLERWA04FP3	FP	Selenium	40.1	121.5	360	1642	ng/g	1.268	21.92	GLERWA04	4	3	Erie	Walleye
GLERWA04FP4	FP	Selenium	34.2	103.4	367	1549	ng/g	1.490	23.70	GLERWA04	4	4	Erie	Walleye
GLERWA05FP1	FP	Selenium	32.4	97.9	384	1767	ng/g	1.573	21.73	GLERWA05	5	1	Erie	Walleye
GLERWA05FP2	FP	Selenium	47.3	143.1	396	1681	ng/g	1.076	23.56	GLERWA05	5	2	Erie	Walleye
GLERWA05FP3	FP	Selenium	47.0	142.1	395	1815	ng/g	1.084	21.76	GLERWA05	5	3	Erie	Walleye
GLERWA05FP4	FP	Selenium	52.7	159.4	374	1658	ng/g	0.966	22.56	GLERWA05	5	4	Erie	Walleye
GLMILT01FP1	FP	Selenium	43.2	130.7	636	1690	ng/g	1.178	37.64	GLMILT01	1	1	Michigan	Lake trout
GLMILT01FP2	FP	Selenium	41.2	124.7	616	1726	ng/g	1.235	35.68	GLMILT01	1	2	Michigan	Lake trout
GLMILT01FP3	FP	Selenium	45.6	137.9	599	1694	ng/g	1.117	35.35	GLMILT01	1	3	Michigan	Lake trout
GLMILT01FP4	FP	Selenium	40.1	121.5	604	1778	ng/g	1.268	33.98	GLMILT01	1	4	Michigan	Lake trout
GLMILT02FP1	FP	Selenium	40.7	123.0	736	1880	ng/g	1.252	39.15	GLMILT02	2	1	Michigan	Lake trout
GLMILT02FP2	FP	Selenium	39.2	118.6	741	1619	ng/g	1.299	45.76	GLMILT02	2	2	Michigan	Lake trout
GLMILT02FP3	FP	Selenium	33.7	102.0	722	2007	ng/g	1.510	35.98	GLMILT02	2	3	Michigan	Lake trout
GLMILT02FP4	FP	Selenium	40.8	123.4	700	1722	ng/g	1.248	40.66	GLMILT02	2	4	Michigan	Lake trout
GLMILT03FP1	FP	Selenium	39.5	119.5	640	1801	ng/g	1.289	35.54	GLMILT03	3	1	Michigan	Lake trout
GLMILT03FP2	FP	Selenium	51.6	156.2	619	1672	ng/g	0.986	37.03	GLMILT03	3	2	Michigan	Lake trout
GLMILT03FP3	FP	Selenium	48.5	146.8	608	1867	ng/g	1.049	32.56	GLMILT03	3	3	Michigan	Lake trout
GLMILT03FP4	FP	Selenium	50.2	152.0	623	1757	ng/g	1.013	35.45	GLMILT03	3	4	Michigan	Lake trout
GLMILT04FP1	FP	Selenium	49.7	150.2	753	1926	ng/g	1.025	39.10	GLMILT04	4	1	Michigan	Lake trout
GLMILT04FP2	FP	Selenium	61.8	187.1	715	2106	ng/g	0.823	33.95	GLMILT04	4	2	Michigan	Lake trout
GLMILT04FP3	FP	Selenium	65.1	196.9	757	2315	ng/g	0.782	32.70	GLMILT04	4	3	Michigan	Lake trout
GLMILT04FP4	FP	Selenium	64.9	196.4	744	2271	ng/g	0.784	32.76	GLMILT04	4	4	Michigan	Lake trout
GLMILT05FP1	FP	Selenium	45.7	138.2	796	2529	ng/g	1.114	31.47	GLMILT05	5	1	Michigan	Lake trout
GLMILT05FP2	FP	Selenium	35.9	108.8	823	2669	ng/g	1.416	30.83	GLMILT05	5	2	Michigan	Lake trout
GLMILT05FP3	FP	Selenium	37.3	112.8	792	2655	ng/g	1.365	29.83	GLMILT05	5	3	Michigan	Lake trout
GLMILT05FP4	FP	Selenium	39.2	118.5	802	2586	ng/g	1.300	31.01	GLMILT05	5	4	Michigan	Lake trout

Sample ID	Sample Type	Analyte	MDL	QL	Selenium WW result	Selenium DW result	Se Results Units	Se Sample Weight (g)	Total Solids (%)	Site-Specimen ID	Specimen Replicate Number	Sample Replicate Number	Water Body Name	Species
GLONSA01FP1	FP	Selenium	52.5	158.8	442	1561	ng/g	0.970	28.32	GLONSA01	1	1	Ontario	Chinook salmon
GLONSA01FP2	FP	Selenium	52.7	159.6	428	1547	ng/g	0.965	27.66	GLONSA01	1	2	Ontario	Chinook salmon
GLONSA01FP3	FP	Selenium	32.8	99.3	443	1635	ng/g	1.551	27.09	GLONSA01	1	3	Ontario	Chinook salmon
GLONSA01FP4	FP	Selenium	42.3	128.1	438	1542	ng/g	1.202	28.41	GLONSA01	1	4	Ontario	Chinook salmon
GLONSA02FP1	FP	Selenium	43.5	131.6	477	1925	ng/g	1.170	24.78	GLONSA02	2	1	Ontario	Chinook salmon
GLONSA02FP2	FP	Selenium	38.0	114.9	463	1697	ng/g	1.340	27.29	GLONSA02	2	2	Ontario	Chinook salmon
GLONSA02FP3	FP	Selenium	36.4	110.2	457	1835	ng/g	1.398	24.90	GLONSA02	2	3	Ontario	Chinook salmon
GLONSA02FP4	FP	Selenium	35.8	108.4	459	1704	ng/g	1.421	26.94	GLONSA02	2	4	Ontario	Chinook salmon
GLONSA03FP1	FP	Selenium	35.9	108.7	428	1599	ng/g	1.417	26.77	GLONSA03	3	1	Ontario	Chinook salmon
GLONSA03FP2	FP	Selenium	32.8	99.2	440	1628	ng/g	1.552	27.02	GLONSA03	3	2	Ontario	Chinook salmon
GLONSA03FP3	FP	Selenium	40.3	122.0	443	1633	ng/g	1.262	27.13	GLONSA03	3	3	Ontario	Chinook salmon
GLONSA03FP4	FP	Selenium	45.7	138.2	454	1732	ng/g	1.114	26.22	GLONSA03	3	4	Ontario	Chinook salmon
GLONSA04FP1	FP	Selenium	51.6	156.0	483	1856	ng/g	0.987	26.03	GLONSA04	4	1	Ontario	Chinook salmon
GLONSA04FP2	FP	Selenium	50.3	152.3	499	1790	ng/g	1.011	27.87	GLONSA04	4	2	Ontario	Chinook salmon
GLONSA04FP3	FP	Selenium	41.8	126.3	495	1598	ng/g	1.219	30.97	GLONSA04	4	3	Ontario	Chinook salmon
GLONSA04FP4	FP	Selenium	43.7	132.2	504	1810	ng/g	1.165	27.84	GLONSA04	4	4	Ontario	Chinook salmon
GLONSA05FP1	FP	Selenium	48.3	146.1	475	1438	ng/g	1.054	33.03	GLONSA05	5	1	Ontario	Chinook salmon
GLONSA05FP2	FP	Selenium	56.0	169.4	475	1430	ng/g	0.909	33.21	GLONSA05	5	2	Ontario	Chinook salmon
GLONSA05FP3	FP	Selenium	44.8	135.6	475	1427	ng/g	1.136	33.28	GLONSA05	5	3	Ontario	Chinook salmon
GLONSA05FP4	FP	Selenium	43.7	132.1	482	1505	ng/g	1.166	32.03	GLONSA05	5	4	Ontario	Chinook salmon
RVANBC01FP1	FP	Selenium	51.6	156.0	190	919	ng/g	0.987	20.67	RVANBC01	1	1	Anacostia	Blue catfish
RVANBC01FP2	FP	Selenium	70.6	213.6	234	1158	ng/g	0.721	20.20	RVANBC01	1	2	Anacostia	Blue catfish
RVANBC01FP3	FP	Selenium	75.4	228.1	203	993	ng/g	0.675	20.45	RVANBC01	1	3	Anacostia	Blue catfish
RVANBC01FP4	FP	Selenium	46.1	139.4	175	851	ng/g	1.105	20.56	RVANBC01	1	4	Anacostia	Blue catfish
RVANBC02FP1	FP	Selenium	70.2	212.4	161	892	ng/g	0.725	18.05	RVANBC02	2	1	Anacostia	Blue catfish
RVANBC02FP2	FP	Selenium	57.1	172.8	139	817	ng/g	0.891	17.01	RVANBC02	2	2	Anacostia	Blue catfish
RVANBC02FP3	FP	Selenium	68.0	205.6	174	958	ng/g	0.749	18.16	RVANBC02	2	3	Anacostia	Blue catfish
RVANBC02FP4	FP	Selenium	51.7	156.5	153	871	ng/g	0.984	17.57	RVANBC02	2	4	Anacostia	Blue catfish
RVANBC03FP1	FP	Selenium	46.8	141.5	151	760	ng/g	1.088	19.86	RVANBC03	3	1	Anacostia	Blue catfish
RVANBC03FP2	FP	Selenium	51.3	155.1	145	735	ng/g	0.993	19.74	RVANBC03	3	2	Anacostia	Blue catfish
RVANBC03FP3	FP	Selenium	62.2	188.3	153	771	ng/g	0.818	19.84	RVANBC03	3	3	Anacostia	Blue catfish
RVANBC03FP4	FP	Selenium	50.2	152.0	156	712	ng/g	1.013	21.91	RVANBC03	3	4	Anacostia	Blue catfish
RVANBC04FP1	FP	Selenium	93.7	283.6	178	867	ng/g	0.543	20.52	RVANBC04	4	1	Anacostia	Blue catfish
RVANBC04FP2	FP	Selenium	76.5	231.6	171	799	ng/g	0.665	21.41	RVANBC04	4	2	Anacostia	Blue catfish
RVANBC04FP3	FP	Selenium	55.3	167.4	174	837	ng/g	0.920	20.79	RVANBC04	4	3	Anacostia	Blue catfish
RVANBC04FP4	FP	Selenium	60.8	184.0	169	760	ng/g	0.837	22.24	RVANBC04	4	4	Anacostia	Blue catfish
RVANBC05FP1	FP	Selenium	54.9	166.1	180	812	ng/g	0.927	22.17	RVANBC05	5	1	Anacostia	Blue catfish
RVANBC05FP2	FP	Selenium	59.7	180.8	170	711	ng/g	0.852	23.91	RVANBC05	5	2	Anacostia	Blue catfish
RVANBC05FP3	FP	Selenium	61.8	187.1	186	906	ng/g	0.823	20.53	RVANBC05	5	3	Anacostia	Blue catfish
RVANBC05FP4	FP	Selenium	56.2	170.0	177	831	ng/g	0.906	21.30	RVANBC05	5	4	Anacostia	Blue catfish

Sample ID	Sample Type	Analyte	MDL	QL	Selenium WW result	Selenium DW result	Se Results Units	Se Sample Weight (g)	Total Solids (%)	Site-Specimen ID	Specimen Replicate Number	Sample Replicate Number	Water Body Name	Species
RVPTLB01FP1	FP	Selenium	49.9	151.0	277	1439	ng/g	1.020	19.25	RVPTLB01	1	1	Potomac	Largemouth bass
RVPTLB01FP2	FP	Selenium	57.6	174.4	272	1392	ng/g	0.883	19.54	RVPTLB01	1	2	Potomac	Largemouth bass
RVPTLB01FP3	FP	Selenium	44.0	133.0	264	1411	ng/g	1.158	18.71	RVPTLB01	1	3	Potomac	Largemouth bass
RVPTLB01FP4	FP	Selenium	44.1	133.4	264	1389	ng/g	1.154	19.00	RVPTLB01	1	4	Potomac	Largemouth bass
RVPTLB02FP1	FP	Selenium	52.9	160.1	321	1611	ng/g	0.962	19.93	RVPTLB02	2	1	Potomac	Largemouth bass
RVPTLB02FP2	FP	Selenium	52.3	158.1	315	1540	ng/g	0.974	20.46	RVPTLB02	2	2	Potomac	Largemouth bass
RVPTLB02FP3	FP	Selenium	42.6	128.9	314	1604	ng/g	1.195	19.58	RVPTLB02	2	3	Potomac	Largemouth bass
RVPTLB02FP4	FP	Selenium	49.6	150.1	321	1598	ng/g	1.026	20.09	RVPTLB02	2	4	Potomac	Largemouth bass
RVPTLB03FP1	FP	Selenium	37.6	113.9	376	1802	ng/g	1.352	20.86	RVPTLB03	3	1	Potomac	Largemouth bass
RVPTLB03FP2	FP	Selenium	37.4	113.2	377	1803	ng/g	1.361	20.91	RVPTLB03	3	2	Potomac	Largemouth bass
RVPTLB03FP3	FP	Selenium	37.8	114.3	375	1805	ng/g	1.347	20.78	RVPTLB03	3	3	Potomac	Largemouth bass
RVPTLB03FP4	FP	Selenium	35.7	108.1	377	1817	ng/g	1.424	20.75	RVPTLB03	3	4	Potomac	Largemouth bass
RVPTLB04FP1	FP	Selenium	33.5	101.3	416	1917	ng/g	1.520	21.70	RVPTLB04	4	1	Potomac	Largemouth bass
RVPTLB04FP2	FP	Selenium	38.2	115.4	434	1978	ng/g	1.334	21.94	RVPTLB04	4	2	Potomac	Largemouth bass
RVPTLB04FP3	FP	Selenium	33.5	101.2	422	1971	ng/g	1.521	21.41	RVPTLB04	4	3	Potomac	Largemouth bass
RVPTLB04FP4	FP	Selenium	39.6	119.9	418	1901	ng/g	1.284	21.99	RVPTLB04	4	4	Potomac	Largemouth bass
RVPTLB05FP1	FP	Selenium	54.8	165.8	348	1680	ng/g	0.929	20.71	RVPTLB05	5	1	Potomac	Largemouth bass
RVPTLB05FP2	FP	Selenium	53.9	163.0	338	1644	ng/g	0.945	20.56	RVPTLB05	5	2	Potomac	Largemouth bass
RVPTLB05FP3	FP	Selenium	45.0	136.2	324	1590	ng/g	1.131	20.38	RVPTLB05	5	3	Potomac	Largemouth bass
RVPTLB05FP4	FP	Selenium	57.9	175.2	342	1673	ng/g	0.879	20.44	RVPTLB05	5	4	Potomac	Largemouth bass
RVSLSB01FP1	FP	Selenium	29.5	89.3	755	3344	ng/g	1.725	22.58	RVSLSB01	1	1	St Lawrence	Smallmouth bass
RVSLSB01FP2	FP	Selenium	33.3	100.9	748	3276	ng/g	1.527	22.83	RVSLSB01	1	2	St Lawrence	Smallmouth bass
RVSLSB01FP3	FP	Selenium	41.4	125.4	758	3366	ng/g	1.228	22.52	RVSLSB01	1	3	St Lawrence	Smallmouth bass
RVSLSB01FP4	FP	Selenium	39.6	119.8	772	3389	ng/g	1.285	22.78	RVSLSB01	1	4	St Lawrence	Smallmouth bass
RVSLSB02FP1	FP	Selenium	43.9	132.8	848	3666	ng/g	1.160	23.13	RVSLSB02	2	1	St Lawrence	Smallmouth bass
RVSLSB02FP2	FP	Selenium	37.5	113.4	846	3531	ng/g	1.358	23.96	RVSLSB02	2	2	St Lawrence	Smallmouth bass
RVSLSB02FP3	FP	Selenium	47.0	142.1	846	3623	ng/g	1.084	23.35	RVSLSB02	2	3	St Lawrence	Smallmouth bass
RVSLSB02FP4	FP	Selenium	48.4	146.4	842	3440	ng/g	1.052	24.48	RVSLSB02	2	4	St Lawrence	Smallmouth bass
RVSLSB03FP1	FP	Selenium	47.0	142.2	765	3501	ng/g	1.083	21.85	RVSLSB03	3	1	St Lawrence	Smallmouth bass
RVSLSB03FP2	FP	Selenium	50.5	152.8	775	3542	ng/g	1.008	21.88	RVSLSB03	3	2	St Lawrence	Smallmouth bass
RVSLSB03FP3	FP	Selenium	53.2	160.9	807	3814	ng/g	0.957	21.16	RVSLSB03	3	3	St Lawrence	Smallmouth bass
RVSLSB03FP4	FP	Selenium	48.1	145.6	792	3442	ng/g	1.058	23.01	RVSLSB03	3	4	St Lawrence	Smallmouth bass
RVSLSB04FP1	FP	Selenium	42.5	128.7	736	3293	ng/g	1.197	22.35	RVSLSB04	4	1	St Lawrence	Smallmouth bass
RVSLSB04FP2	FP	Selenium	47.9	145.0	743	3211	ng/g	1.062	23.14	RVSLSB04	4	2	St Lawrence	Smallmouth bass
RVSLSB04FP3	FP	Selenium	44.3	134.0	761	3397	ng/g	1.149	22.40	RVSLSB04	4	3	St Lawrence	Smallmouth bass
RVSLSB04FP4	FP	Selenium	48.5	146.8	766	3296	ng/g	1.049	23.24	RVSLSB04	4	4	St Lawrence	Smallmouth bass
RVSLSB05FP1	FP	Selenium	30.6	92.4	809	3575	ng/g	1.666	22.63	RVSLSB05	5	1	St Lawrence	Smallmouth bass
RVSLSB05FP2	FP	Selenium	29.8	90.1	804	3407	ng/g	1.709	23.60	RVSLSB05	5	2	St Lawrence	Smallmouth bass
RVSLSB05FP3	FP	Selenium	31.3	94.7	803	3536	ng/g	1.626	22.71	RVSLSB05	5	3	St Lawrence	Smallmouth bass
RVSLSB05FP4	FP	Selenium	29.9	90.5	822	3519	ng/g	1.702	23.36	RVSLSB05	5	4	St Lawrence	Smallmouth bass

Sample ID	Sample Type	Analyte	MDL	QL	Selenium WW result	Selenium DW result	Se Results Units	Se Sample Weight (g)	Total Solids (%)	Site-Specimen ID	Specimen Replicate Number	Sample Replicate Number	Water Body Name	Species
GLERWA01HF1	HF	Selenium	10.3	31.1	487	2082	ng/g	4.956	23.39	GLERWA01	1	1	Erie	Walleye
GLERWA01HF2	HF	Selenium	9.6	29.2	436	1920	ng/g	5.281	22.71	GLERWA01	1	2	Erie	Walleye
GLERWA01HF3	HF	Selenium	10.3	31.0	462	2053	ng/g	4.960	22.50	GLERWA01	1	3	Erie	Walleye
GLERWA01HF4	HF	Selenium	9.6	29.0	479	2098	ng/g	5.308	22.83	GLERWA01	1	4	Erie	Walleye
GLERWA02HF1	HF	Selenium	9.9	29.8	491	2299	ng/g	5.165	21.36	GLERWA02	2	1	Erie	Walleye
GLERWA02HF2	HF	Selenium	9.7	29.4	461	2072	ng/g	5.247	22.25	GLERWA02	2	2	Erie	Walleye
GLERWA02HF3	HF	Selenium	9.6	29.1	477	2216	ng/g	5.293	21.53	GLERWA02	2	3	Erie	Walleye
GLERWA02HF4	HF	Selenium	10.0	30.2	469	2044	ng/g	5.099	22.95	GLERWA02	2	4	Erie	Walleye
GLERWA03HF1	HF	Selenium	9.6	29.0	456	1998	ng/g	5.303	22.82	GLERWA03	3	1	Erie	Walleye
GLERWA03HF2	HF	Selenium	10.4	31.4	439	1904	ng/g	4.905	23.06	GLERWA03	3	2	Erie	Walleye
GLERWA03HF3	HF	Selenium	9.6	29.2	436	1894	ng/g	5.275	23.02	GLERWA03	3	3	Erie	Walleye
GLERWA03HF4	HF	Selenium	9.8	29.5	458	2000	ng/g	5.212	22.90	GLERWA03	3	4	Erie	Walleye
GLERWA04HF1	HF	Selenium	9.8	29.7	367	1643	ng/g	5.193	22.34	GLERWA04	4	1	Erie	Walleye
GLERWA04HF2	HF	Selenium	10.3	31.1	393	1754	ng/g	4.959	22.41	GLERWA04	4	2	Erie	Walleye
GLERWA04HF3	HF	Selenium	10.3	31.1	391	1726	ng/g	4.950	22.66	GLERWA04	4	3	Erie	Walleye
GLERWA04HF4	HF	Selenium	9.7	29.4	372	1655	ng/g	5.239	22.48	GLERWA04	4	4	Erie	Walleye
GLERWA05HF1	HF	Selenium	9.8	29.8	390	1771	ng/g	5.176	22.02	GLERWA05	5	1	Erie	Walleye
GLERWA05HF2	HF	Selenium	10.1	30.4	401	1813	ng/g	5.063	22.12	GLERWA05	5	2	Erie	Walleye
GLERWA05HF3	HF	Selenium	9.9	29.8	398	1843	ng/g	5.164	21.59	GLERWA05	5	3	Erie	Walleye
GLERWA05HF4	HF	Selenium	9.8	29.6	415	1895	ng/g	5.198	21.90	GLERWA05	5	4	Erie	Walleye
GLMILT01HF1	HF	Selenium	10.3	31.3	611	1634	ng/g	4.919	37.40	GLMILT01	1	1	Michigan	Lake trout
GLMILT01HF2	HF	Selenium	9.9	29.9	660	1766	ng/g	5.151	37.37	GLMILT01	1	2	Michigan	Lake trout
GLMILT01HF3	HF	Selenium	10.2	30.9	608	1632	ng/g	4.986	37.26	GLMILT01	1	3	Michigan	Lake trout
GLMILT01HF4	HF	Selenium	10.1	30.6	578	1549	ng/g	5.036	37.31	GLMILT01	1	4	Michigan	Lake trout
GLMILT02HF1	HF	Selenium	9.7	29.4	589	1495	ng/g	5.238	39.41	GLMILT02	2	1	Michigan	Lake trout
GLMILT02HF2	HF	Selenium	10.1	30.6	684	1668	ng/g	5.037	41.01	GLMILT02	2	2	Michigan	Lake trout
GLMILT02HF3	HF	Selenium	10.1	30.6	642	1622	ng/g	5.035	39.59	GLMILT02	2	3	Michigan	Lake trout
GLMILT02HF4	HF	Selenium	9.9	29.8	630	1567	ng/g	5.166	40.20	GLMILT02	2	4	Michigan	Lake trout
GLMILT03HF1	HF	Selenium	10.3	31.1	663	1784	ng/g	4.955	37.16	GLMILT03	3	1	Michigan	Lake trout
GLMILT03HF2	HF	Selenium	10.0	30.4	642	1679	ng/g	5.067	38.23	GLMILT03	3	2	Michigan	Lake trout
GLMILT03HF3	HF	Selenium	10.4	31.3	618	1631	ng/g	4.917	37.88	GLMILT03	3	3	Michigan	Lake trout
GLMILT03HF4	HF	Selenium	10.1	30.6	647	1732	ng/g	5.035	37.35	GLMILT03	3	4	Michigan	Lake trout
GLMILT04HF1	HF	Selenium	9.8	29.7	716	2038	ng/g	5.190	35.14	GLMILT04	4	1	Michigan	Lake trout
GLMILT04HF2	HF	Selenium	9.7	29.4	734	2109	ng/g	5.245	34.81	GLMILT04	4	2	Michigan	Lake trout
GLMILT04HF3	HF	Selenium	10.0	30.3	813	2302	ng/g	5.087	35.32	GLMILT04	4	3	Michigan	Lake trout
GLMILT04HF4	HF	Selenium	10.2	30.9	821	2372	ng/g	4.985	34.61	GLMILT04	4	4	Michigan	Lake trout
GLMILT05HF1	HF	Selenium	10.1	30.6	762	2318	ng/g	5.037	32.87	GLMILT05	5	1	Michigan	Lake trout
GLMILT05HF2	HF	Selenium	9.6	29.0	788	2358	ng/g	5.316	33.42	GLMILT05	5	2	Michigan	Lake trout
GLMILT05HF3	HF	Selenium	9.6	29.1	867	2611	ng/g	5.284	33.20	GLMILT05	5	3	Michigan	Lake trout
GLMILT05HF4	HF	Selenium	9.8	29.5	823	2480	ng/g	5.216	33.18	GLMILT05	5	4	Michigan	Lake trout

Sample ID	Sample Type	Analyte	MDL	QL	Selenium WW result	Selenium DW result	Se Results Units	Se Sample Weight (g)	Total Solids (%)	Site-Specimen ID	Specimen Replicate Number	Sample Replicate Number	Water Body Name	Species
GLONSA01HF1	HF	Selenium	10.6	32.1	466	1696	ng/g	4.801	27.47	GLONSA01	1	1	Ontario	Chinook salmon
GLONSA01HF2	HF	Selenium	10.5	31.9	503	1836	ng/g	4.831	27.39	GLONSA01	1	2	Ontario	Chinook salmon
GLONSA01HF3	HF	Selenium	9.6	29.0	494	1840	ng/g	5.316	26.85	GLONSA01	1	3	Ontario	Chinook salmon
GLONSA01HF4	HF	Selenium	9.9	30.0	472	1740	ng/g	5.140	27.12	GLONSA01	1	4	Ontario	Chinook salmon
GLONSA02HF1	HF	Selenium	9.8	29.7	510	1922	ng/g	5.193	26.53	GLONSA02	2	1	Ontario	Chinook salmon
GLONSA02HF2	HF	Selenium	9.9	30.0	502	1827	ng/g	5.133	27.48	GLONSA02	2	2	Ontario	Chinook salmon
GLONSA02HF3	HF	Selenium	10.3	31.2	491	1781	ng/g	4.943	27.57	GLONSA02	2	3	Ontario	Chinook salmon
GLONSA02HF4	HF	Selenium	10.5	31.8	493	1809	ng/g	4.837	27.25	GLONSA02	2	4	Ontario	Chinook salmon
GLONSA03HF1	HF	Selenium	10.0	30.1	482	1794	ng/g	5.113	26.86	GLONSA03	3	1	Ontario	Chinook salmon
GLONSA03HF2	HF	Selenium	10.4	31.4	457	1751	ng/g	4.904	26.10	GLONSA03	3	2	Ontario	Chinook salmon
GLONSA03HF3	HF	Selenium	10.1	30.6	454	1723	ng/g	5.037	26.35	GLONSA03	3	3	Ontario	Chinook salmon
GLONSA03HF4	HF	Selenium	10.7	32.4	467	1744	ng/g	4.750	26.77	GLONSA03	3	4	Ontario	Chinook salmon
GLONSA04HF1	HF	Selenium	10.0	30.4	534	1913	ng/g	5.065	27.91	GLONSA04	4	1	Ontario	Chinook salmon
GLONSA04HF2	HF	Selenium	9.5	28.8	515	1780	ng/g	5.338	28.94	GLONSA04	4	2	Ontario	Chinook salmon
GLONSA04HF3	HF	Selenium	9.7	29.5	489	1700	ng/g	5.228	28.76	GLONSA04	4	3	Ontario	Chinook salmon
GLONSA04HF4	HF	Selenium	10.6	32.2	508	1782	ng/g	4.781	28.51	GLONSA04	4	4	Ontario	Chinook salmon
GLONSA05HF1	HF	Selenium	10.5	31.8	477	1481	ng/g	4.843	32.21	GLONSA05	5	1	Ontario	Chinook salmon
GLONSA05HF2	HF	Selenium	10.1	30.4	452	1535	ng/g	5.059	29.44	GLONSA05	5	2	Ontario	Chinook salmon
GLONSA05HF3	HF	Selenium	10.4	31.6	463	1432	ng/g	4.876	32.33	GLONSA05	5	3	Ontario	Chinook salmon
GLONSA05HF4	HF	Selenium	10.3	31.2	492	1596	ng/g	4.936	30.82	GLONSA05	5	4	Ontario	Chinook salmon
RVANBC01HF1	HF	Selenium	9.7	29.5	181	877	ng/g	5.222	20.65	RVANBC01	1	1	Anacostia	Blue catfish
RVANBC01HF2	HF	Selenium	10.4	31.5	182	871	ng/g	4.886	20.90	RVANBC01	1	2	Anacostia	Blue catfish
RVANBC01HF3	HF	Selenium	10.0	30.2	175	865	ng/g	5.102	20.22	RVANBC01	1	3	Anacostia	Blue catfish
RVANBC01HF4	HF	Selenium	10.1	30.6	172	840	ng/g	5.031	20.47	RVANBC01	1	4	Anacostia	Blue catfish
RVANBC02HF1	HF	Selenium	10.4	31.5	159	891	ng/g	4.882	17.84	RVANBC02	2	1	Anacostia	Blue catfish
RVANBC02HF2	HF	Selenium	10.5	31.8	168	925	ng/g	4.840	18.17	RVANBC02	2	2	Anacostia	Blue catfish
RVANBC02HF3	HF	Selenium	9.5	28.8	158	876	ng/g	5.341	18.04	RVANBC02	2	3	Anacostia	Blue catfish
RVANBC02HF4	HF	Selenium	9.4	28.4	163	901	ng/g	5.422	18.09	RVANBC02	2	4	Anacostia	Blue catfish
RVANBC03HF1	HF	Selenium	10.8	32.6	151	807	ng/g	4.719	18.72	RVANBC03	3	1	Anacostia	Blue catfish
RVANBC03HF2	HF	Selenium	10.6	31.9	145	750	ng/g	4.824	19.34	RVANBC03	3	2	Anacostia	Blue catfish
RVANBC03HF3	HF	Selenium	10.3	31.0	145	767	ng/g	4.965	18.91	RVANBC03	3	3	Anacostia	Blue catfish
RVANBC03HF4	HF	Selenium	9.7	29.3	147	760	ng/g	5.254	19.33	RVANBC03	3	4	Anacostia	Blue catfish
RVANBC04HF1	HF	Selenium	10.6	32.1	167	859	ng/g	4.794	19.45	RVANBC04	4	1	Anacostia	Blue catfish
RVANBC04HF2	HF	Selenium	10.3	31.1	166	843	ng/g	4.947	19.70	RVANBC04	4	2	Anacostia	Blue catfish
RVANBC04HF3	HF	Selenium	9.9	29.9	177	892	ng/g	5.146	19.84	RVANBC04	4	3	Anacostia	Blue catfish
RVANBC04HF4	HF	Selenium	10.5	31.9	178	903	ng/g	4.829	19.72	RVANBC04	4	4	Anacostia	Blue catfish
RVANBC05HF1	HF	Selenium	9.3	28.3	178	873	ng/g	5.451	20.39	RVANBC05	5	1	Anacostia	Blue catfish
RVANBC05HF2	HF	Selenium	10.3	31.1	176	854	ng/g	4.950	20.61	RVANBC05	5	2	Anacostia	Blue catfish
RVANBC05HF3	HF	Selenium	10.6	31.9	182	877	ng/g	4.822	20.76	RVANBC05	5	3	Anacostia	Blue catfish
RVANBC05HF4	HF	Selenium	9.3	28.0	174	849	ng/g	5.492	20.50	RVANBC05	5	4	Anacostia	Blue catfish

Sample ID	Sample Type	Analyte	MDL	QL	Selenium WW result	Selenium DW result	Se Results Units	Se Sample Weight (g)	Total Solids (%)	Site-Specimen ID	Specimen Replicate Number	Sample Replicate Number	Water Body Name	Species
RVPTLB01HF1	HF	Selenium	9.5	28.6	294	1471	ng/g	5.379	19.99	RVPTLB01	1	1	Potomac	Largemouth bass
RVPTLB01HF2	HF	Selenium	10.3	31.2	296	1506	ng/g	4.938	19.66	RVPTLB01	1	2	Potomac	Largemouth bass
RVPTLB01HF3	HF	Selenium	9.3	28.3	293	1452	ng/g	5.445	20.18	RVPTLB01	1	3	Potomac	Largemouth bass
RVPTLB01HF4	HF	Selenium	9.9	30.1	290	1454	ng/g	5.116	19.94	RVPTLB01	1	4	Potomac	Largemouth bass
RVPTLB02HF1	HF	Selenium	9.8	29.6	318	1640	ng/g	5.194	19.39	RVPTLB02	2	1	Potomac	Largemouth bass
RVPTLB02HF2	HF	Selenium	9.4	28.4	313	1582	ng/g	5.426	19.78	RVPTLB02	2	2	Potomac	Largemouth bass
RVPTLB02HF3	HF	Selenium	10.3	31.2	306	1501	ng/g	4.929	20.39	RVPTLB02	2	3	Potomac	Largemouth bass
RVPTLB02HF4	HF	Selenium	9.5	28.6	318	1565	ng/g	5.379	20.32	RVPTLB02	2	4	Potomac	Largemouth bass
RVPTLB03HF1	HF	Selenium	9.3	28.2	394	1896	ng/g	5.461	20.78	RVPTLB03	3	1	Potomac	Largemouth bass
RVPTLB03HF2	HF	Selenium	9.8	29.8	405	1993	ng/g	5.176	20.32	RVPTLB03	3	2	Potomac	Largemouth bass
RVPTLB03HF3	HF	Selenium	9.9	30.0	397	1851	ng/g	5.130	21.45	RVPTLB03	3	3	Potomac	Largemouth bass
RVPTLB03HF4	HF	Selenium	10.2	30.8	397	1864	ng/g	5.006	21.30	RVPTLB03	3	4	Potomac	Largemouth bass
RVPTLB04HF1	HF	Selenium	9.9	30.1	437	1854	ng/g	5.116	23.57	RVPTLB04	4	1	Potomac	Largemouth bass
RVPTLB04HF2	HF	Selenium	9.9	30.0	428	1866	ng/g	5.139	22.94	RVPTLB04	4	2	Potomac	Largemouth bass
RVPTLB04HF3	HF	Selenium	10.2	30.8	427	1863	ng/g	4.997	22.92	RVPTLB04	4	3	Potomac	Largemouth bass
RVPTLB04HF4	HF	Selenium	10.2	30.9	431	1847	ng/g	4.978	23.34	RVPTLB04	4	4	Potomac	Largemouth bass
RVPTLB05HF1	HF	Selenium	9.9	29.9	315	1519	ng/g	5.155	20.74	RVPTLB05	5	1	Potomac	Largemouth bass
RVPTLB05HF2	HF	Selenium	10.7	32.5	314	1555	ng/g	4.741	20.19	RVPTLB05	5	2	Potomac	Largemouth bass
RVPTLB05HF3	HF	Selenium	10.6	32.0	319	1558	ng/g	4.820	20.47	RVPTLB05	5	3	Potomac	Largemouth bass
RVPTLB05HF4	HF	Selenium	9.8	29.7	313	1556	ng/g	5.191	20.11	RVPTLB05	5	4	Potomac	Largemouth bass
RVSLSB01HF1	HF	Selenium	10.6	32.0	888	3647	ng/g	4.820	24.35	RVSLSB01	1	1	St Lawrence	Smallmouth bass
RVSLSB01HF2	HF	Selenium	9.8	29.8	888	3842	ng/g	5.175	23.11	RVSLSB01	1	2	St Lawrence	Smallmouth bass
RVSLSB01HF3	HF	Selenium	10.2	30.9	890	3831	ng/g	4.983	23.23	RVSLSB01	1	3	St Lawrence	Smallmouth bass
RVSLSB01HF4	HF	Selenium	10.3	31.2	900	3881	ng/g	4.941	23.19	RVSLSB01	1	4	St Lawrence	Smallmouth bass
RVSLSB02HF1	HF	Selenium	9.6	28.9	907	3973	ng/g	5.324	22.83	RVSLSB02	2	1	St Lawrence	Smallmouth bass
RVSLSB02HF2	HF	Selenium	9.6	28.9	931	3972	ng/g	5.329	23.44	RVSLSB02	2	2	St Lawrence	Smallmouth bass
RVSLSB02HF3	HF	Selenium	10.1	30.7	932	4065	ng/g	5.020	22.93	RVSLSB02	2	3	St Lawrence	Smallmouth bass
RVSLSB02HF4	HF	Selenium	10.0	30.3	901	3828	ng/g	5.088	23.54	RVSLSB02	2	4	St Lawrence	Smallmouth bass
RVSLSB03HF1	HF	Selenium	9.9	30.0	843	3790	ng/g	5.138	22.24	RVSLSB03	3	1	St Lawrence	Smallmouth bass
RVSLSB03HF2	HF	Selenium	10.5	31.6	845	3881	ng/g	4.868	21.77	RVSLSB03	3	2	St Lawrence	Smallmouth bass
RVSLSB03HF3	HF	Selenium	10.2	30.8	834	3843	ng/g	4.997	21.70	RVSLSB03	3	3	St Lawrence	Smallmouth bass
RVSLSB03HF4	HF	Selenium	10.1	30.7	834	3806	ng/g	5.023	21.91	RVSLSB03	3	4	St Lawrence	Smallmouth bass
RVSLSB04HF1	HF	Selenium	10.4	31.4	854	3963	ng/g	4.907	21.55	RVSLSB04	4	1	St Lawrence	Smallmouth bass
RVSLSB04HF2	HF	Selenium	10.5	31.6	855	3790	ng/g	4.870	22.56	RVSLSB04	4	2	St Lawrence	Smallmouth bass
RVSLSB04HF3	HF	Selenium	9.8	29.7	848	3761	ng/g	5.189	22.55	RVSLSB04	4	3	St Lawrence	Smallmouth bass
RVSLSB04HF4	HF	Selenium	10.1	30.6	829	3703	ng/g	5.027	22.39	RVSLSB04	4	4	St Lawrence	Smallmouth bass
RVSLSB05HF1	HF	Selenium	10.3	31.2	890	3823	ng/g	4.943	23.28	RVSLSB05	5	1	St Lawrence	Smallmouth bass
RVSLSB05HF2	HF	Selenium	10.3	31.2	909	3942	ng/g	4.932	23.06	RVSLSB05	5	2	St Lawrence	Smallmouth bass
RVSLSB05HF3	HF	Selenium	10.3	31.2	905	3949	ng/g	4.936	22.92	RVSLSB05	5	3	St Lawrence	Smallmouth bass
RVSLSB05HF4	HF	Selenium	10.5	31.8	910	4084	ng/g	4.839	22.28	RVSLSB05	5	4	St Lawrence	Smallmouth bass

Supplementary Information

Figure SI1. Comparison of individual fish specimen homogenized fillet (HF) and fillet plug (FP) mercury concentrations

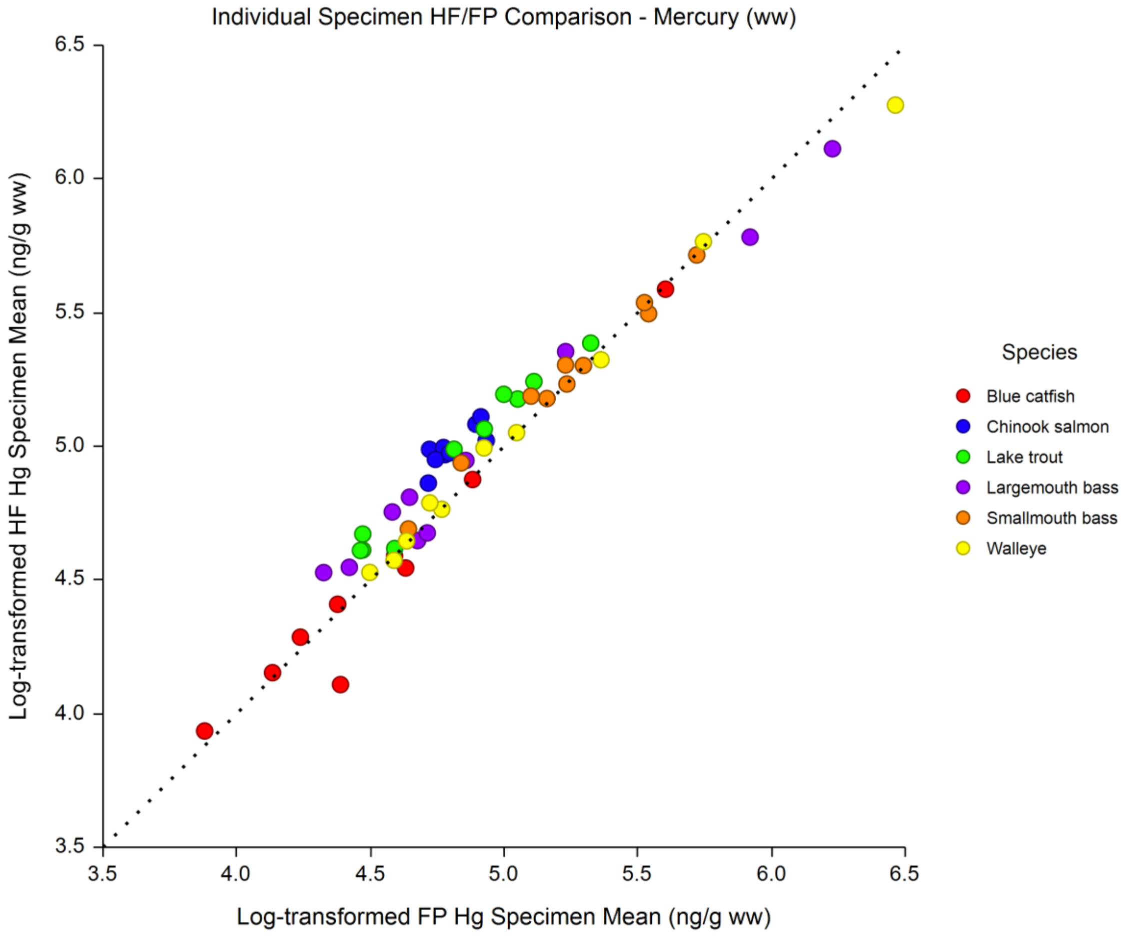
Individual Specimen HF/FP Comparison - Mercury (ww)
Log-transformed HF Hg Specimen Mean (ng/g ww)
Log-transformed FP Hg Specimen Mean (ng/g ww)
3.5
4.0
4.5
5.0
5.5
6.0
6.5
Species
Blue catfish
Chinook salmon
Lake trout
Largemouth bass
Smallmouth bass
Walleye

Supplementary Information

Figure SI2. Comparison of individual fish specimen homogenized fillet (HF) and fillet plug (FP) selenium concentrations

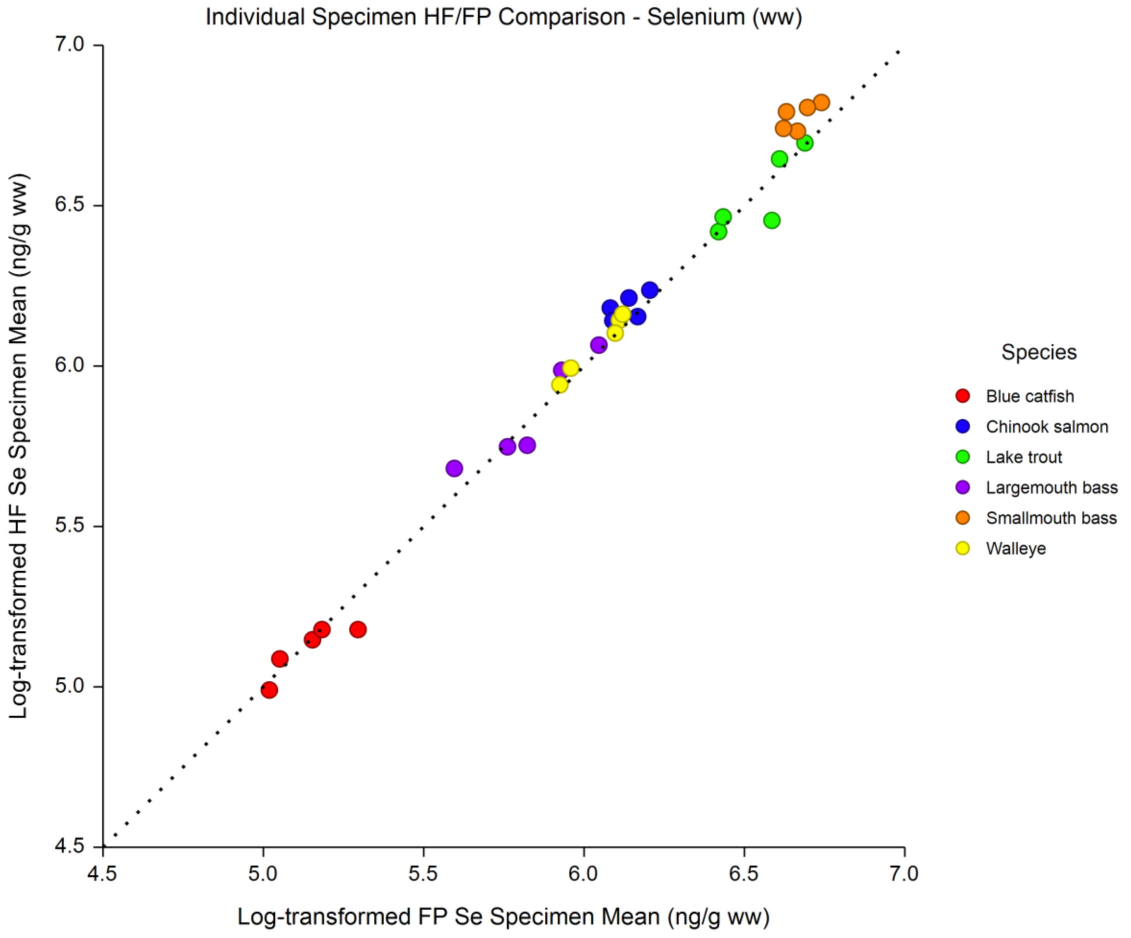
Individual Specimen HF/FP Comparison - Selenium (ww)
Log-transformed HF Se Specimen Mean (ng/g ww)
Log-transformed FP Se Specimen Mean (ng/g ww)
4.5
5.0
5.5
6.0
6.5
7.0
Species
Blue catfish
Chinook salmon
Lake trout
Largemouth bass
Smallmouth bass
Walleye
